# Artificial intelligence empowers targeted protein degradation: Core technological innovations, multi‐scenario applications, and translational prospects

**DOI:** 10.1002/smo2.70089

**Published:** 2026-08-12

**Authors:** Shuanglin Qin, Rui Peng, Guangshuai Zhang, Si Yan, Jin Xiao, Zishu Liu, Ziyu Tan, Qingqing Xie, Shangjie Li, Wei Luo, Xiaohe Xiao

**Affiliations:** ^1^ National Engineering Research Center of Personalized Diagnostic and Therapeutic Technology Research Center for Precision Medication of Chinese Medicine FuRong Laboratory Hunan University of Chinese Medicine Changsha China; ^2^ Senior Department of Hepatology China Military Institute of Chinese Materia the Fifth Medical Center of PLA General Hospital Beijing China

**Keywords:** artificial intelligence, LYTACs, molecular glues, PROTACs, structure prediction, targeted protein degradation

## Abstract

Targeted protein degradation (TPD) has emerged as a transformative therapeutic strategy that offers unprecedented opportunities to eliminate traditionally “undruggable” proteins that have posed significant challenges in traditional drug development. Current TPD approaches, including proteolysis‐targeting chimeras (PROTACs), molecular glues, and lysosome‐targeting chimeras (LYTACs), encounter several limitations. These include the complexity of forming stable ternary complexes, suboptimal design of linkers, a limited repertoire of E3 ligases, and inadequate pharmacokinetic properties. Artificial intelligence (AI) has rapidly become essential in addressing these challenges, revolutionizing the TPD drug discovery process through data‐driven insights and predictive modeling. This review systematically explores AI applications in TPD development, covering the prediction and design of stable ternary complexes, rational optimization of linkers, high‐throughput screening for E3 ligase ligands, and accurate predictions of degradation efficiency and ADMET (Absorption, Distribution, Metabolism, Excretion, Toxicity) properties. Additionally, this review underscores AI's pioneering role in discovering molecular glues, from target identification to activity prediction, and discusses the AI‐driven optimization of emerging TPD modalities, such as LYTACs and PROTAC/IMiD bifunctional molecules. Despite significant progress, several critical challenges remain, such as the absence of standardized datasets, the static modeling of dynamic biological systems, and the opaque nature of advanced AI architectures. Future research should concentrate on integrating multi‐omics data to improve model training, developing dynamic and mechanistic AI frameworks, advancing explainable AI (XAI) to enhance mechanistic interpretability, and encouraging transdisciplinary collaboration to expedite clinical translation. By integrating AI with structural biology, pharmacology, and experimental validation, TPD technologies hold the potential to expand the druggable proteome and provide novel therapeutic solutions for cancer, neurological disorders, and other persistent diseases.

## Graphical abstract

AI is transforming TPD by improving the design, prediction, and optimization of degraders such as PROTACs, molecular glues, and LYTACs. This review summarizes key AI‐driven advances, highlights applications across drug discovery stages, and discusses remaining challenges and future directions for accelerating the development of therapies against previously undruggable disease targets.

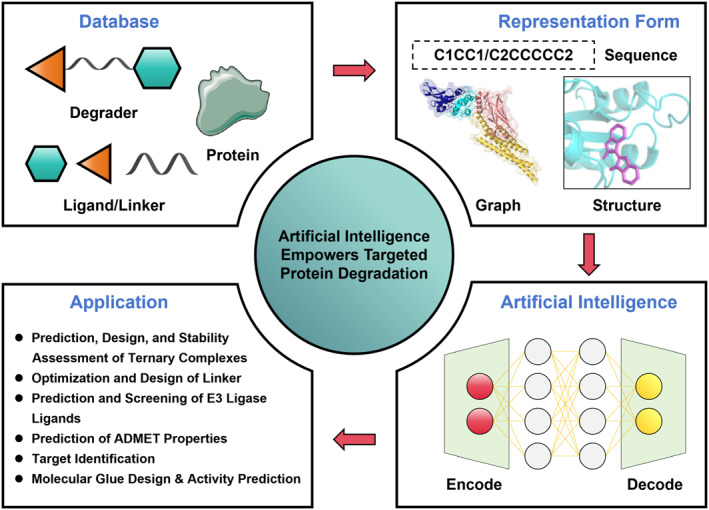

## 1 INTRODUCTION

The sphere of drug development has always been hindered by the fact that this process requires substantial investment and is fraught with significant risk.[^1^, ^2^, ^3^, ^4^] The presence of challenges in this field, particularly those known as “undruggable proteins,” represents one of the most daunting issues. These proteins are difficult to target with standard small‐molecule drug development strategies due to their structural and functional properties.[^5^, ^6^, ^7^] Often, these proteins lack stable three‐dimensional conformations and well‐defined ligand‐binding pockets, and their active sites are prone to off‐target interactions. Most are involved in complex physiological processes, making them difficult to isolate or manipulate without triggering a cascade of unwanted biological responses.^[8]^ This complexity poses a significant challenge to traditional small‐molecule drugs, which, in most cases, cannot bind these proteins selectively and effectively.[^9^, ^10^, ^11^, ^12^, ^13^]

This difficulty is further illustrated by studies on the human proteome. It has been estimated that about 2% of non‐enzymatic body proteins are essentially undruggable using conventional occupancy‐based pharmacological approaches. Moreover, it has been stated that over 80% of all human proteins lack clearly defined binding pockets that can be targeted by typical drugs. At this point, approximately 3% of human proteins in the proteome that interact with approved drugs have been identified. This vast disparity highlights one of the principal issues with the traditional drug development approach.[^14^, ^15^, ^16^, ^17^, ^18^] We need to develop new treatments that can address the vast majority of proteins that cannot be treated with existing medicines.

To address most of the existing challenges in drug discovery, scientists have adopted a relatively new methodology known as TPD.[^19^, ^20^, ^21^] TPD focuses on eliminating disease‐associated proteins, unlike traditional inhibitors that prevent the function of these proteins.[^22^, ^23^, ^24^] This innovative approach marks a significant shift in treating particularly intractable diseases. The harmful proteins are a byproduct of metabolism, and TPD fundamentally relies on the principle that selectively targets these proteins for destruction via the cell's own disposal systems, such as the ubiquitin‐proteasome system (UPS) and the lysosomal pathway. Rather than merely inhibiting their function, TPD methods completely remove these proteins, potentially leading to a long‐term therapeutic impact.[^25^, ^26^, ^27^, ^28^, ^29^] A variety of strategies have been developed within the TPD framework, including PROTACs, molecular glues, LYTACs, hydrophobic tags, and autophagy‐targeting chimeras. Currently, over 10 different approaches are actively being investigated for therapeutic use.^[30]^ TPD offers several advantages over traditional drug treatments; it is likely to be more selective and effective and operates based on a fundamentally different mechanism that involves destruction rather than inhibition. There is promising evidence from preclinical studies, both in vitro and in vivo, particularly in cancer research. The progress made has led to the idea that TPD could be used to target a significant portion of the human proteome that is otherwise untargetable.^[31]^

However, there are problems with this method. The main issue is specificity: the process of protein degradation may target critical proteins, which can have undesirable side effects or toxicities. It might also be linked to resistance, since changes can be seen at the binding sites, which makes TPD treatments less effective. Researchers are diligently working to resolve these issues so that TPD‐based medicines are safe and can be used for a long time.[^32^, ^33^, ^34^, ^35^]

**FIGURE 1:**
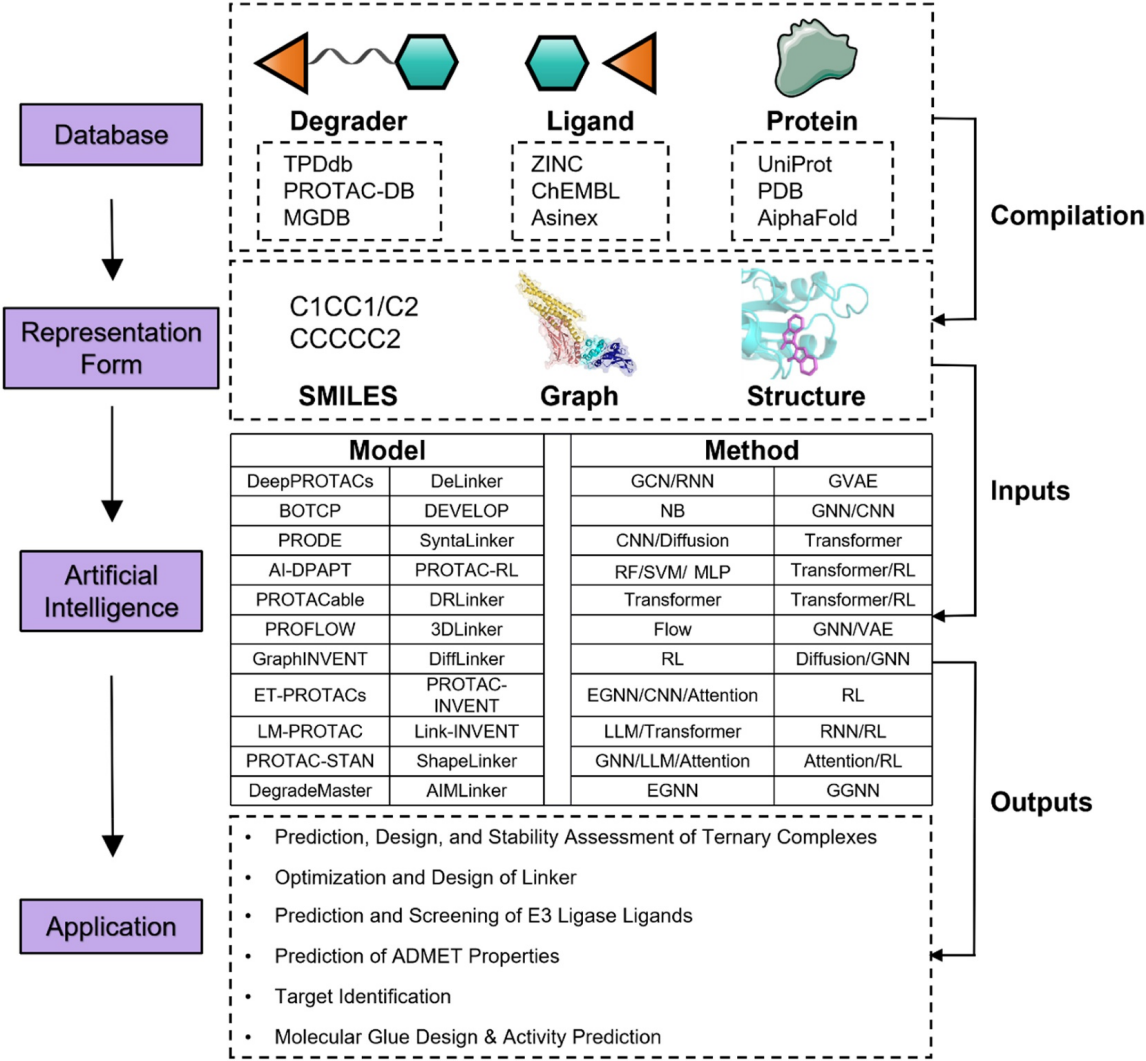
Schematic overview of AI‐assisted TPD technology. The workflow illustrates AI‐driven data processing, molecular representation, model construction, and core applications in TPD, including ternary complex prediction, linker design, E3 ligase ligand screening, ADMET prediction, molecular glue discovery, and degradation activity evaluation. AI empowers the entire TPD drug discovery pipeline by integrating multi‐modal data and advanced deep learning architectures. AI‐DPAPT, artificial intelligence‐driven PROTAC activity prediction tool; AIMLinker, artificial intelligent molecule linker; BOTCP, bayesian optimization for ternary complex prediction; CNN, convolutional neural networks; DEVELOP, deep vision‐enhanced lead optimization; EGNN, equivariant graph neural networks; GCN, graph convolutional neural networks; GGNN, gated graph neural network; GNN, graph neural networks; GVAE, geometric variational auto‐encoder; LLM, large language models; LM‐PROTAC, language model for PROTAC molecule design pipeline; MLP, multilayer perceptron; NB, naive bayes model; PRODE, PROTAC‐design‐evaluator; PROTAC‐STAN, structure‐informed deep ternary attention network framework for interpretable PROTAC degradation prediction; RF, random forest; RL, reinforcement learning; RNN, recurrent neural networks; SVM, support vector machine; VAE, variational autoencoder.

TPD technologies are increasingly being developed with substantial assistance from AI (Figure 1). By incorporating AI into the drug discovery process, researchers can overcome the challenges associated with TPD design goals while substantially reducing workflow complexity, research costs, and development times. One of the major advantages of AI is its ability to handle large volumes of data and simplify screening processes, which is essential given the complexity of TPD processes. Software for AI‐based drug discovery and design (AIDD), along with computer‐aided drug design (CADD), has improved the molecular interaction interface and identified more accurate targets for degradation.[^36^, ^37^, ^38^, ^39^, ^40^, ^41^, ^42^, ^43^] Ultimately, the synergistic benefits of AI and other advanced technologies may facilitate the clinical adoption of TPD, although it was initially only theoretical.^[44]^

## 2 CORE APPLICATIONS OF AI IN PROTAC DEVELOPMENT

**FIGURE 2:**
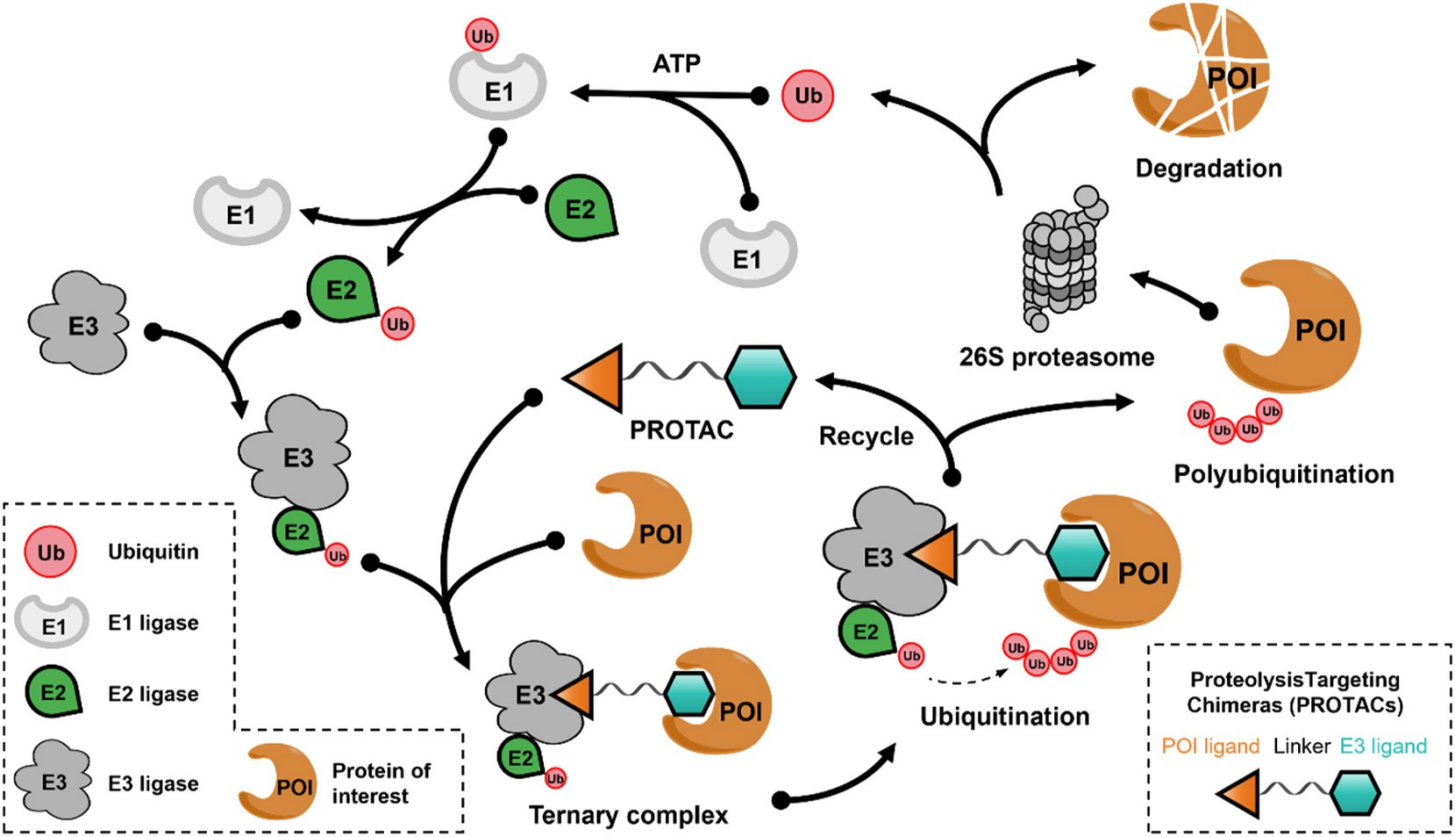
Schematic illustration of the PROTAC mechanism. PROTAC molecules recruit E3 ubiquitin ligase and POI to form a stable POI‐PROTAC‐E3 ternary complex leading to POI ubiquitination and proteasomal degradation.

PROTAC represents a novel protein regulation technology that diverges from the traditional model of protein inhibition. With its unique mechanism of action, PROTAC offers significant potential in the field of innovative drug development. The concept of PROTAC technology was first introduced by professor Craig Crews and his team at Yale University in 2001. Since then, this technology has followed a trajectory of rapid development.[^45^, ^46^, ^47^, ^48^] Drugs developed using this technology have demonstrated explosive growth in the research and development pipeline. To date, more than 30 PROTAC drugs globally have reached the clinical trial phase, addressing multiple therapeutic areas such as cancer and autoimmune diseases, while numerous others are in the preclinical research phase.[^25^, ^49^, ^50^] A typical PROTAC molecule features a heterobifunctional small molecule architecture comprising three essential components: first, a targeting ligand that specifically recognizes and binds to the protein of interest (POI); second, a ligand that forms a stable interaction with E3 ligase; and third, a linker that connects the two ligands. This design enables PROTAC molecules to uniquely facilitate the formation of a ternary complex (POI‐PROTAC‐E3 ubiquitin ligase) by allowing each ligand component, the small‐molecule ligand and the E3 ligase ligand, to bind specifically to its respective protein. This complex allows the E3 ubiquitin ligase to specifically tag the target protein with ubiquitin molecules. The ubiquitinated target protein is subsequently recognized and degraded by the cell's UPS, thus achieving precise control of the protein's level within the cell[^51^, ^52^] (Figure 2). The effective and stable formation of this ternary complex is crucial, as it is the primary requirement for initiating and efficiently advancing the degradation process. This directly influences the efficacy and specificity of PROTAC technology in modulating target proteins.[^53^, ^54^, ^55^] AI is increasingly being employed in PROTAC development to overcome these challenges. Integrating AI with structural biology can enhance our ability to predict and optimize the behavior of ternary complexes.

### 2.1 Prediction, design, and stability assessment of ternary complexes

A variety of advanced AI architectures have been developed to predict ternary complex formation, structural design, and stability validation, as exemplified by DeepPROTACs and ET‐PROTACs (Table 1). Built on GNN, Transformer, and EGNN frameworks, these computational platforms provide residue‐level and atom‐level predictions of PROTAC degradability. Although validated with multiple benchmark datasets, these methods still encounter practical limitations arising from insufficient ternary crystal structures and overlooked chiral information restricting their applicability to novel PROTAC chemical scaffolds.

**TABLE 1:** AI for prediction, design and stability assessment of ternary complexes.

Models	Molecular representations	Methods	Application scenario	Strengths	Limitations	References
DeepPROTACs	SMILES/graph	GCN/RNN	Predicts the degradation activity of ER‐targeted PROTACs recruited by VHL; generalizes to predict degradation capacity of targets including EZH2, STAT3, eIF4E, and FLT‐3	High prediction accuracy (77.95% test accuracy, 0.8470 AUROC) with multi‐module fused encoding; eliminates ternary complex construction, reduces computational cost, and supports high‐throughput virtual screening.	Ignores chirality of small molecules; limited labeled training data; generalization varies across targets due to data imbalance.	[56]
BOTCP	SMILES/structure	NB	PROTAC‐mediated ternary complex structure prediction	Sample‐efficient, fast computation, GPU‐free; accurately filter and rerank near‐native poses; suitable for unbound protein structure prediction	Poor performance on some flexible protein systems; refinement step is still computationally time‐consuming	[60]
Ribes et al.	SMILES	Linear layer/ReLU	PROTAC degradation activity prediction modeling	Open‐source, reproducible, low computational cost; multi‐embedding fusion, strong generalization	Poor generalization to novel protein targets; limited dataset scale and diversity	[61]
PRODE	SMILES	CNN/Diffusion	Rational design and evaluation of novel PROTACs; ternary complex binding mode & stability prediction	Integrated docking, MD, and free energy calculation; reproduces experimental conformations, supports novel targets	No ADMET property evaluation; lack of oral bioavailability rules	[62]
AI‐DPAPT	SMILES	RF/SVM/MLP	PROTAC activity classification prediction; rapid screening of novel PROTAC molecules	Ultra‐high prediction accuracy (AUC 0.93–0.97); multi‐model ensemble & user‐friendly web server; supports SHAP interpretable analysis	Only based on molecular fingerprints, no protein/cell info; excludes ternary complex & degradation kinetics	[63]
PROTACable	SMILES/graph/descriptors	Transformer	Automated de novo design of novel PROTACs; 3D modeling & activity prediction of ternary complexes	End‐to‐end automation, no prior ternary complex needed; SE(3)‐equivariant transformer for accurate 3D feature learning; built‐in E3/linker library, open‐source & reproducible	Insufficient accuracy for some protein‐protein docking poses; high computational cost & long runtime	[64]
PROFLOW	Graph	Flow	PROTAC‐induced ternary complex structure prediction; large‐scale virtual screening for PROTAC designs	Ultra‐fast inference; full modeling of PROTAC conformational flexibility; significant correlation with degradation activity	Relies on prior warhead binding site information; extremely scarce real ternary complex training data	[65]
GraphINVENT	Graph	RL	De novo design of novel PROTAC molecules; degrader generation for hard‐to‐drug IRAK3	Graph‐based generation supports large‐molecule de novo construction; RL‐guided design greatly improves predicted activity rate	Limited generalization due to sparse labeled data; no consideration of synthesizability and ADMET properties	[91]
ET‐PROTACs	Graph/Sequence	EGNN/CNN/Attention	PROTAC targeted degradation activity prediction; POI‐PROTAC‐E3 ternary interaction modeling	Cross‐modal fusion & ternary attention for interaction capture; EGNN leverages 3D spatial info for higher accuracy; interpretable attention visualizes key residues/atoms	Scarce ternary complex crystal structure data; cannot precisely locate binding sites	[66]
AIMLinker	SMILES/Graph	GGNN	De novo linker design for PROTACs; stable conformation generation for ternary complexes	Preserves 3D spatial structure information; multi‐index screening ensures drug‐likeness	Relies on accuracy of protein‐ligand superimposition; insufficient conformational sampling due to short simulation time	[67, 68]
LM‐PROTAC	Graph/Sequence	LLM/Transformer	De novo generation of PROTAC degraders for target proteins; targeted degradation design for disease‐related proteins	Dual constraints of structure and physicochemical properties; language model‐driven, automated end‐to‐end design; strong generalizability, fast adaptation to new targets	Low molecular generation efficiency; insufficient diversity due to similar skeletons; limited accuracy of protein‐fragment affinity prediction	[69]
PROTAC‐STAN	Graph/sequence	GNN/LLM/Attention	Interpretable prediction of PROTAC degradation activity; POI‐PROTAC‐E3 ternary interaction analysis	Integrates protein structure and molecular hierarchical representation; explicit ternary interaction modeling via ternary attention; atomic/residue‐level visualization, high interpretability	Limited generalization due to few rigid linker data; relies on protein sequence‐structure pre‐trained models	[70]
DegradeMaster	Graph/descriptors	EGNN/Semi‐supervised learning	Accurate prediction of PROTAC‐targeted degradation activity; degradability evaluation of mutant proteins	E(3)‐equivariant encoding preserves 3D spatial structure; semisupervised pseudolabeling uses massive unlabeled data; mutual attention pooling with high interpretability	Only binary classification, no continuous DC_50_ output; 3D modeling leads to slightly higher inference time	[71]

*Note*: Further elucidation is readily accessible within the pertinent bibliographic citations.

Abbreviations: GraphINVENT, graph‐based deep generative model; MD, molecular dynamic; ReLU, rectified linear unit.

In 2022, Li et al. introduced DeepPROTACs (Table 2, 1), a deep learning‐based model for predicting PROTAC degradation activity, addressing the previously unclear structure‐activity relationships in the rational design of PROTAC molecules^[56]^ (https://github.com/fenglei104/DeepPROTACs & https://bailab.siais.shanghaitech.edu.cn/ services/deepprotacs/). The research team amassed a dataset of 2832 PROTAC records from databases such as PROTAC‐DB.[^57^, ^58^, ^59^] They categorized these records into 988 “good degraders” and 1844 “poor degraders,” based on criteria of DC_50_ ≤ 100 nM and D_max_ ≥ 80%. The model employs a multi‐module neural network architecture that includes the POI protein pocket, the E3 ligase pocket, the target protein ligand (warhead), and the E3 ligand, all of which are analyzed using GCN. The linker SMILES sequence is encoded via a bidirectional LSTM. These four‐component features are then concatenated and fed into an MLP for binary classification prediction. The model achieved an accuracy of 77.95% and an AUROC of 0.8470 on the test set, markedly surpassing traditional ML methods. To assess the model's practicality, the team designed 16 PROTAC molecules targeting the estrogen receptor (ER), incorporating alkyl chains and PEG linkers of various lengths. Experimental results indicated that 11 of these molecules were effective degraders. DeepPROTACs accurately predicted the efficacy of these 11 molecules, achieving a prediction accuracy of 68.75%. The prediction accuracy for new targets, such as EZH2 and STAT3, ranged from 65% to 80%, demonstrating strong generalization capabilities. Computational modeling suggested that a linker of medium length (approximately 8 Å) forms the most stable ternary complex, enhancing the recognition of lysine residues on the target protein surface by the ubiquitination system, thereby improving degradation efficiency. DeepPROTACs provide an effective tool for the high‐throughput virtual screening of PROTAC drugs, potentially reducing experimental costs and accelerating the development cycle.

**TABLE 2:** Examples of AI‐generated PROTAC molecules.

Structures	Bioinformatics
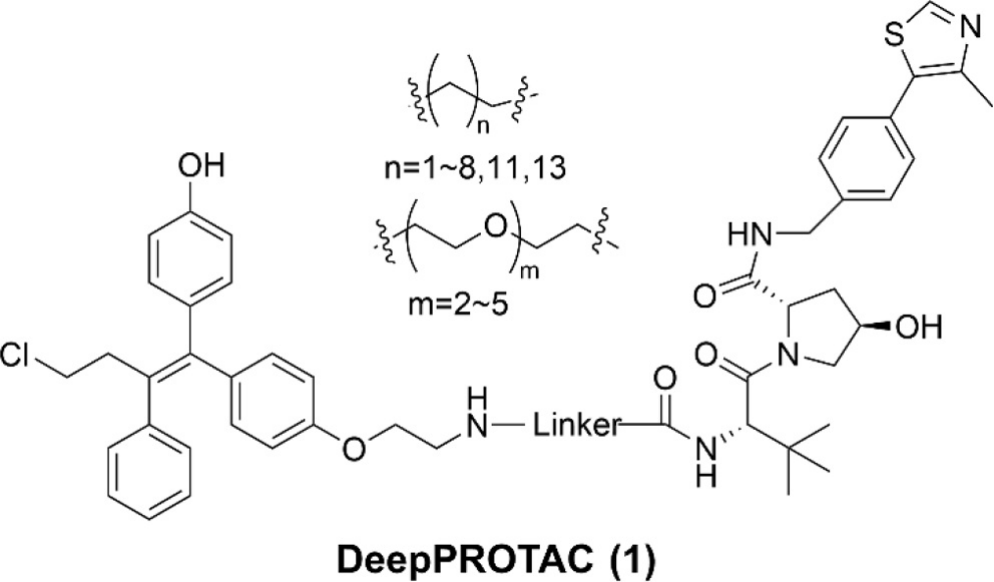	POI: ER E3 ligand: VHL POI ligand: Toremifene derivative linker: Alkyl chain/PEG chain Activity: DC_50_ ≤ 100 nM, D_max_ ≥ 80% Cell model: MCF‐7, T‐47D cells
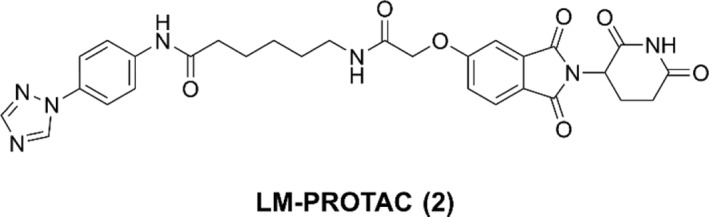	POI: Wnt3a E3 ligand: CRBN linker: Alkyl chain Activity: Dose‐dependent degradation at 0.16–5 μM Cell model: HepG2 cells
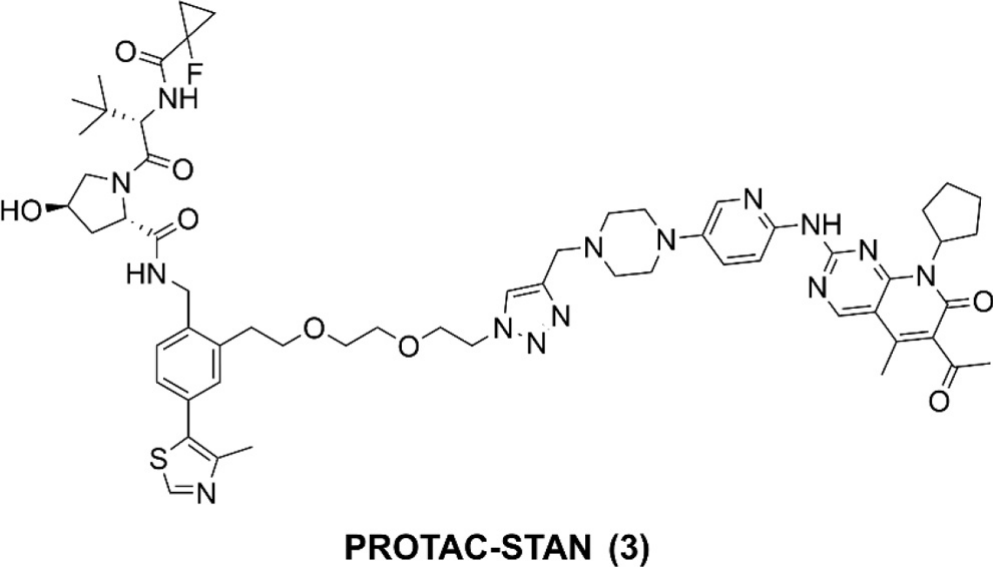	POI: CDK4 E3 ligand: VHL linker: PEG chain Activity: DC_50_ ≤ 100 nM, D_max_ ≥ 80% Model accuracy: 88.41% AUROC = 0.8833 F1 score = 0.8588 Average lysine SASA: ∼70 Å^2^
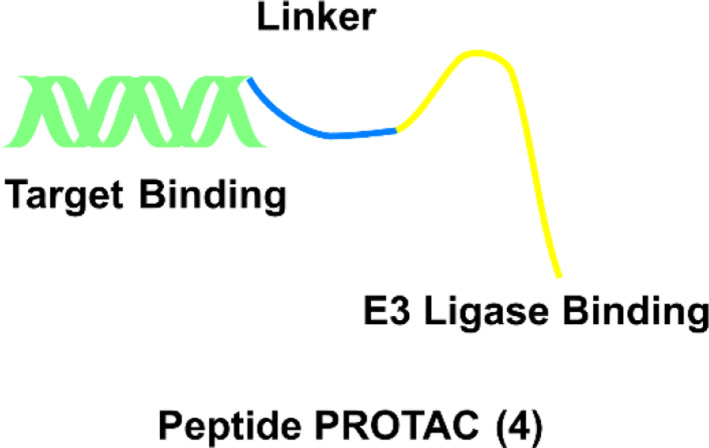	POI: AR E3 ligand: CRBN Full sequence: 116 nM AR‐binding peptide: SSKLRQLLF VHL‐binding peptide: YGEGTSSFWL Linker: GGSGG C‐terminal modification: GGC Binding affinity to AR: 19.4 nM Binding affinity to VHL: 57.8 nM DC_50_ (C4‐2 cells): 693.1 nM DC_50_ (LNCaP cells): 892.5 nM
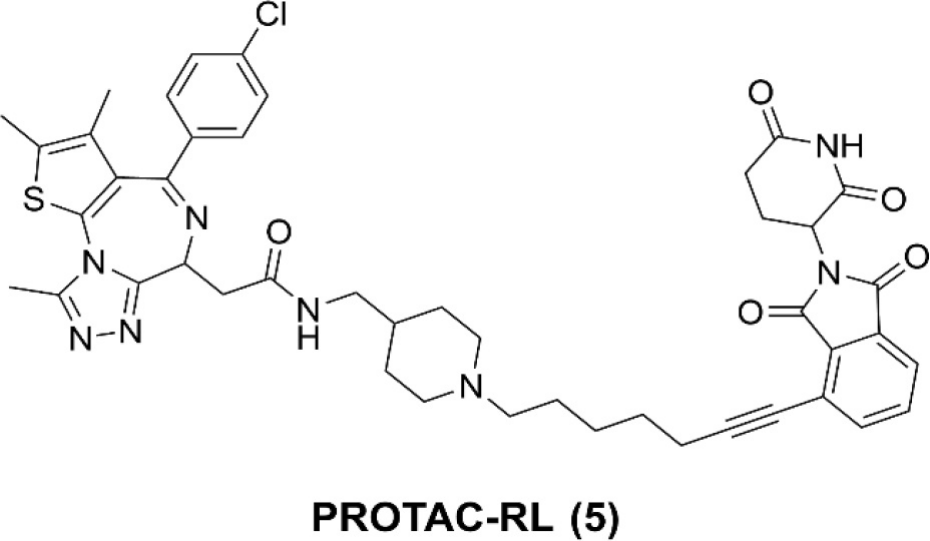	POI: BRD4 E3 ligand: VHL IC_50_ (Molt4 cells): 116 nM C_max_ = 194 ng/mL T_1/2_ = 2.22 h AUC_0‐inf_ = 319 ng ・h/mL
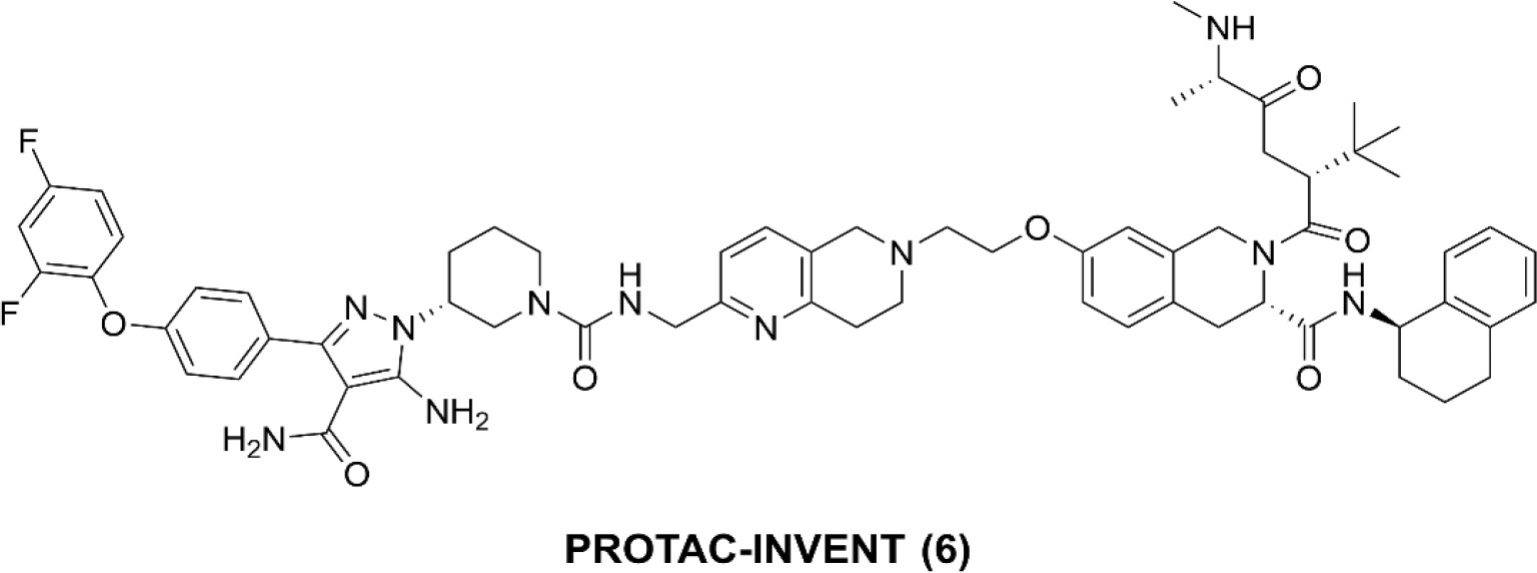	POI: BTK E3 ligand: cIAP1/BIRC2 Docking score: −10.9 Best RMSD: 0.157 Å Docking score < −10.9: Up to 72.8% Ps > 0.9: Up to 71.5%
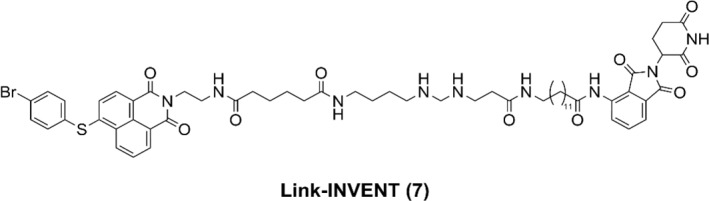	POI: Bcl‐2/Mcl‐1 E3 ligand: CRBN tPSA ≤ 250 Å^2^ logP: 3.5–6.0 Number of hydrogen bond acceptors ≤16 Number of hydrogen bond donors ≤6 Number of rotatable bonds <25
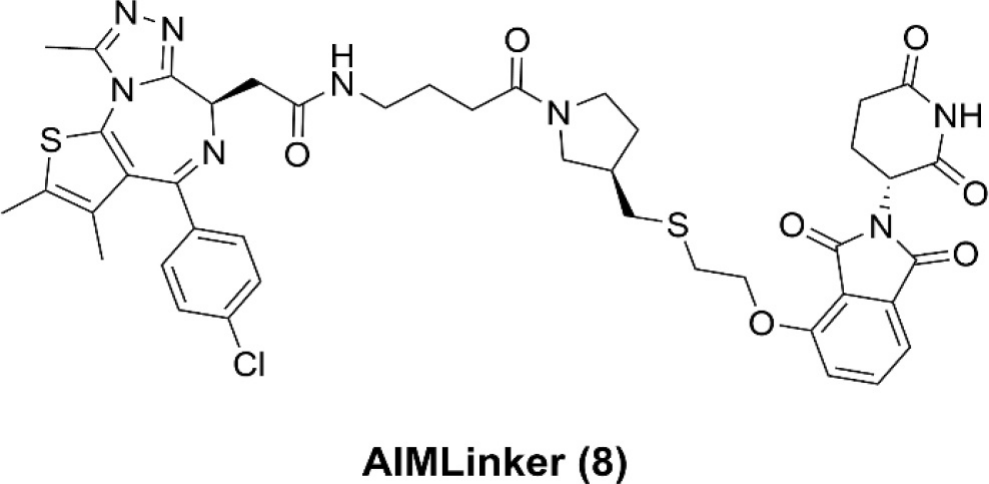	POI: BRD4 E3 ligand: CRBN Average RMSD: 0.44 ± 0.05 Å Docking ΔG binding: −62.26 ± 6.36 kcal/mol 10 ns MD ΔG binding: −45.91 ± 4.78 kcal/mol ΔΔG binding versus crystal: −0.74 kcal/mol FEP ΔΔG binding versus crystal: −1.25 ± 0.85 kcal/mol
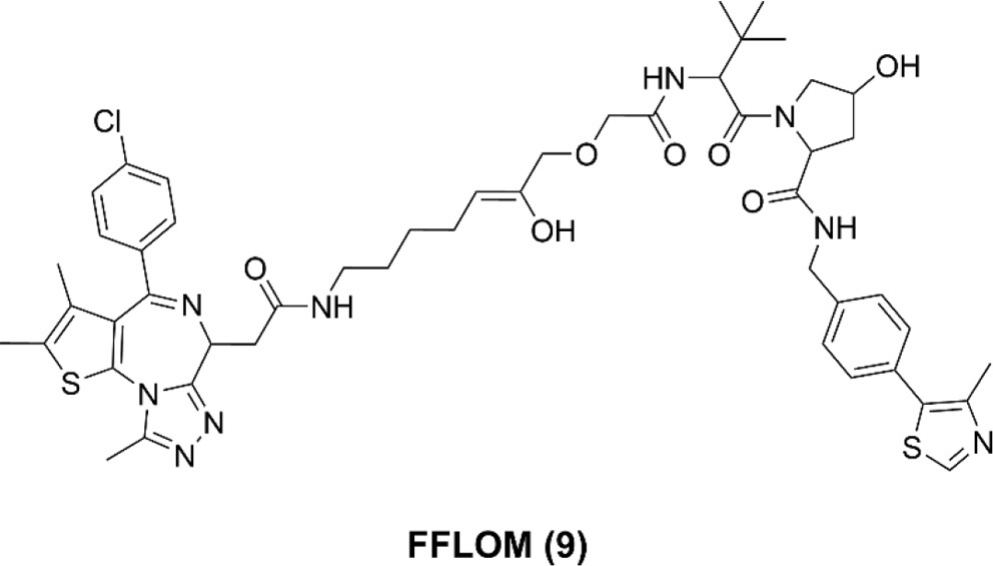	POI: BRD4 E3 ligand: VHL Molecular docking energy: −17.13 kcal/mol
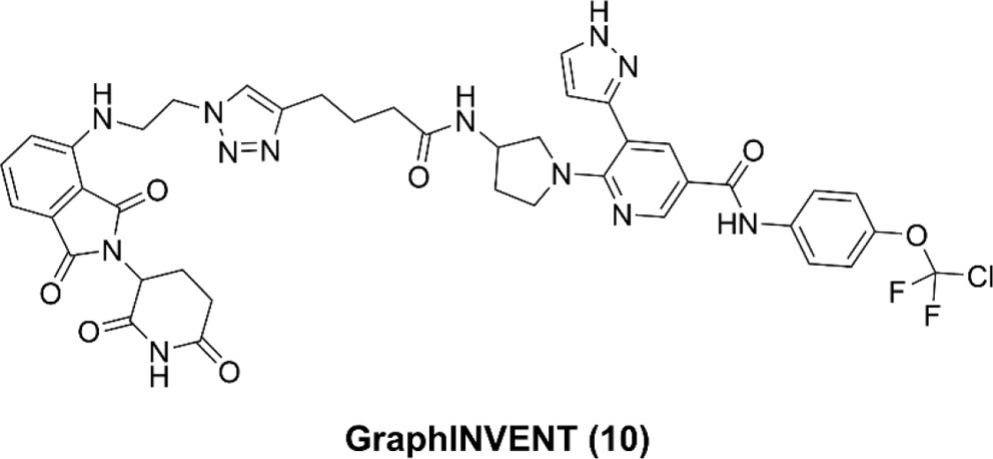	POI: IRAK3 E3 ligand: CRBN DC_50_ < 100 nM

*Note*: Illustrating diverse structural designs and biological activities derived from different AI models.

Abbreviations: AR, androgen receptor; Bcl‐2, B‐cell lymphoma 2; BRD4, Bromodomain containing 4; BTK, Bruton's tyrosine kinase; CDK4, Cyclin dependent kinase 4; IRAK3, Interleukin‐1 receptor‐associated kinase 3; Mcl‐1, Myosin regulatory light chain 1; Wnt3a, Wnt family member 3A.

In 2023, Rao et al. proposed a new BOTCP aiming to address the primary structural prediction challenges in the development of TPD drugs.^[60]^ PROTACs promote protein degradation by facilitating the formation of a ternary complex involving the target protein and E3 ubiquitin ligase. However, traditional computational methods struggle with issues such as extensive conformational space and complex interactions. These methods typically depend on experimental structures or are computationally intensive, posing difficulties for efficient application in the early stages of drug design. The BOTCP method achieves sample‐efficient exploration of this complex conformational space through an ML‐driven active learning strategy. The methodology encompasses five stages: initially, inputting the unbound structures of the POI and E3 ligase, along with PROTAC molecules; secondly, employing Bayesian optimization within the 7D roto‐translation space, using protein interaction energy (DFIRE) and PROTAC conformational feasibility as the joint objective function, while iteratively sampling approximately 2500 candidate conformations; thirdly, optimizing the PROTAC stability score using a simulated annealing algorithm (constrained docking based on an improved AutoDock Vina); fourthly, filtering out unreasonable conformations using protein interaction constraint energy (TCP‐AIR) and reordering the clustering results; finally, refining the predictions through MD. Two innovative scoring indicators, TCP‐AIR energy and PROTAC stability score, ensure the rationality of protein spatial arrangement and ligand conformational stability, respectively. In the evaluation of 14 PDB ternary complexes (22 test cases), BOTCP's prediction performance on unbound structures substantially surpassed that of existing methods. Before refinement, 21 out of 22 cases achieved acceptable quality (DockQ ≥0.23), with 16 cases ranking in the top 20, and 12 reaching medium quality; after refinement, all cases obtained acceptable structures, 17 were ranked in the top 20, 14 achieved medium quality, and 2 reached high quality. Compared to methods like PRosettaC that rely on bound structures, BOTCP requires only about 2 h on a 128‐core CPU to complete the predictions without GPU acceleration, thus improving computational efficiency by several orders of magnitude. This method provides an efficient and reliable structural screening tool for the rational design of PROTACs with potential for further enhancements in prediction accuracy by integrating side chain flexibility and entropy effects in the future.

In 2024, Ribes et al. proposed an ML‐based PROTAC degradation activity prediction framework to address the problems of scarce data and insufficient tools in PROTAC research and development^[61]^ (https://github.com/ribesstefano/PROTAC‐Degradation‐Predictorr). The research team compiled 2141 experimental data points from two open‐source databases, PROTAC‐DB[^57^, ^58^, ^59^] and PROTAC‐Pedia, covering essential information such as pDC_50_, D_max_, E3 ligase, POI sequence, and cell line. They established an activity threshold of pDC_50_ ≥ 6 and D_max_ ≥ 60% for binary classification labeling. They adopted a multi‐modal embedding strategy, generating 256‐bit Morgan fingerprints of PROTAC molecules through RDKit, obtaining 1024‐dimensional UniProt protein embeddings of POI and E3 ligase using pre‐trained Transformers, and performing one‐hot encoding on cell lines. These features were aggregated after independent linear layers and softmax normalization, and then used to achieve activity prediction through fully connected layers and ReLU activation functions. This work is the first to predict PROTAC activity by integrating DC_50_ and D_max_ as dual indicators, which is more practical and reproducible than existing methods such as DeepPROTACs. Its open‐source toolkit lowers the computational threshold and provides a new benchmark for the rational design of PROTACs, while also highlighting the importance of developing richer protein representations and expanding datasets to improve target generalization ability.

Geofrey et al. proposed an innovative computer‐aided PROTAC design and evaluation method, PRODE, aimed at addressing the bottleneck caused by the lack of systematic computational tools for the rational design of existing PROTACs^[62]^ (https://github.com/rdkit/rdkit). PROTACs induce protein degradation by linking the target protein to an E3 ligase; however, traditional methods struggle to accurately predict the structure and binding affinity of the resulting ternary complex. PRODE integrates cutting‐edge technologies such as protein‐protein docking, torsion diffusion conformational sampling, MD simulation, and binding free energy calculation. This integration establishes a comprehensive workflow from linker selection to complex stability assessment and introduces thermo‐titration molecular dynamics (TTMD) technology to distinguish the stability of complexes with varying binding constants. The effectiveness of PRODE was initially verified using the BRD4‐VHL system (PDB ID: 8BDX). MEGADOCK was employed for protein‐protein docking to screen for linker‐compatible conformations. The torsion diffusion algorithm generated 4000 PROTAC conformations, which were aligned based on templates, successfully reproducing the crystal binding mode of PROTAC 48 (RMSD = 2.084 Å). A 50‐ns MD simulation demonstrated that the complex has good stability, and MMPBSA calculations indicated that the free energy of the ternary complex (ΔG_TER = −88.29 kcal/mol) was substantially lower than that of the binary complexes (ΔG_BI = −39.39 and −29.34 kcal/mol), confirming a synergistic effect. Subsequently, this method was applied to the novel FGFR1‐MDM2 system to design degraders targeting the cancer‐related FGFR1. The candidate molecule with linker 184 was selected, achieving a stable ternary complex structure (ΔG_TER = −83.29 kcal/mol). TTMD verification showed that the ternary complex with a low Kd value (8BDT, 0.006 μM) retained markedly more interactions (0.866) at high temperatures than the system with a high Kd value (8BDX, 0.043 μM; 0.644). This trend was repeated in another system (7JTP vs. 7JTO), proving that TTMD can effectively distinguish the stability of PROTAC‐mediated complexes. The PROTAC_184 designed for the FGFR1‐MDM2 system still retains key hydrogen bonds and hydrophobic interactions at 450K. PRODE provides an efficient computational platform for early PROTAC discovery, considerably shortens the traditional DMTA cycle, and shows potential to facilitate the development of TPD drugs.

Abouzied et al. developed an AI‐driven PROTAC activity prediction tool, AI‐DPAPT, to address the challenge of predicting PROTAC molecule activity in TPD technology^[63]^ (https://ai‐protac.streamlit.app/). The research team utilized ML algorithms and molecular fingerprint technology to create a computational platform that quickly predicts PROTAC activity based on chemical structures. This platform aims to enhance the screening and optimization of candidate drugs. Technically, AI‐DPAPT employs a diverse PROTAC dataset from databases such as PROTAC‐DB 2.0. It converts SMILES structures into 1024‐bit Morgan molecular fingerprints using RDKit and utilizes LabelEncoder and StandardScaler for feature encoding and standardization. The team trained and evaluated five classification models: RF, gradient boosting, AdaBoost, SVM, and MLP, with ROC‐AUC as the performance indicator. The results indicated that the AUC of all models exceeded 0.9, with Random Forest achieving the highest test set AUC of 0.97, dramatically outperforming existing tools such as DeepPROTACs, which scored 0.847. Through case studies of clinically validated PROTAC molecules, such as ARV‐110 and ARV‐471, as positive and negative controls, AI‐DPAPT demonstrated high accuracy in distinguishing active from inactive compounds. This study shows that AI‐DPAPT provides a reliable, efficient, and user‐friendly computational framework for PROTAC drug design, substantially contributing to rational drug design in the field of TPD.

PROTACable is a computational pipeline for automated PROTAC design that integrates three‐dimensional structure modeling and deep learning^[64]^ (https://github.com/giaguaro/PROTACable/). Mslati and colleagues categorized PROTAC molecules into functional modules by constructing a chemical library containing 86 E3 ligase ligands, 262 target protein ligands, and 488 linkers. They sequentially performed small molecule docking, protein‐protein docking, linker screening and reconstruction, and ultimately generated candidate ternary complex structures through energy minimization. Employing SE(3) equivariant graph Transformer networks, the model effectively identifies key structural features independent of spatial orientation using three‐dimensional atomic coordinates and topological information and predicts PROTAC in vitro activity by combining molecular fingerprints and physicochemical descriptors. Focusing on the histone methyltransferase G9A, PROTACable automatically generated more than 1000 candidate molecules from the inhibitor UNC0642, with the top 20 molecules, all containing VHL‐type E3 ligase, confirming its practical value. The platform, built entirely on open‐source tools, enables complete process automation from molecular design to activity prediction by simply inputting the target protein structure, providing an efficient, accessible, and low‐cost solution for PROTAC drug research and development.

PROFLOW is the first end‐to‐end deep learning model designed to predict the structure of PROTAC‐induced ternary complexes^[65]^ (https://www.rdkit.org). PROTACs facilitate protein degradation by simultaneously binding to a target protein and an E3 ligase. However, their rational design has lacked a structural basis, causing existing algorithms to oversimplify the process into a mere protein docking problem constrained by distance. To address this, the authors introduced a pseudo‐data generation scheme based on binary protein complexes and PROTAC linker graphs, which enabled the construction of a large‐scale training set that takes full account of linker flexibility. The framework employs a flow matching iterative optimization strategy: it defines a linker‐compatible SE(3) transformation space, generates a prior distribution of E3 ligase from linker conformational sampling, interpolates on the SE(3) manifold, projects back to the compatible space, and trains an E3NN equivariant network to predict rigid body transformations. Unlike traditional methods that reduce linkers to mere distance constraints, PROFLOW models their entire conformational space throughout the process, thereby facilitating accurate docking under the constraints of chemical information. Experiments demonstrate that PROFLOW outperforms existing methods in both holo/apo structure re‐docking, achieving an interface RMSD as low as 8.20 Å and an Fnat of 0.264, and it operates 60 times faster than previous approaches. A large‐scale screening of PROTAC‐DB reveals that the predicted change in solvent‐accessible surface area strongly correlates with DC_50_ (*r* = −0.52). Additionally, case studies confirm a negative correlation between Rosetta energy and D_max_ (*r* = −0.60), thus validating the model's practical value in predicting degradation activity and offering an efficient computational tool for the rational design of PROTACs.

Cai et al. proposed the ET‐PROTACs model, which is the first to integrate cross‐modal learning with a ternary attention mechanism^[66]^ (https://github.com/GuanyuYue/ET‐PROTACS). For PROTAC molecules, RDKit constructs a three‐dimensional graph structure, while EGNN extracts geometric features that are invariant to rotation and translation. A three‐layer CNN captures local amino acid patterns in the POI/E3 protein sequences. The model's ternary attention module models the spatial‐channel interactions between the POI and PROTAC, as well as between the E3 and PROTAC, through cross‐channel attention matrices. It uses an MLP to generate attention vectors and calculate weighted fusion representations, finally concatenating them to predict degradation activity using a classifier. This method innovatively models three‐dimensional structural information and sequence information synergistically and quantifies the contribution of each component. On the self‐built 1086‐sample PROTAC‐DB dataset, ET‐PROTACs comprehensively outperform benchmark models with an accuracy of 0.853 and an AUC of 0.781. In ablation experiments, EGNN's three‐dimensional structure extraction proved superior to two‐dimensional methods such as GCN/GAT. CNN is more effective than the Transformer on small samples, and the attention module increases the AUC by 7.9%. Attention visualization shows that when PROTAC 22 degrades BRD9, significant weights are located at positions 109–113 (the binding pocket) and 576–580 (a distal site) of the target protein. E3 (VHL) exhibits a global attention pattern, and the warhead region (atoms 8/14/24) and linker region of PROTAC concentrate high weights, verifying the biological interpretability of the model. In the future, semi‐supervised learning will be used to expand the data, binding site annotation will be introduced to improve positioning accuracy, and stronger protein encoding methods will be explored to break performance limits.

To address the challenges of limited crystal structures and the difficulty in constructing ternary complexes in PROTAC drug research and development, Lin and colleagues proposed an innovative rational PROTAC design method that combines structure superposition with deep neural networks.^[67]^ The AIMLinker^[68]^ (https://github.com/AnHorn/AIMLinker) model developed in this study utilizes GGNN within the VAE framework. It integrates over 160,000 molecular data points from the ZINC database and PROTAC‐DB. This model can generate linking fragments based on the three‐dimensional spatial information of POI and E3 ligands and filters out non‐drug‐like structures through post‐processing. The workflow employs the BRD4‐AT7‐VHL ternary complex as a benchmark, superimposes the BRD4‐AT7 pair onto the new BRD4‐C18/MS417 ligand pair, retains the VHL ligand as an anchor point, and inputs it into the network. Through multiple rounds of CDOCKER docking, RMSD structural similarity, binding free energy (BFE), and buried solvent‐accessible surface area (SASA), the screening for the generated 220–224 candidate molecules is achieved. The final candidate molecules, C18‐02‐0019 (RMSD = 1.20 Å, ΔG_BFE = −43.71 kcal/mol) and MS417‐01‐0157 (RMSD = 0.69 Å), demonstrate better binding affinity than the reference molecule, AT7. A 10‐ns MD simulation indicates that C18‐02‐0130 exhibits the best number of non‐bonded interactions (36.73) and ΔRg (0.13 Å). Free energy perturbation (FEP) calculations further confirm that its relative binding free energy (−1.91 kcal/mol) is substantially superior to that of AT7. To verify the generality of the method, the study was repeated with the cIAP1‐BTK system, where the newly generated molecules also displayed higher BFE and reasonable SASA values. This method overcomes data limitations through structural alignment, captures molecular spatial relationships using deep learning, establishes screening criteria of RMSD‐BFE‐SASA, and achieves efficient rational design.

In 2025, the Shao team proposed a new language model‐based PROTAC molecule generation method, LM‐PROTAC (Table 2, 2), which addresses the limitations of traditional computer‐aided drug design in developing protein degraders by imposing dual constraints on structure and physicochemical properties^[69]^ (http://zinc15.docking.org). Utilizing natural language processing technology, this method segments molecules and proteins into functional fragments (S‐mol/S‐pro), employs the FOTF‐CPI model to identify fragment pairs with high affinity for the target protein, and then uses the DCT generation model, which combines C‐Transformer and RL, to simultaneously constrain molecular skeleton similarity and key pharmacokinetic properties, such as solubility and lipophilicity, during the generation process. This approach realizes the efficient, directed generation of complete PROTAC molecules from fragments. The technical workflow comprises four core modules: initially, chemical rule filtering and VOLT algorithm segmentation are applied to molecules from the ZINC database to construct an S‐mol library of more than 8.7 million; next, the FOTF‐CPI prediction model is trained, achieving an AUC of 0.929 on datasets such as BindingDB and dramatically surpassing methods like DeepDTA, and identifies 9 pairs of SSIs with high affinity for the liver cancer‐related target Wnt3a; subsequently, the DCT model generates 10,000 candidate molecules using these fragments as structural constraints and LogP/LogS as property constraints, and 372 molecules pass drug similarity (QED), SAS, and Lipinski's Rule of Five screening through the MDAM multi‐dimensional attribute model, which integrates 1D/2D/3D features; finally, 12 molecules undergo retrosynthetic analysis, and 3 complete chemical synthesis. In vitro experimental verification shows that Compound 1 exhibits dose‐dependent degradation of the Wnt3a protein in HepG2 cells in the range of 0.16–5 μM, with an IC_50_ of approximately 811.96 nM, while Compound 2 shows weak activity. MD simulation confirms that the three candidate molecules form stable bindings with the Wnt3a‐CRBN complex, among which Compounds 1 and 3 exhibit the smallest RMSD fluctuations and markedly reduced SASA. The entire process from design to verification takes only about 50 days. By segmenting molecules from an NLP perspective and using an integrated generation‐screening strategy, this method substantially enhances the efficiency of PROTAC development, providing an automated solution for targeting “undruggable” proteins.

In addition to task‐specific deep‐learning architectures such as LM‐PROTAC, biological foundation models and LLMs have rapidly emerged as transformative tools for modern TPD molecular design. These foundation models are pre‐trained on extensive datasets comprising millions of protein sequences, AlphaFold‐generated 3D structures, and diverse small‐molecule SMILES strings, enabling zero‐shot and few‐shot generation of novel PROTAC candidates without extensive labeled degradation data. Representative foundation models, including BioGPT and ChemBERTa, facilitate target‐specific PROTAC design through transfer learning after fine‐tuning on curated PROTAC‐DB and TPDdb datasets. When combined with equivariant diffusion‐based generative frameworks, these foundation models concurrently produce rational molecular topologies and optimized 3D conformations, significantly reducing computational demands during ternary complex conformational screening. Nonetheless, most currently available foundation models lack pre‐training on TPD‐specific ternary interaction data, limiting their generalization to unconventional E3 ligase recruiters and novel, undruggable targets.

Chen et al. proposed a novel structure‐aware deep ternary attention framework named PROTAC‐STAN (Table 2, 3) to address several key challenges in existing deep learning methods for PROTAC degradation prediction. These challenges include the overlooking of molecular hierarchical structure information, the underutilization of protein structure data, and the lack of interpretability in “black box” models^[70]^ (https://github.com/PROTACs/PROTAC‐STAN http://cadd.zju.edu.cn/protacdb/). By incorporating an atomic‐molecular‐property hierarchical characterization of PROTAC molecules, the structure embedding of the POI and E3 ligase based on protein language models, and an innovative ternary attention network, this framework successfully models the interactions within the ternary complex explicitly. The ternary attention mechanism of PROTAC‐STAN not only captures the complex interactions among the POI, PROTAC, and E3 ligase simultaneously but also generates quantifiable three‐dimensional attention maps that visualize the prediction results down to the atomic and amino acid residue levels. On the PROTAC‐fine dataset, which contains 1503 samples, the model achieves an accuracy of 88.41%, an AUROC of 0.8833, and an F1 score of 0.8588, surpassing the best baseline by over 10%. In the study, key molecular interaction regions were found through attention weight mapping. As indicated by the results, the weak protein‐protein interaction between E3 and POI markedly affects the degradation efficiency. The framework exhibits excellent generalization capabilities: it maintains an accuracy of over 70% in out‐of‐distribution testing on the PROTAC‐DB 3.0 dataset. Linker sensitivity analysis confirms its ability to differentiate among various chemical structures. Case studies targeting the CDK4 protein, verified through MD simulations, show that all highly active PROTACs predicted by the model exhibit stable protein‐protein binding energies (ranging from −39.25 to −20 kcal/mol) and conformational stability. Ablation experiments reveal that hierarchical encoding and the ternary attention mechanism contribute 4.87% and 6.77% to the performance improvement, respectively.

Liu et al. proposed DegradeMaster, a semi‐supervised E(3)‐EGNN designed to predict PROTAC‐TPD ability. This model addresses two significant shortcomings of existing deep learning methods: the omission of 3D spatial information and dependence on limited labeled data^[71]^ (https://github.com/ABILiLab/DegradeMaster; https://zenodo.org/records/14715718). DegradeMaster incorporates five core components: a 3D molecular graph construction module, which treats atoms as nodes, chemical bonds as edges, and incorporates three‐dimensional coordinates; an E(3)‐equivariant encoder that maintains consistent molecular representations under transformations such as rotation and translation through equivariant coordinate transformation and feature aggregation; a feature selection module that identifies six key chemical descriptors; a cross‐attention pooling mechanism that calculates the contribution weight of each node to the degradation prediction, thereby enabling interpretability analysis; and a memory‐enhanced pseudo‐label strategy that combines prediction divergence with the similarity between unlabeled samples and labeled prototypes to select high‐quality pseudo‐labels for expanding the training set. On the PROTAC‐1K and PROTAC‐8K datasets, which contain 1503 and 8603 samples, respectively, and were constructed from PROTAC‐DB 3.0, DegradeMaster markedly outperformed benchmarks such as SVM, RF, DeepPROTAC, and PROTAC‐STAN in both supervised and unsupervised settings. The model achieved AUROC scores of 0.854 and 0.882, respectively, marking improvements of up to 10.5%. It also reached an accuracy of 88.33% (correctly predicting 14 out of 16 samples) in forecasting the degradation of BRD9 by the VZ185 candidate and achieved a prediction accuracy of 77.78% for ACBI3 in degrading 17 KRAS mutants. Visualization analysis revealed that the model could intelligently identify critical structures such as the ends of the linker, the binding regions of the warhead and E3 ligand, and the distribution of attention weights, which aligns with the actual binding modes. Ablation experiments confirmed that the E(3)‐equivariant encoder is the most significant contributor to the model's performance, which decreases markedly upon its removal.

**FIGURE 3:**
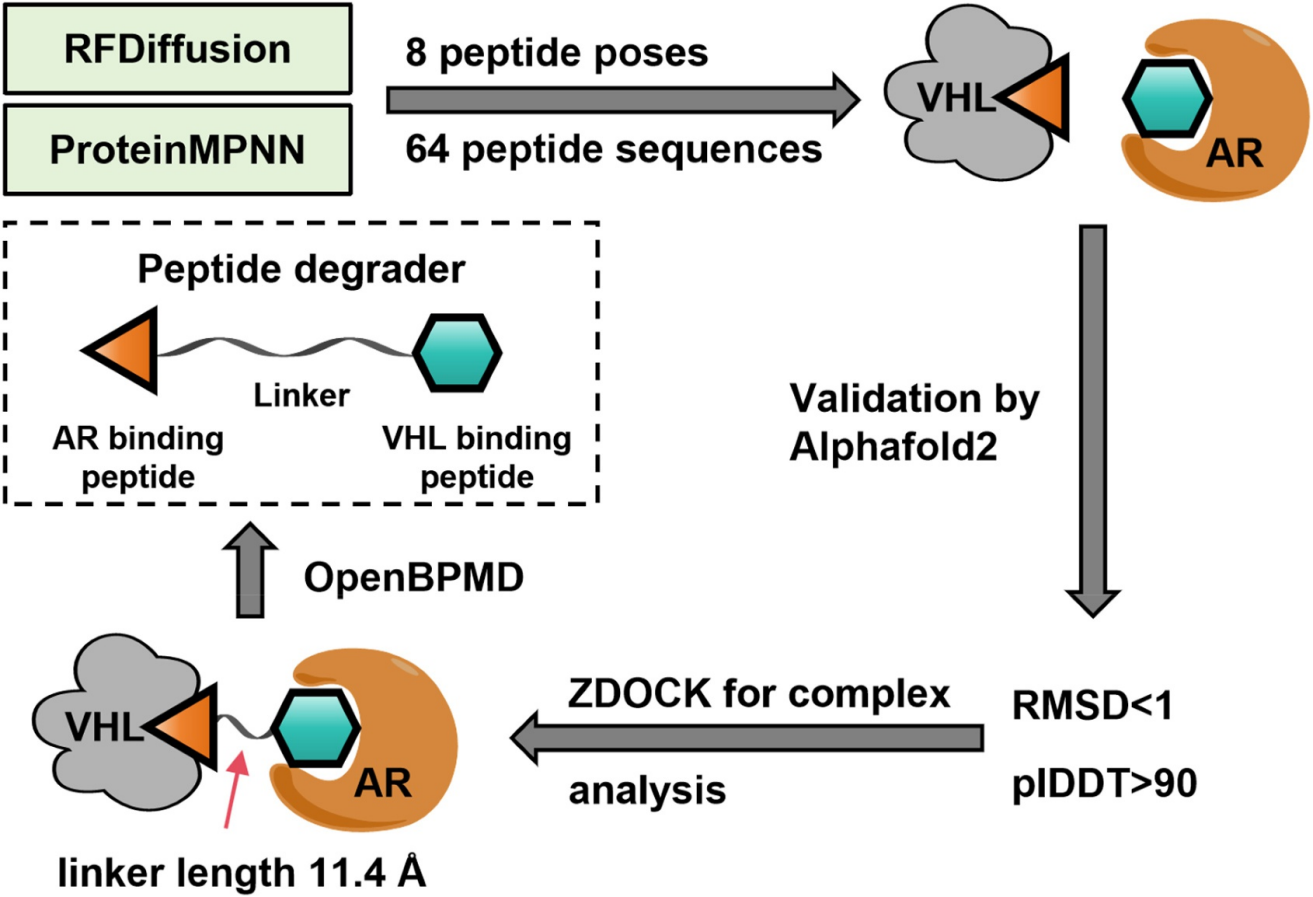
The figure illustrates the AI‐driven design workflow for peptide‐based PROTACs targeting AR. RFDiffusion is first used to generate multiple peptide conformations for both AR and VHL, followed by ProteinMPNN predicting amino acid sequence probabilities to produce candidate peptides. AlphaFold2‐multimer validates the 3D structures of the peptides bound to AR and VHL and assesses conformation accuracy retaining those with RMSD <1. ZDOCK then simulates the AR‐VHL complex to optimize linker length, and finally, MetaDynamics in OpenBPMD performs MD simulations to verify the stability of the tripartite peptide‐protein complex. This workflow establishes an efficient AI‐guided strategy for designing and optimizing peptide PROTACs for targeted AR degradation.

Ma et al. made an innovation besides small molecules employed in small‐molecule PROTAC drug design. For the development of a novel peptide‐based PROTAC therapy for androgenetic alopecia (AGA), they integrated AI algorithms with nano‐delivery technology^[44]^ (Table 2, 4). The research team employed RFdiffusion and ProteinMPNN to design binding peptides that target the AR and E3 ligase VHL. The structure was verified using AlphaFold2‐multimer. Moreover, the linker length was optimized with ZDOCK. As a result, the bifunctional molecular sequence NH_2_‐SSKLRQLLFGGSGGYGEGTSSFWL‐COOH was obtained. This molecule binds to AR and VHL with high affinities of 19.4 and 57.8 nM, respectively, and degrades the AR protein via the ubiquitin‐proteasome pathway, overcoming the limitations of traditional inhibitors that can only block functions (Figure 3). To address the issues of poor stability and low transdermal efficiency of peptide drugs, the study designed a selenium nanoparticle (Se‐AR PROTAC) modification system with a particle size of 80.7 nm and a positive surface charge of +25.3 mV to enhance cellular penetration. Hyaluronic acid was employed to prepare a soluble microneedle array (11 × 11) for non‐invasive local delivery. This delivery system can protect the drug. To be specific, the serum half‐life was extended from 0.84 to 6.8 h. Moreover, the selenium nanoparticles can scavenge reactive oxygen species (ROS). Thus, a dual mechanism of “degradation of AR and antioxidation” can be formed. In vitro experiments demonstrated that Se‐AR PROTAC effectively degrades AR with a DC_50_ of 693.1 nM in C4‐2 cells. In vivo experiments using a C57BL/6 mouse AGA model confirmed that the microneedle group was dramatically more effective than daily minoxidil when administered every two days, with the largest hair regrowth area and the thickest diameter. Additionally, the Ki‐67 proliferation marker of hair follicles increased, and AR expression markedly decreased after 15 days. As indicated by pharmacokinetics, the drug primarily remains in the skin with no residue in the blood after 8 h, and no toxicity to blood, liver, or kidney function. This study not only establishes a general AI‐driven peptide‐based PROTAC design paradigm but also expands the application of PROTAC technology from cancer to chronic disease treatment. It provides a cell‐compatible, safe, and efficient innovative solution for unmet clinical needs, such as alopecia.

### 2.2 Optimization and design of linker

Linker optimization is one of the most complex areas in PROTAC development because the linker connects the target‐binding ligand and the E3 ligase ligand, and determines the efficiency of ternary complex formation.[^36^, ^37^, ^38^] The design of linkers has been greatly improved through the application of AI, which integrates cheminformatics, molecular modeling, and ML. Traditional trial‐and‐error techniques do not always account for the non‐linear correlations between linker length, flexibility, and degradation efficiency.[^39^, ^40^] Now, AI models such as CNNs and GNNs can screen large virtual libraries of linkers and predict the conformational compatibility between ligands and their spatial orientation during ternary complex formation[^41^, ^42^, ^43^] (Table 3).

**TABLE 3:** AI for linker design and optimization in PROTACs.

Models	Molecular representations	Methods	Application scenario	Strengths	Limitations	References
DeLinker	Graph	GVAE	Scaffold hopping for molecular optimization; PROTAC degrader design	Directly integrate 3D structural information for linker generation; higher efficiency in generating high 3D similarity molecules than database methods; adapt to fragment linking, scaffold hopping and PROTAC design	Only use fragment distance and angle, exclude complete protein binding site information; denerate some molecules with poor synthesis and stability; not assign stereochemistry of generated molecules	[72]
DEVELOP	Graph	GNN/CNN	Linker design & R‐group optimization	3D pharmacophore precise constraints; support both linker design and scaffold elaboration	No direct 3D coordinate generation; rely on manual pharmacophore definition	[73]
SyntaLinker	SMILES	Transformer	Fragment linking; lead optimization; scaffold hopping	Conditional transformer controllable generation; higher recovery & novelty; no 3D conformation required	Only 2D SMILES input; no 3D spatial constraints	[74]
PROTAC‐RL	SMILES	Transformer/RL	BRD4‐targeted PROTAC design	Low‐resource generation; RL‐optimized pharmacokinetics; full generation‐screening‐assay loop	Reward function relies on empiricism; predefined attachment sites required; ternary conformation relies on simulations	[75]
DRLinker	SMILES	Transformer/RL	Fragment linking optimization; quasi‐scaffold hopping	RL‐controlled property optimization; multi‐objective simultaneous optimization; high 3D similarity & low 2D similarity	Relies on predefined scoring functions; 3D scoring computation is slow; hard to precisely control side chains	[76]
3DLinker	Graph	GNN/VAE	3D molecular linker design; 3D molecular linker design	E (3)‐equivariant 3D coordinate generation; automatic anchor prediction; joint graph & 3D structure generation	Predefined maximum node count; lower diversity & novelty; no fragment conformation perturbation	[77]
DiffLinker	Point cloud	Diffusion/GNN	Fragment‐based drug linking; pocket‐conditioned generation	E (3)‐equivariant, one‐shot multi‐fragment linking; pocket‐compatible, high synthetic accessibility	Poor for long PROTAC linkers; slightly lower chemical validity	[78]
PROTAC‐INVENT	SMILES	RL	BTK PROTAC design; BAF/BRD4 PROTAC generation	3D conformation & RL optimization; fast & accurate PROTAC docking; 2D & 3D property constraints	Relies on PTS ternary structure; limited public PROTAC structures	[79]
Link‐INVENT	SMILES	RNN/RL	Fragment linking; scaffold hopping; PROTAC design	RL multi‐objective optimization; fine‐grained linker property control; strong chemical space generalization	No 3D structural awareness; relies on scoring function quality; poor long linker generation	[80]
ShapeLinker	SMILES/Graph	Attention/RL	PROTAC linker design; ternary complex conformation matching	3D shape alignment & RL; rigid/branching precise control; 2D chemistry & 3D geometry	Conformer generation time‐consuming; poor for long complex linkers; lower synthetic accessibility	[81]
LinkerNet	Graph	Diffusion/GNN	PROTAC linker design; fragment pose co‐generation	Fragment‐linker co‐design; 3D equivariant diffusion & physics‐inspired; no fixed fragment pose constraint	Slow inference speed; no protein pocket modeling; lower novelty	[82]
FFLOM	Graph	GCN/MLP	Fragment linking/scaffold hopping; PROTAC linker design; R‐group growing & optimization	Flow & autoregressive; 100% chemical validity; high recovery & novelty	No 3D structural awareness; slightly lower synthetic accessibility; limited long‐chain generation	[83]
DiffPROTACs	Graph	Diffusion/Transformer	Generation of BRD4‐PROTAC‐VHL degradation system	O(3) equivariant; 93.86% validity; high linker diversity	Relies on high‐quality 3D data; unstable for large molecules	[84]
GRELinker	Graph	GGNN/RL/CL	A_2_A AR antagonist optimization; KRAS G12C inhibitor linker generation	Stable graph generation; CL enables complex linker generation	Relies on good scoring functions; limited for complex proteins	[87]

*Note*: Further elucidation is readily accessible within the pertinent bibliographic citations.

Abbreviation: CL, curriculum learning.

In 2020, Imrie et al. introduced DeLinker, the first GNN model for molecular generation that integrates three‐dimensional structural data explicitly to address the challenges of fragment linking and scaffold hopping in drug design^[72]^ (https://github.com/oxpig/DeLinker). Traditional computational methods depend on predefined databases to screen linkers, thus limiting the exploration of the chemical space. Although existing ML models are capable of generating new molecules, they seldom incorporate three‐dimensional structural constraints, which complicates the preservation of the original binding conformations of the fragments. DeLinker surmounts this constraint by accepting two unconnected molecular fragments, along with their relative distances and orientations, as inputs. It generates linkers through an iterative “bond‐by‐bond construction” method, ensuring that the resultant molecules not only completely retain the original fragments but also adhere to chemical valence rules. DeLinker employs an encoder‐decoder architecture, combining the GGNN and Variational Autoencoder frameworks. The encoder compresses the structural information of the fragments and reference molecules into low‐dimensional latent variables. Conversely, the decoder iteratively expands the molecular graph through a three‐step cycle of edge selection, edge labeling, and node update until the molecule is fully formed. A pivotal innovation of DeLinker is its integration of three‐dimensional geometric information into the feature vectors, which guides the generation process under structural constraints. The training of DeLinker utilized 418,000 fragment‐molecule pairs derived from the ZINC database, enabling the model to learn chemical properties such as synthetic accessibility (SA) and aromaticity without manual annotation. Comprehensive evaluations and practical applications have robustly validated DeLinker's superior performance. On an independent test set, the model demonstrated a 60% increase in the generation of molecules with high three‐dimensional similarity compared to the database baseline; this figure rose to 200% for longer linkers containing more than five atoms. Three case studies further illustrate the practical value: In the design of IMPDH inhibitors, DeLinker not only replicated all experimentally active molecules but also generated more than twice as many optimized candidates. In the case of JNK3 selective inhibitors, the model produced 182 high‐similarity skeletons, accurately reflecting the key attributes of aminopyrazole. In the context of PROTAC design, DeLinker generated over 2000 novel linkers that precisely mimicked the complex geometric configurations with pyrrole structures enhancing protein interactions through NH‐π interactions. These results indicate that three‐dimensional structural information is crucial for molecular generation, and DeLinker provides a formidable de novo design tool for structure‐based drug design.

In the following year, Imrie et al. proposed DEVELOP, a molecular generation model that integrates GNNs and CNNs, considerably improving the quality of drug design by introducing three‐dimensional pharmacophore constraints^[73]^ (https://github.com/oxpig/DEVELOP). This method addresses the issues that existing generative models face, specifically their inability to effectively use three‐dimensional structural information and lack of control over the design process. It represents molecular fragments as graph structures and converts pharmacophore features (hydrogen bond donors, acceptors, and aromatic systems) into voxel grids. These grids are then encoded by CNNs and combined with the molecular graph representation of GNNs to guide the iterative bond‐by‐bond generation process. Methodologically, DEVELOP builds upon and extends the breadth‐first generation framework of DeLinker, extracts spatial distribution information of pharmacophores through 3D convolutional layers, and forms a structural information vector by combining geometric parameters such as distance and angle to guide the decoder in generating molecules that meet pharmacophore requirements. Pharmacophore representations can be derived from known ligands or defined based on target protein structures, making the model highly applicable to drug discovery scenarios such as fragment linking, scaffold hopping, and R‐group optimization. The training data are derived from a matched molecular pair analysis of the ZINC database, constructing approximately 420,000 samples for linker design and scaffold expansion. Large‐scale tests demonstrate that the model increases the original molecule recovery rate by more than 10 times (22.4% vs. 1.9%) on the PDBbind test set, increases the proportion of molecules with high 3D similarity by 300%, and can generate a large number of alternative structures that meet design assumptions. In a case study of menin‐MLL inhibitor optimization, the model successfully reproduces two active R‐groups reported in the literature and predicts additional candidate molecules that maintain key hydrogen bond interactions. As indicated by the result of the case study, compared to one‐dimensional count information, three‐dimensional pharmacophore constraints can guide the generation of molecular space more accurately. It is therefore confirmed that the model serves as an effective tool for medicinal chemists to integrate prior knowledge into AI‐based design and is of great application value for molecular optimization in the preclinical stage.

SyntaLinker is a fragment linking tool that utilizes deep conditional Transformer neural networks to address key challenges in fragment‐based drug design^[74]^ (https://github.com/YuYaoYang2333/SyntaLinker). Yang et al. transformed the task of molecular fragment linking as a “sentence completion” problem within natural language processing. To be specific, linking rules are automatically learned based on the identified implicit grammatical patterns in SMILES strings. Thus, the limitations of traditional methods, which depend on predefined substructures or rigid transformation rules, can be overcome. The model accepts paired molecular fragments and linker constraints as input, which encompass pharmacophore information such as shortest bond distance, hydrogen bond donor or acceptor features, rotatable bonds, and ring structures. It then generates a complete molecular structure that meets these requirements using an encoder‐decoder architecture, thus achieving precise control of the linking process. Technically, the study involved extracting over 780,000 fragment‐molecule triplets from the ChEMBL database for use as training data. This data was filtered through three specific rules and SA score filtering to ensure that the generated molecules possess drug‐likeness and synthetic feasibility. The three developed model variants demonstrated more than 95% effectiveness, 90% linker novelty, and an over 80% original molecule recovery rate on the ChEMBL test set. Compared with the existing DeLinker method, SyntaLinker improved the recovery rate from 53.7% to 62.7% on the CASF validation set, and the novelty increased substantially from 51.0% to 75.4%. Furthermore, it enabled a more accurate control over the topological structure and pharmacophore features of the generated molecules. The molecules generated exhibited higher drug similarity scores and lower synthetic complexity, indicating their suitability for further medicinal chemistry optimization. The practical value of SyntaLinker was confirmed through three case studies. First, involving the IMPDH inhibitor fragment linking, the model successfully recovered active molecules and generated candidates with improved scores. In the optimization of chitinase A lead compounds, it produced 179 analogs with an RMSD of less than 2 Å, outperforming DeLinker's best results. In the scaffold hopping for JNK3 inhibitors, SyntaLinker designed 634 novel linkers covering 186 unique skeletons. An analysis using the attention mechanism confirmed that the model can accurately identify input fragments, rearrange structures, and insert linkers at the correct positions. This study highlights SyntaLinker as a data‐driven intelligent tool for fragment‐based drug design capable of efficiently constructing virtual compound libraries, optimizing lead structures, and facilitating scaffold hopping, thereby contributing to the acceleration of the early stages of drug discovery.

In 2022, Zheng et al. proposed a novel rational PROTAC design method, PROTAC‐RL (Table 2, 5), which integrates deep learning and molecular simulation to overcome challenges such as low efficiency, difficulty in exploring chemical space, and poor pharmacokinetic (PK) properties encountered in traditional PROTAC development^[75]^ (https://github.com/biomed‐AI/PROTAC‐RL). The researchers developed a generative model called Proformer, based on the Transformer architecture, to address the problem of data scarcity through a transfer learning strategy. Initially, the model was pre‐trained on 294,000 quasi‐PROTAC molecules, then fine‐tuned on a smaller, real PROTAC dataset comprising approximately 2300 examples, and further enhanced through the use of random SMILES. The model treats PROTAC design as a sentence completion task, taking target protein ligands and E3 ligase ligands as inputs and generating linkers as outputs. Subsequently, a memory‐assisted RL module was introduced, which guided the generation process using PK properties as the reward function, successfully improving the average PK score from 25.6 to 14.5. As a proof of concept, the study focused on the anti‐cancer target BRD4. Following the generation of 5000 PROTAC molecules by the model, six candidate molecules were selected for chemical synthesis. This selection was facilitated by ML activity prediction, molecular clustering, and physical simulation screening using Rosetta and MM/GBSA scores. Experimental findings revealed that three of these molecules effectively degraded the BRD4 protein in HEK293T cells. Specifically, Compound 1 exhibited activity at a concentration of 100 nM, demonstrated an anti‐proliferative IC_50_ of 116 nM against Molt4 cells, and showed low hERG cardiotoxicity at 27.4 μM. PK studies indicated that Compound 1 has a half‐life of 2.22 h in mice and an AUC of 319 ngh/mL, significantly outperforming the reference compound dBET6. The entire process from design to animal testing was completed in 49 days, achieving a hit rate of 50%. This study proves that the AI‐driven strategy can moderately expedite the discovery of PROTACs.

Tan et al. proposed the DRLinker framework, which innovatively integrates Transformer architecture with deep RL^[76]^ (https://github.com/biomed‐AI/DRlinker). This method progresses in two stages: first, it involves training the Prior model via supervised learning on the ChEMBL dataset to master SMILES grammatical patterns; subsequently, it entails fine‐tuning the Agent model using policy gradient RL, which guides the generation process with a custom scoring function. The introduction of RL eliminates the need to retrain the entire dataset for each new constraint, thereby fundamentally enhancing the model's flexibility and efficiency. DRLinker demonstrates excellent performance in multiple tasks. In terms of controlling fragment linking length, the success rates for target lengths of 5 and 10 are 94.0% and 88.0%, respectively, significantly surpassing SyntaLinker's rates of 1.6% and 1.3%. In the logP optimization task, the success rate for target values from 1 to 3 exceeds 91.9%, with an average absolute deviation of only 0.3. At the biological activity optimization level, the pChEMBL value was successfully increased by 7.5 for the JAK3 target. In the multi‐objective optimization task, the drug similarity score improved from 0.32 to 0.67, and the SA score decreased from 3.75 to 3.56. More importantly, in the quasi‐scaffold hopping experiment, the three‐dimensional similarity increased by 58.9%, while fully meeting the two‐dimensional structural novelty constraint, thereby verifying the model's capability to navigate through intellectual property limitations while maintaining the binding posture. A core advantage of DRLinker is that users only need to define the scoring function to automatically sample target molecules, thus avoiding repetitive training. The multinomial sampling strategy exhibits a stronger exploration capability than beam search, and the generated molecules display higher novelty. DRLinker provides a flexible and efficient solution for fragment‐based drug discovery (FBDD), holding significant potential for practical applications in drug design.

The 3DLinker is an E(3) equivariant variational autoencoder designed for molecular linker design. It addresses the fundamental challenge that traditional methods face in simultaneously predicting anchor atoms, generating molecular graphs, and producing precise 3D structures^[77]^ (https://github.com/YinanHuang/3DLinker). Developed by Huang et al., this model is particularly useful in the development of bifunctional molecules, such as PROTACs. Unlike existing methods that generate only 2D graphs, 3DLinker processes invariant features (node types) and equivariant features (coordinates) simultaneously through a mixed‐feature message‐passing mechanism. It incorporates a Vector Neurons network to maintain strict equivariance with respect to rotation, translation, and reflection. The model does not require preset anchor atoms and can directly generate absolute coordinates, thereby eliminating cumulative errors associated with the indirect prediction of distance matrices. Experiments demonstrate that 3DLinker significantly outperforms improved baselines on a subset of the ZINC database. Its molecular graph recovery rate reaches 93.58%, and its effectiveness is 98.67%, while the RMSD of 3D structure prediction is as low as 0.079. This represents an improvement by an order of magnitude compared to methods such as DeLinker + ConfVAE, which have RMSD values between 1.2 and 1.3. Ablation studies confirm the importance of equivariant features and dynamic coordinate update modules for enhancing geometric accuracy and graph structure quality. Furthermore, the latent representation learned by the model exhibits superior generalization capability in the QED prediction task. This work is recognized as the initial realization of end‐to‐end 3D equivariant generation of linker design. To be specific, an efficient computational framework is provided, which can balance chemical rationality with spatial accuracy for drug research and development.

DiffLinker is an innovative E(3) equivariant three‐dimensional conditional diffusion model that addresses key challenges in fragment‐based drug design by linking molecular fragments^[78]^ (https://github.com/igashov/DiffLinker). Traditional methods, which can only retrieve data from databases or perform physical simulations, are limited to linking two fragments, require predetermined connection points, and disregard protein pocket information. Through generative AI, DiffLinker overcomes the above limitations. To be specific, any number of discrete 3D molecular fragments are input. On that basis, the linker size is automatically predicted by the model. The model iteratively denoises from noise to generate missing atoms. Ultimately, a complete molecule is formed. The framework is equivariant to translation, rotation, and permutation and can integrate protein pockets as conditional constraints, offering a more flexible solution for real drug design scenarios. Technically, a two‐stage strategy is followed. Initially, the relative positions, orientations, and atom types of the fragments are analyzed by a GNN to predict the optimal linker size. Subsequently, the EGNN performs a 500‐step denoising process on initial Gaussian noise, considering the context of the fragments and pocket atoms, and simultaneously generates atom coordinates and types. By mapping discrete atom types to a continuous space and applying chemical rule constraints, DiffLinker learns molecular rationality from data without explicitly encoding valence rules. For ensuring the spatial compatibility of the generated molecules with the pockets, protein pockets are represented as atomic point clouds, and a sparse graph structure is created using a 4Å distance threshold. As indicated by the experiment results, DiffLinker significantly outperforms DeLinker and 3DLinker in the ZINC and CASF benchmark tests. It generates molecules with better QED, SA, and structural diversity. Moreover, it successfully links more than two fragments with 93% effectiveness on the GEOM dataset. Three case studies confirm its practical value: it recovers the crystal structure of Hsp90 inhibitors, generates potent IMPDH inhibitors, and provides 238 novel skeletons for JNK kinases. Although retraining is necessary for tasks involving long linkers, such as PROTACs, and chemical effectiveness could be further enhanced by incorporating valence features, the model still provides an efficient and reliable computational tool for accelerating the development of candidate drugs.

In 2023, Li et al. proposed PROTAC‐INVENT (Table 2, 6), the first PROTAC linker generation model integrating three‐dimensional structural information^[79]^ (https://github.com/jidushanbojue/Protac‐invent). In this model, REINVENT serves as the core engine. On that basis, 2D and 3D properties can be optimized through RL. Initially, the generative model outputs linker SMILES and then constructs 3D conformations through a four‐step workflow: generating initial conformations using Omega, aligning to reference molecules using ROCS, retaining the coordinates of ligands at both ends through MacroModel constraint optimization, and scoring local docking using AutoDock Vina. The reward function of RL comprehensively integrates multidimensional indicators such as docking score, pharmacophore shape similarity, linker length, number of aromatic rings, and structural alerts. This function guides the generated molecules to adapt to the binding pocket. Verified in the BTK degrader case, the model quickly converges within 50 training rounds delivering a product docking score better than that of the reference molecule, and the linker length distribution is accurately regulated according to constraint conditions. Re‐docking tests with Glide and Vina demonstrate that PROTAC‐INVENT achieves the lowest RMSD in 12 of 14 crystal structures. The time consumption is averaged as only 0.65 min. This result indicates that PROTAC‐INVENT significantly outperforms traditional methods (3–4 min). MD simulations further confirm that the binding affinities of some generated products are superior to those of the reference compounds. The model efficiently designs PROTACs. Moreover, its constrained docking workflow provides a practical solution for precise docking of large flexible ligands.

Guo et al. extended the REINVENT platform by integrating Link‐INVENT (Table 2, 7), which has renewed the drug linker design paradigm through a recurrent neural network architecture^[80]^ (https://github.com/MolecularAI/Reinvent). The model, trained on tens of thousands of molecular fragment pairs derived by reaction cutting from the ChEMBL database, learns to generate linkers that comply with SMILES grammar after receiving two molecular fragments and ultimately outputs complete molecules. Compared with traditional database search methods, its token‐level linker construction method achieves broader chemical space generalization. The training data encompasses various linking patterns, from small fragments to macrocyclic structures. On that basis, a single model is enabled to handle multiple design tasks. The core breakthrough of the system lies in its flexible scoring function and RL mechanism. The scoring function integrates multi‐parameter optimization objectives using a weighted geometric mean. Therefore, users are allowed to custom‐regulate numerous indicators (e.g., physicochemical properties, structural features, and binding energy predictions). The addition of linker‐specific parameters, such as effective length and rotatable bond ratio, allows for precise regulation of flexibility, rigidity, and branching degree. Balancing exploration and exploitation through a diversity filter, the model explores different regions in each run while generating effective linkers, ensuring that the chemical space is not overly concentrated. The study verified the practical value of the tool through three typical cases. In the CK2α kinase fragment linking task, the model generated more than 5000 unique skeletons while retaining key hydrogen bond interactions. It is noteworthy that, compared to the reference compound, some molecules achieved better docking scores. The DLK inhibitor scaffold hopping case optimized CNS penetration. Accordingly, a narrow solution space was explored, while the Cys193 hydrogen bond was maintained. The PROTAC design experiment further demonstrated the model's ability to independently regulate length, linearity, and flexibility. Link‐INVENT deeply integrates molecular docking into the learning process, substantially improving the binding affinity and structural diversity of the generated molecules. It provides an open‐source solution directly applicable to complex projects such as FBDD and PROTACs.

ShapeLinker is an innovative method for PROTAC linker design. It can achieve fragment linking through an RL‐driven autoregressive SMILES generator to address the challenge of balancing geometric constraints with chemical properties in traditional linker design^[81]^ (https://github.com/aivant/ShapeLinker). ShapeLinker employs an attention‐based point cloud alignment score to effectively incorporate geometric information into the 2D generation process. The method begins by sampling the molecular surface into a dense point cloud, then extracts global features using self‐attention and cross‐attention networks, predicts pseudo‐coordinates, and utilizes the differentiable Kabsch algorithm to achieve optimal superposition of query and reference configurations. It quantifies shape similarity using the Chamfer distance. This approach takes full advantage of GPU parallel computing, with a single alignment completed in only 230 milliseconds. The RL framework uses a composite scoring function to balance multiple objectives, assigning the highest weight to shape alignment due to its significant learning challenge and impact on activity. This optimization enhances the rigidity by optimizing the ratio of rotatable bonds and reduces branching by controlling the linker length, thus achieving a dynamic balance between geometric matching and chemical rationality. In a comprehensive evaluation involving 10 crystal structures, ShapeLinker demonstrates superior performance. The average Chamfer distance for the generated linkers is reduced to 2.19, which is better than the Link‐INVENT baseline that does not consider geometry. Moreover, it maintains 93.1% effectiveness and 99.94% chemical novelty. While ShapeLinker exhibits slightly less geometric accuracy compared to the leading 3D method, DiffLinker, it produces molecules with fewer rotatable bonds and lower branching rates, aligning more closely with the drug properties required for PROTACs. Notably, by implicitly encoding 3D constraints into the 2D generation process, the method achieves geometry awareness without requiring 3D input, thus combining computational efficiency with chemical diversity. These features establish ShapeLinker as a fast and modular rational design tool in PROTAC drug discovery, particularly suitable for the lead compound optimization stage, and offering a new technical approach for quickly exploring structure‐activity relationships.

Kao et al. proposed a deep learning framework named AIMLinker for efficiently designing and generating PROTAC molecule linkers with drug‐like properties^[68]^ (Table 2, 8). PROTACs, bifunctional molecules that degrade target proteins by recruiting E3 ubiquitin ligases, require carefully designed linkers to be effective. The study uses the CRBN‐dBET6‐BRD4 ternary complex as a model system, employs GGNN as the core architecture, and inputs two fragments: the target protein ligand and the E3 ligase ligand, along with their three‐dimensional spatial information (angles and distances between anchor points). AIMLinker generates linkers by iteratively adding atoms and forming bonds. The model includes post‐processing steps to eliminate molecules that violate chemical rules, contain unfavorable substructures, or are duplicates, and it evaluates drug‐likeness in conjunction with the “three rules.” The AIMLinker was trained on a dataset comprising 157,221 ZINC compounds and 3270 PROTAC‐DB molecules, optimizing its reconstruction ability using a Variational Autoencoder framework. Of the 2000 molecules generated, 524 candidate molecules were obtained after screening, 43% of which contain cyclic structures. These structures are more rigid and offer higher binding specificity than the linear linker of dBET6. While AIMLinker exhibits excellent performance in terms of connection correctness (100%) and uniqueness (98.3%), its effectiveness (59.8%) is lower than that of DiffLinker. However, it can accurately anchor specified positions. AutoDock4 docking verification revealed that the average RMSD for the optimal molecule, 6BOY_1268, is 0.44Å, and the binding free energy (ΔGbinding) is −62.26 kcal/mol, which is more favorable than both the crystal structure (−22.51 kcal/mol) and the re‐docked pose. A 10‐ns MD simulation demonstrated that its ΔGbinding is −45.91 ± 4.78 kcal/mol, and its ΔΔGbinding is 0.74 kcal/mol lower than that of the dBET6 crystal pose. Further, FEP calculations confirm its relative binding energy advantage of −1.25 kcal/mol. The study demonstrates that AIMLinker can effectively generate PROTAC linkers with novel structures, strong binding capabilities, and superior properties, thereby providing an efficient computational tool for structure‐based drug design.

LinkerNet refers to an innovative three‐dimensional equivariant diffusion model for TPD drugs' linker design. It can address the limitations of existing methods assuming fixed relative positions of fragments^[82]^ (https://github.com/guanjg/LinkerNet). Traditional fragment linking design depends on known binding postures. It is noteworthy that PROTACs require simultaneous binding to the target protein and the E3 ligase. Besides, the relative positions of the target protein and the E3 ligase are unknown due to protein flexibility. To address this, the study introduces a general framework for the collaborative design of fragment postures and linkers. This framework treats molecular fragments as rigid bodies, represents their global postures using rotation matrices and translation vectors, and jointly learns the atomic coordinates, types, and bonding relationships of linkers during the diffusion process. The core method comprises two key technologies: first, the design of a physics‐aware module inspired by the Newton‐Euler equations, which utilizes an equivariant GNN to predict the force and torque on each atom and calculates the translation and rotation updates for the fragment centroid, ensuring full utilization of geometric information while maintaining SE(3) equivariance. Second, the construction of a diffusion process on the manifold involves adding noise and denoising to translation and rotation using Gaussian and SO(3) equivariant distributions, respectively. This process also realizes constrained sampling through classifier guidance, which includes limiting fragment spacing and the range of anchor point selection. The model optimizes both linker structure and fragment posture at each step of the diffusion process, ultimately generating a stable conformation with low energy. Experiments conducted on the ZINC and PROTAC‐DB datasets demonstrate that the model performs excellently in both unconstrained and constrained generation scenarios. In the unconstrained setting, the molecular effectiveness reaches 83.1%, and the binding energy is markedly lower than that of baselines such as 3DLinker and DiffLinker. Furthermore, the effectiveness and recovery rate increase to 55.5% and 5.4%, respectively, in the constrained setting. The generated molecules exhibit improved drug similarity and SA. Ablation studies confirm the critical role of the physics‐inspired module in posture prediction. As the first PROTAC design model that discards the fixed fragment assumption, LinkerNet expands the available chemical space and has the potential to integrate protein context information to enhance its practical application in the future.

FFLOM (Table 2, 9) is an innovative autoregressive flow‐based model specifically developed for linker and R‐group optimization in fragment‐based drug design^[83]^ (https://github.com/Jenniferkim09/FFLOM). The model establishes a correspondence between the chemical space and a standard normal distribution through a reversible mapping mechanism. After inputting the molecular graph representation of fragments, it enables the sequential generation of atoms and bonds using multi‐layer node flow and edge flow coupling layers. During training, a masking parallel strategy is adopted to enhance efficiency, and a valence check mechanism is included during generation to ensure chemical validity. Furthermore, unreasonable structures such as continuous heteroatoms in chains and unsaturated small rings are excluded through post‐processing constraints, thus obtaining synthesizable candidate molecules. Compared with traditional methods such as VAEs and GANs, which rely on approximate inference or adversarial training, the flow‐based architecture fundamentally avoids the risks associated with mode collapse and training instability. In three benchmark tests, ZINC, CASF‐2016, and PDBbind, FFLOM demonstrates excellent performance, achieving 100% molecular effectiveness, more than 90% novelty and uniqueness, and a recovery rate exceeding 92.5%. Four practical application cases further verify its value. The fragment linking task for IMPDH inhibitors starts from two benzimidazole fragments. Over 1300 among the generated linkers, with a quantity of more than 10,000, pass property screening. Moreover, nearly 70% meet shape similarity standards, and more than 800 exhibit better docking scores than the original molecules. In the PROTAC design of BRD4, 5000 linkers are generated based on MZ1, and four molecules demonstrate further improved affinity. From the perspective of the R‐group growth of TRPM8 antagonists, almost all generated molecules maintain high conformational similarity, and nearly 200 excel in docking. In the optimization case of dabrafenib, 77 molecules with improved affinity for B‐Raf‐V600E are identified from 15,000 generated products. To be specific, 38 of these molecules also show enhanced PXR selectivity. The core advantages of FFLOM lie in its perfect reconstruction ability, guaranteed by the reversible architecture, and the flexibility and controllability brought by its autoregressive design, allowing users to customize fragment ranges and linking lengths. By limiting the dependency distance to balance training speed and reconstruction efficiency, it maintains high 3D conformational fidelity while exploring a broad chemical space. Although challenges remain in generating large molecules such as PROTACs and the SA is slightly inferior to traditional methods, the model still provides an efficient and reliable AI tool for fragment‐based drug design. In the future, by enhancing the ability to handle complex molecular distributions, it is expected to advance preclinical drug research and development towards automation and intelligence.

In 2024, Li et al. proposed a novel deep learning generative model called DiffPROTACs to address the challenge of designing linkers for PROTAC molecules^[84]^ (https://github.com/Fenglei104/DiffPROTACs). DiffPROTACs innovatively combine diffusion models with Transformer architecture. Through the O(3) equivariant graph transformer (OEGT) module, it generates reasonable linker structures based on given E3 ligands and target ligands, while maintaining the three‐dimensional spatial equivariance of molecules. The model uses Transformers to update node features and GNNs to update atomic coordinates synchronously, ensuring that molecular properties remain unchanged under rotation or reflection. To accommodate the significant differences in element composition, molecular weight, and chemical space distribution between PROTAC molecules and traditional small molecule datasets, the researchers implemented a two‐stage training strategy. Initially, they pre‐trained the model on the ZINC and GEOM datasets to master basic chemical principles, then fine‐tuned it on the specially constructed PROTAC‐DB dataset, which contains 2813 samples. Experiments demonstrated that after fine‐tuning, the model's generation effectiveness increased from 34.32% to 93.86%, and it could recover 43.75% of the test molecules. In a case study involving a BRD4 degrader, the model successfully reproduced the crystal structure with an RMSD of only 0.25–0.53Å, proving its ability to generate functional conformations. The study also constructed a virtual database containing 2.6 million PROTACs with linker lengths ranging from 5 to 28 atoms. The physicochemical property distribution of these virtual molecules closely matches that of real molecules, and Tanimoto similarity analysis confirms their excellent molecular diversity. The open‐source code, online server, and generated database of DiffPROTACs provide an efficient tool for rational design in PROTAC drug research and development, which may contribute to the acceleration of the research process in the TPD field.

Contemporary generative AI paradigms, including diffusion‐based, flow‐based, and variational autoencoder frameworks, dominate linker design pipelines. Cross‐domain foundation models further surpass limitations imposed by predefined warhead and E3 ligand fragments. Advanced biomedical foundation models directly use only POI and E3 protein sequences as inputs to autonomously generate complete bifunctional PROTAC scaffolds, encompassing the warhead, customized linker, and E3‐binding moiety. A major limitation of current open‐source foundation tools, however, is their insufficient incorporation of ADMET constraints, frequently resulting in synthetically challenging or pharmacokinetically suboptimal linker structures.[^85^, ^86^]

Zhang et al. proposed GRELinker, an innovative GNN‐based molecular fragment linking generative model that integrates RL and CL strategies for fragment linking design in drug discovery^[87]^ (https://github.com/howzh728/GRELinker). In FBDD, linking molecular fragments in two adjacent pockets is a crucial strategy for optimizing lead compounds. Traditional methods are constrained by a narrow chemical space and high computational costs, while existing deep learning methods, such as SMILES sequence generation or 3D coordinate models, lack flexibility and struggle with complex property constraints. GRELinker utilizes a GGNN architecture, representing molecules as graph structures and constructing linkers through atomic‐level iterative generation. Compared with existing SMILES models such as DRLinker, GRELinker more accurately retains input fragments and automatically predicts connection sites. GRELinker adopts a two‐stage training paradigm. Initially, it involves pre‐training the GGNN on 718,000 fragment‐molecule triplets from the ChEMBL database, where it learns molecular graph construction rules by minimizing the KL divergence of the action probability distribution. Subsequently, it fine‐tunes the model to generate molecules that meet specific properties through policy RL or CL. The model defines three actions: adding atoms, connecting atoms, and terminating graphs. It determines fragment connection positions through an anchor point prediction module. The RL framework utilizes the enhanced likelihood strategy of the REINVENT algorithm, optimizing the strategy with property scores (logP, SA, biological activity, 3D similarity, etc.) as reward signals. CL decomposes complex targets into multiple progressive sub‐targets, guiding the model to gradually learn from simple structures to complex linking patterns, which substantially improves training efficiency. In multiple benchmark tests, GRELinker markedly outperforms DRLinker. In the logP control task, its effectiveness reaches 94.56% (compared to 64.82% for DRLinker), in SA optimization, it improves by 62.3%, and it also demonstrates substantial improvements in uniqueness and novelty in tasks measuring biological activity and 3D similarity. Notably, in the case of the A2A adenosine receptor antagonist, with the docking score as the reward, the model successfully generated candidate molecules with remarkably improved affinity and automatically determined the optimal connection length as four bonds. For the complex KRAS inhibitor linker containing a bicyclic structure, CL achieved a 3D similarity of 0.92 within 550 rounds, while traditional RL only reached 0.61 with incorrect connection points. This work demonstrates that the combination of graph representation with RL and CL can effectively expand the chemical space exploration, providing an efficient and flexible molecular optimization tool for FBDD.

### 2.3 Prediction and screening of E3 ligase ligands

Two core machine learning workflows have been developed for the computational discovery and subtype classification of E3 ligase ligands: the ErG‐integrated XGBoost system and MatchMaker (Table 4). The former achieves robust classification across 17 E3 subtypes enabling rapid high‐throughput screening of ligand libraries, while MatchMaker facilitates the de novo identification of ligands for the unliganded DCAF protein. Nonetheless, sparse bioassay data for less common E3 ligase families and the suboptimal binding affinity of identified hits significantly restrict their broader application.

**TABLE 4:** AI for prediction and screening of E3 ligase ligands.

Models	Molecular representations	Methods	Application scenario	Strengths	Limitations	References
ErG	Fingerprints/descriptors	XGBoost	E3 ligase ligand selectivity prediction	High interpretability; accurate & efficient prediction	Only for known ligands; dependent on training data	[88]
MatchMaker	Fingerprints/descriptors	Concatenates	Discover novel DCAF1 ligands	Suitable for ligand‐free targets; proteome‐wide generalization	Low hit rate; weak binding affinity	[89]

*Note*: Further elucidation is readily accessible within the pertinent bibliographic citations.

Abbreviations: DCAF1, DDB1‐CUL4 associated factor 1; ErG, extended reduced graph; XGBoost, the gradient boosting model.

In 2023, Karki et al. constructed the first multi‐class ML model to predict the selectivity of E3 ligase ligands using the pharmacophore fingerprint ErG. This model aims to provide a computational screening tool for the rational design of PROTACs^[88]^ (https://github.com/Fraunhofer ITMP/E3_binder_Model). The study integrated three databases: PROTAC‐DB, PROTACpedia, and PDD. It analyzed 662 ligands spanning 17 E3 ligases and ultimately categorized them into six groups: CRBN, VHL, XIAP, CIAP1, IAP, and “Other.” ErG pharmacophore features were extracted using MOE software and the XGBoost algorithm was used to develop a classification model. This model was compared with four traditional fingerprints, including MACCS and ECFP4. The ErG model achieved the highest performance, with an accuracy of 94.0% and a Cohen's kappa coefficient of 0.881, substantially surpassing other methods. Its advantage is evident in its ability to convert 3D pharmacophore information into 2D interpretable features. The key distinguishing features were two elements: Hf_Ac_d4 (hydrophobic‐acceptor, 4‐bond distance) and Hf_D_d3 (hydrophobic‐donor, 3‐bond distance), which can cover 91% of the dataset. As indicated by pharmacophore space visualization, CRBN and VHL ligands are distinctly separated, while ligands for niche enzymes like XIAP appear blurred due to limited data. The model demonstrates that CRBN prefers ligands with succinimide ring structures, whereas VHL interactions are predominantly with hydroxyproline, aligning with established structural biology evidence. The model has been successfully applied to the screening of the Asinex degrader library, where it predicted that 66% of the compounds would likely bind to CRBN and only 2% would target VHL, thus revealing the selectivity bias of commercial libraries. Additionally, 24% of potential CRBN ligands were also identified in the Broad Institute drug repurposing library. The study's limitations include a lack of data for niche enzymes, an absence of non‐ligand verification, and experimental data support. Future plans include incorporating the AlphaFold structure database to broaden E3 ligase coverage and continuously updating the training dataset to improve generalization capabilities. The codes and data have been open‐sourced to GitHub for community use.

In the same year, Halabelian et al. successfully discovered the first small molecule ligand, CYCA‐117‐70, targeting the WD40 repeat domain of DCAF1 using the AI‐driven Drug‐Target Interaction (DTI) prediction model MatchMaker^[89]^ (https://enamine.net/compound‐collections/screening‐collection). DCAF1 serves as a key substrate recruitment subunit for CRL4 and EDVP, two types of E3 ubiquitin ligases. DCAF1 is of great significance in physiological processes including cell proliferation and DNA damage response. The functional abnormalities of DCAF1 are associated with tumorigenesis. Moreover, it is exploited by HIV/SIV viral Vpr/Vpx proteins to degrade host antiviral factors. Previously, no chemical ligands were known for this target, which hindered the application of traditional structure‐based or ligand‐based drug design methods. The research team conducted virtual screening of approximately 2.87 million compounds from the Enamine screening library against three predicted binding pockets on the DCAF1 WDR domain. The MatchMaker model processed 84 billion drug‐target pair interactions by integrating protein function annotations and binding site structural descriptors. Ultimately, proteome‐specific ranking, physicochemical property filtering, and molecular docking were employed to select 101 candidate molecules. Biophysical experimental verification demonstrated that compound CYCA‐117‐70 binds selectively to DCAF1 with an affinity of approximately 70 μM (without significantly affecting the homologous protein WDR5) and exhibits a ligand efficiency of 0.21. As indicated by the X‐ray crystallography structure (PDB: 7SSE), through hydrophobic interactions and water‐mediated hydrogen bonds, the molecule occupies the surface of the central channel of the WDR domain, partially overlapping with the binding region of the viral Vpr protein. This study confirms the applicability of the AI‐driven virtual screening method for “orphan targets.” The method can serve as a chemical tool for the functional research and drug development targeting DCAF1. The modular chemical structure and reversible binding mode of CYCA‐117‐70 make it an excellent starting point for developing inhibitors of viral‐protein interactions and DCAF1‐recruiting PROTAC degraders. Although the affinity needs optimization, this achievement demonstrates the capability of the DTI model to infer new target binding modes from existing protein data through transfer learning, offering an efficient paradigm for drug discovery targeting low‐data biomolecules and fostering open collaboration between academia and industry in precompetitive research.

In subsequent studies, the research team used DNA‐encoded library (DEL) technology to screen the WDR domain of DCAF1, identifying the initial hit Z1391232269 from 114 billion compounds through a similarity search and ML algorithms. This compound exhibited a surface plasmon resonance (SPR) Kd of 11 μM.^[90]^ After verification by differential scanning fluorimetry (DSF) and Isothermal Titration Calorimetry (ITC), this compound served as the starting point for further structural optimization. The co‐crystal structure of DCAF1 and the initial hit were analyzed. As indicated by the results, the S‐enantiomer (3d) demonstrated an affinity 27 times greater than that of the R‐enantiomer, with an SPR Kd of 490 nM. These results can reveal stereochemistry's significant impact on activity. The substitution patterns of the “northern” and “southern” aromatic rings of the molecule were systematically optimized by the team through a structure‐guided medicinal chemistry strategy. As indicated by the finding, the binding can be markedly enhanced through the introduction of meta‐halogens on the northern benzene ring and ortho/para‐halogens on the southern benzene ring. The final optimized compound, OICR‐8268 (26e), exhibits nanomolar affinity with an SPR Kd of 38 nM and an ITC Kd of 216 nM. The co‐crystal structure (PDB: 8F8E) shows that the compound is deeply embedded in the central channel of DCAF1, tightly binding to residues such as Asp1356, Arg1298, and Phe1330 through networks of hydrogen bonds, hydrophobic interactions, and halogen bonds. The cellular thermal shift assay (CETSA) confirms that OICR‐8268 substantially stabilizes the intracellular DCAF1‐WDR domain at a concentration of 10.5 μM, and its activity surpasses that of the lead compound 3d. Importantly, the binding site of this compound does not overlap with the peptide‐binding region of the viral proteins Vpx/Vpr, which avoids directly antagonizing the viral hijacking function and offers the potential for developing bifunctional molecules. As the first DCAF1 ligand with high affinity and cellular activity, OICR‐8268 not only serves as a chemical tool to explore the biological functions of DCAF1 thoroughly but also represents an ideal starting point for developing DCAF1‐based PROTAC molecules and antagonists for Merlin‐deficient tumors and HIV infections. This opens up new avenues for TPD and cancer treatment.

### 2.4 Prediction of degradation efficiency

A diverse array of computational predictors quantify PROTAC degradation potency and protein degradability, with LightGBM, MAPD, and sequence‐driven PrePROTAC serving as representative examples (Table 5). These tools leverage protein sequences, intrinsic physicochemical descriptors, and molecular fingerprints to identify potential druggable targets across the proteome. However, imbalanced data distribution and dataset bias towards CRBN‐recruiting degraders undermine predictive reliability for VHL/IAP‐dependent TPD candidates.

**TABLE 5:** AI for degradation efficiency prediction.

Models	Molecular representations	Methods	Application scenario	Strengths	Limitations	References
Nori et al.	SMILES	LightGBM	De novo PROTAC design; predict protein degradation activity	Generate large valid molecules; RL boosts activity prediction	Sparse & imbalanced training data; ignoring synthesizability	[91]
MAPD	Descriptors	NB/KNN/SVM/RF	Predict protein degradability; screen TPD drug targets	Generalize to non‐kinase proteins; predict by intrinsic protein features	Incomplete ubiquitination data; ignoring deubiquitinating enzymes	[92]
PrePROTAC	Sequence	RF/GBT	Genome‐wide degradable protein prediction; mine disease‐related potential targets	High interpretability (eSHAP); good cross‐family generalization	Limited training data; only for CRBN E3 ligase	[93]
Apprato et al.	Descriptors	RF/RT/NB/KNN/SVM	Predict AR degradation activity; screen VHL‐based PROTACs	Only needs 2D descriptors; fast early screening	Not for CRBN‐based PROTACs; dependent on data quality	[94]

*Note*: Further elucidation is readily accessible within the pertinent bibliographic citations.

Abbreviations: DCAF1, DDB1‐CUL4 associated factor 1; GBT, gradient boost tree; KNN, K‐nearest neighbors; MAPD, model‐free analysis of protein degradability; RT, random tree.

In 2022, Nori et al. proposed GraphINVENT (Table 2, 10) for the design of novel PROTAC molecules^[91]^ (https://github.com/divnori/Protac‐Design). Initially, they developed a surrogate model based on LightGBM to predict PROTAC degradation activity, which achieved a test AUC of 0.87 after training on 638 public data points. Subsequently, they adopted a graph autoregressive generative model to construct molecule atom by atom from an empty graph. For learning chemical rules, a 200‐round training was performed on 4120 molecules from PROTAC‐DB. They applied policy gradient RL for multi‐objective optimization. Through a memory‐aware RL loss function, the model was guided to generate molecules with high predicted activity, chemical effectiveness, and structural uniqueness. In the IRAK3 target case study, after 200 steps of RL fine‐tuning, the predicted activity of the generated molecules increased from 50.8% to 83.8%, the chemical effectiveness approached 100%, and all structures were novel. The generated molecules contained an average of 56 heavy atoms, equivalent to the average in the training set as well as substructures of known degraders. These substructures encompass the phthalimide skeleton of CRBN ligands. The study demonstrates that even with sparse training data, this method can learn the structural characteristics of PROTACs and effectively optimize them.

Zhang and colleagues developed an ML model named MAPD to predict the feasibility of TPD using intrinsic protein features^[92]^ (https://github.com/liulabdfci/MAPD). TPD employs the ubiquitin‐proteasome system to degrade “undruggable” proteins. However, predicting protein degradability has long been a significant challenge in the field. Based on chemical proteomics data from 91 multi‐kinase degraders, the research team analyzed the degradation of over 420 kinases and compiled a dataset that includes 42 intrinsic protein features. MAPD excelled in predicting kinase degradability through the Random Forest algorithm, achieving an AUPRC of 0.759 and an AUROC of 0.775. The model identified five key predictive features. Among these features, the ubiquitination potential serves as the most crucial indicator. To be specific, proteins featuring a higher proportion of lysine ubiquitination modifications are more likely to be degraded. Additionally, the degradability can be substantially affected by protein half‐life, phosphorylation potential, acetylation potential, and protein length. The study also discovered through structural modeling that ubiquitination sites accessible to E2 ubiquitin‐conjugating enzymes, rather than all lysine residues, markedly correlate with kinase degradability. This finding underscores the critical role of spatial accessibility in ubiquitin transfer. As a result, a structural biology foundation can be offered for degradation design. MAPD successfully predicted 77% (50 out of 65) of known PROTAC kinase targets and identified 14 potential targets not covered experimentally. Upon extending the model to the entire proteome, the team predicted 2648 degradable non‐kinase proteins. To be specific, 832 of 2648 were associated with diseases, including 206 oncogenes and 626 other disease‐related proteins. This study demonstrates that intrinsic protein features, particularly endogenous ubiquitination potential, are crucial determinants of TPD success, supplementing the traditional focus solely on ternary complex formation.

In 2023, Xie et al. developed the PrePROTAC model, which uses interpretable ML to extract sequence embedding features with the ESM protein language model. This model is combined with a Random Forest classifier to predict the degradation potential mediated by the CRBN‐E3 enzyme^[93]^ (https://github.com/XieResearchGroup/PrePROTAC). The model achieved a ROC‐AUC of 0.81 and an average precision of 0.84 on a training set comprising 445 protein kinases. It demonstrated no significant performance decline on an external test set of 50 non‐kinase domain proteins, indicating good generalization capability. The team also innovated the eSHAP analysis method, which identifies key functional residues through in silico mutagenesis. This method was validated using known complex structures, such as FAK and BRD4, and the predicted key sites showed high consistency with experimental structures. When applied to 20,504 human proteins, the PrePROTAC model identified 615 high‐confidence degradable targets among 12,475 understudied disease‐related proteins. For proteins highly expressed in Alzheimer's disease (AD), such as the Ski‐like protein (P12757), DAXX (Q9UER7), and ASCC2 (Q9H1I8), candidate molecules capable of binding both CRBN and the target protein pocket were designed. This design was facilitated by screening the PROTAC‐DB database and a fragment library through protein docking, which helped retain key interactions and reveal the binding interface. This study provides the first sequence‐based prediction framework for PROTAC target discovery, thereby expanding the potential target space for disease treatment. Current predictors, MAPD and PrePROTAC, primarily rely on isolated protein sequences or single‐layer physicochemical descriptors. In contrast, state‐of‐the‐art multimodal learning seamlessly integrates multi‐omics data (genomics, transcriptomics, proteomics, and post‐translational modification profiles) with high‐resolution structural resources, including AlphaFold‐predicted tertiary structures and experimentally determined crystallographic complexes. Multimodal learning frameworks significantly enhance the systematic in silico identification of disease‐associated proteins with predicted degradability at the proteome scale.^[94]^

Apprato et al. studied the feasibility of extracting drug design information from published PROTAC degradation data targeting the AR using computational tools^[95]^ (https://www.cs.waikato.ac.nz/ml/weka/). The study integrated degradation data from 92 PROTAC molecules, comprising 53 VHL‐recruiting and 39 CRBN‐recruiting molecules, and analyzed them using various cheminformatics methods. The objective was to provide guidance for the rational design of PROTACs. Initially, the study classified PROTACs using the Bemis‐Murcko framework, which demonstrated its inability to effectively distinguish between active and inactive molecules. This finding confirmed that PROTAC degradation activity is not merely the sum of each building module but results from synergistic effects. The degradation cliff analysis successfully identified key structural modifications that affect activity, such as the importance of ring size and the position of the amide nitrogen in the warhead. However, the analysis was limited by the applicability of existing similarity descriptors to the flexible structures of large PROTAC molecules. Furthermore, the linker analysis indicated that CRBN and VHL‐recruiting PROTACs require different optimal linker lengths, and that rigid linkers can maintain high activity when optimized for length. Ternary complex modeling corroborated the findings from the degradation cliff analysis, revealing the activity differences.

### 2.5 Prediction of ADMET properties

Multiple specialized ADMET prediction algorithms tailored specifically for TPD agents, including tree‐based ensemble models and MT‐GNN frameworks for solubility and permeability assessment (Table 6), have been developed. Leveraging experimentally annotated pharmacokinetic datasets and transfer‐learning strategies, these models facilitate efficient early‐stage lead selection. However, intrinsic data imbalances in CRBN‐associated samples and the distinctive beyond‐rule‐of‐five (bRo5) physicochemical properties of large PROTAC molecules introduce notable prediction errors for complex heterobifunctional degraders.

**TABLE 6:** AI for ADMET property prediction of TPD molecules.

Models	Molecular representations	Methods	Application scenario	Strengths	Limitations	References
Jiménez et al.	SMILES/descriptors	RF	Classify PROTAC solubility	Fast threshold judgment; based on simple descriptors	Small dataset; no modular prediction	[96]
Poongavanam et al.	SMILES/descriptors	DT/KNN/RF/SVM	Predict PROTAC permeability	High accuracy; based on simple descriptors	Imbalanced CRBN dataset; limited generalization	[97]
MT‐GNN	SMILES	MPNN/DNN learning	ADME/physicochemical property prediction for TPD molecules	Multi‐task collaborative prediction, strong generalization; fine‐tuning optimizes hard‐to‐predict molecules, improves accuracy	Higher prediction error for heterobifunctional degraders; limited prediction for some CYP properties	[98]

*Note*: Further elucidation is readily accessible within the pertinent bibliographic citations.

Abbreviations: DNN, deep neural network; DT, decision tree; MPNN, message‐passing neural network; MT‐GNN, multivariate time series forecasting with graph neural networks.

In 2022, Jiménez et al. focused on the optimization of solubility for PROTACs, which is a critical challenge in oral administration^[96]^ (https://www.cs.waikato.ac.nz/ml/weka/). The authors systematically determined the thermodynamic solubility (log S, mol/L) for 21 representative commercial PROTACs. These compounds encompass two major E3 ligase systems, CRBN and VHL, and vary in their linker and warhead structures. The findings indicated that the majority of PROTACs exhibited poor solubility, with only a few surpassing the high solubility threshold (>200 μM) set by GSK standards. Through the comparison of six computational tools, including Marvin and pkCSM, the study confirmed that existing quantitative structure‐activity relationship (QSPR) models demonstrate a poor correlation (maximum *R* ^2^ = 0.57) with PROTAC solubility, underscoring the problem of insufficient training data. The research identified lipophilicity as the primary factor influencing solubility. Both BRlogD and log k_w^IAM displayed significant negative correlations with log S (*R* ^2^ values of 0.67 and 0.61, respectively), whereas the polar parameter EPSA showed a weak correlation with Δlog k_w^IAM. Although the three‐dimensional polar surface area (3D‐PSA) was derived through conformational sampling and MD simulations, it did not perform better than the two‐dimensional TPSA (*R* ^2^ = 0.42 vs. 0.34). Based on these insights, the authors developed a three‐dimensional chemical space map incorporating BRlogD, log k_w^IAM, and TPSA, and proposed a practical decision‐making tool combined with a random tree ML model (accuracy: 86.7%). The tool suggests that a TPSA of ≥289 Å^2^ indicates high solubility; when TPSA is <289 Å^2^, a BRlogD of ≥2.58 predicts low solubility, and otherwise suggests moderate solubility. Comparative studies of three sets of PROTACs confirmed that predicting overall solubility based on the individual components (warhead, linker, E3 ligand) carries significant risks. For instance, the solubility difference between MZ1 and MZP‐54 can be attributed to the solubility of their respective warheads (JQ1 carboxylic acid vs. I‐BET726). However, in the cases of BI‐3663 and BI‐0319, VHL ligands with higher solubility paradoxically resulted in lower PROTAC solubility, likely due to changes in ionization state and the formation of intramolecular hydrogen bonds. Ultimately, the study provides an automatable solubility classification strategy for PROTACs in early drug discovery, emphasizing the need to fully consider the balance between lipophilicity and polarity. Despite its potential, this decision‐making tool requires ongoing validation and refinement within the complex bRo5 chemical space.

In 2023, Poongavanam et al. developed a binary classification model to predict the cellular permeability of PROTACs using ML techniques, with the aim of minimizing the resources required for synthesis and testing^[97]^ (https://www.knime.com). The research utilized 228 structurally diverse PROTACs, comprising 115‐VHL‐type and 113 CRBN‐type molecules. Four algorithms, decision tree, k‐nearest neighbor, random forest, and support vector machine, were employed to construct models based on 17 molecular descriptors. The PROTACs were categorized into three permeability levels: high, medium, and low. Three classification scenarios were established: high versus low, high/medium versus low, and high versus medium/low. The study revealed that the VHL‐type PROTAC model performed exceptionally well. In the high versus low classification scenario, the random forest and k‐nearest neighbor algorithms demonstrated prediction accuracies exceeding 80% with Cohen's kappa values of at least 0.57, based on a blind test set of 138 compounds. These models achieved sensitivity rates between 75% and 89% and specificity rates between 74% and 93%. In contrast, the model for CRBN‐type PROTACs showed poor performance, attributed to a significant data imbalance where samples with low permeability constituted only 7%–10% of the data. The application of the SMOTE oversampling technique did not rectify this imbalance. The most influential descriptors were found to be molecular size (molecular weight and characteristic volume) and lipophilicity (cLogD), although all 17 descriptors contributed to model performance. The impact of the linker structure on permeability was noted to be slightly more significant than that of the target protein ligands. Additionally, the study observed a progressive shift in the chemical space of PROTACs; as the cLogD increased and the TPSA decreased, the proportion of compounds classified as highly permeable rose from 40% to between 53% and 56%. Upon integrating the initial training set with the first blind test set for retraining, the performance of the VHL model on a subsequent blind test set of 52 compounds improved, with the random forest model achieving accuracies of 79%–82% and kappa values between 0.44 and 0.63. The ROC analysis indicated an excellent ability for early enrichment. The study concluded that ML‐based binary classification models are effective tools for screening in PROTAC design, although ensuring balanced training sets and strict evaluation of applicability domains are essential for reliable predictions.

In 2024, Peteani et al. conducted the first systematic evaluation of the predictive capabilities of ML‐based QSPR models for the ADMET of TPD molecules.^[98]^ The objective was to determine whether TPD molecules are within the applicability domain of existing models and to identify optimization strategies to support TPD drug design. The research team constructed four MT‐GNN global models, integrated data from 25 ADMET experimental datasets, and conducted prospective validation using time‐split validation. The findings indicated that the models' prediction accuracy for TPD molecules was comparable to that for traditional drugs, with molecular glues exhibiting the lowest prediction error (MAE: 0.11–0.28) and heterobifunctional molecules displaying slightly higher errors, yet still below 2.5‐fold. In classifying key properties such as permeability, CYP3A4 inhibition, and microsomal clearance in human and rat livers, the misclassification rate for molecular glues was below 4%, and for heterobifunctional molecules, below 15%. A transfer learning fine‐tuning strategy, where models were specifically optimized using TPD data, considerably improved the prediction performance for heterobifunctional molecules. Furthermore, models trained on a surrogate dataset of approximately 274,000 compounds, compiled from public databases, also demonstrated promising potential, with prediction outcomes highly correlated with those from internal models. This study establishes that ML‐based QSPR models are fully applicable to the TPD system, challenging the prevailing belief that TPD molecules exceed the applicability range of traditional models. This research not only creates a reliable framework for predicting TPD properties but also provides publicly available surrogate data resources, which are crucial tools for both academic and industrial TPD drug development. These findings are likely to enhance the application of ML throughout the TPD design‐synthesis‐testing‐analysis cycle, potentially accelerating the development of degraders with optimized ADMET properties and setting the groundwork for exploring the prediction of more complex properties as additional data become available in the future.

PROTACs typically exhibit large molecular sizes and properties beyond Lipinski's rule‐of‐five, making their ADMET prediction notably challenging. Unlike conventional ADMET models designed for small‐molecule drugs that conform to Lipinski's criteria, the MT‐GNN global models, RF models, and KNN classification models evaluated in this study are specifically adapted for PROTACs, effectively addressing their unique physicochemical and pharmacokinetic complexities. PROTACs generally occupy the bRo5 chemical space, characterized by high molecular weight, substantial flexibility, and significant polarity.

A key strength of these specialized models is their validated and reliable predictive accuracy for large, flexible, multi‐domain degraders, encompassing both molecular glue and heterobifunctional subtypes, whereas conventional small‐molecule models often fail or provide unreliable predictions for these structures. Critically, MT‐GNN models employ transfer learning and targeted fine‐tuning to mitigate the scarcity of publicly available experimental PROTAC data, significantly enhancing prediction accuracy for heterobifunctional degraders without requiring extensive retraining or proprietary datasets. Concurrently, RF and KNN classification models effectively capture the multifactorial determinants of PROTAC solubility and cell permeability, which are governed primarily by lipophilicity, polar surface area, and conformational dynamics rather than basic two‐dimensional descriptors. These models thus support robust binary classification and quantitative prediction, facilitating rational early‐stage PROTAC design.^[99]^

By enabling reliable risk stratification, reducing unnecessary synthesis, and guiding modular optimization of linkers, warheads, and E3 ligands, these specialized ADMET models (MT‐GNN global models, RF models, and KNN models) offer unique capabilities unmatched by traditional small‐molecule tools. Consequently, they are indispensable for accelerating the development of orally bioavailable and pharmaceutically viable degraders. While the original MT‐GNN framework utilizes only molecular graph information for ADMET regression, updated multimodal architectures incorporate hepatic transcriptomic and tissue proteomic data alongside molecular descriptors. This integration precisely predicts the complex in vivo metabolic fate, hepatic toxicity, and oral bioavailability of bRo5 TPD degraders, effectively mitigating the high prediction deviation associated with traditional ADMET tools for oversized bifunctional PROTACs.^[100]^

### 2.6 AI‐driven PROTAC to clinical transformation

While AI has significantly advanced PROTAC molecule design and optimization, clinical translation remains the definitive benchmark for validating their druggability and therapeutic value. Recently, numerous PROTAC candidates have entered late‐stage clinical development. Notably, ARV‐110 and ARV‐471 serve as landmark molecules that not only validate the clinical feasibility of PROTAC technology but also provide empirical guidance for AI‐driven rational design strategies with robust clinical efficacy and favorable drug‐like properties.[^101^, ^102^]

ARV‐110 (Bavdegalutamide), the first PROTAC to enter clinical trials, targets the AR for metastatic castration‐resistant prostate cancer.^[101]^ It induces robust AR degradation by recruiting the CRBN E3 ubiquitin ligase. In the Phase I/II ARDENT trial, ARV‐110 demonstrated effective AR degradation in patients and exhibited antitumor activity against tumors harboring AR T878A/H875Y mutations, which are resistant to standard endocrine therapies.^[103]^ These outcomes provide critical proof‐of‐concept evidence for PROTAC clinical applicability. ARV‐471 (Vepdegestrant) represents an even greater milestone, being the first PROTAC to complete Phase III trials and gain FDA approval (https://www.fda.gov/drugs/resources‐information‐approved‐drugs/fda‐approves‐vepdegestrant‐er‐positive‐her2‐negative‐esr1‐mutated‐advanced‐or‐metastatic‐breast). It targets the ER in ER+/HER2‐advanced breast cancer, efficiently degrading both wild‐type and ESR1‐mutant ER. In the Phase III VERITAC‐2 trial, ARV‐471 substantially prolonged progression‐free survival in patients with ESR1 mutations while demonstrating favorable oral bioavailability and safety. These findings confirm the clinical viability of rationally designed PROTACs, marking a transformative advancement from bench to bedside.

These clinically advanced PROTAC molecules demonstrate the translational promise of targeted protein degradation technology, verifying that PROTACs can overcome drug resistance, enable oral administration, and achieve manageable safety profiles. They provide real‐world druggable paradigms and key design principles to guide AI‐driven PROTAC research and development. Clinical data derived from ARV‐110 and ARV‐471 iteratively refine AI model optimization. For example, critical parameters such as stable ternary complex conformations, linker length and flexibility, and E3 ligase ligand preferences obtained from these molecules inform models like DeepPROTACs and PROTACable, significantly improving prediction accuracy of degradation activity. Additionally, clinical constraints derived from these studies enhance ADMET optimization, aligning AI‐driven designs more closely with clinical druggability requirements.

Based on these insights, AI can effectively reproduce and extend clinically validated design principles. For example, D16_M1P2, a PKMYT1‐targeted PROTAC designed by Insilico Medicine using the Chemistry42 platform, has advanced as a preclinical candidate.^[104]^ By integrating druggable characteristics derived from clinically validated PROTACs, this molecule demonstrated enhanced selectivity and oral bioavailability. This example underscores that AI accelerates PROTAC development from computational design through experimental validation to clinical translation, significantly shortening research cycles and improving clinical success rates.

## 3 INNOVATIVE APPLICATIONS OF AI IN MOLECULAR GLUE DEVELOPMENT

**FIGURE 4:**
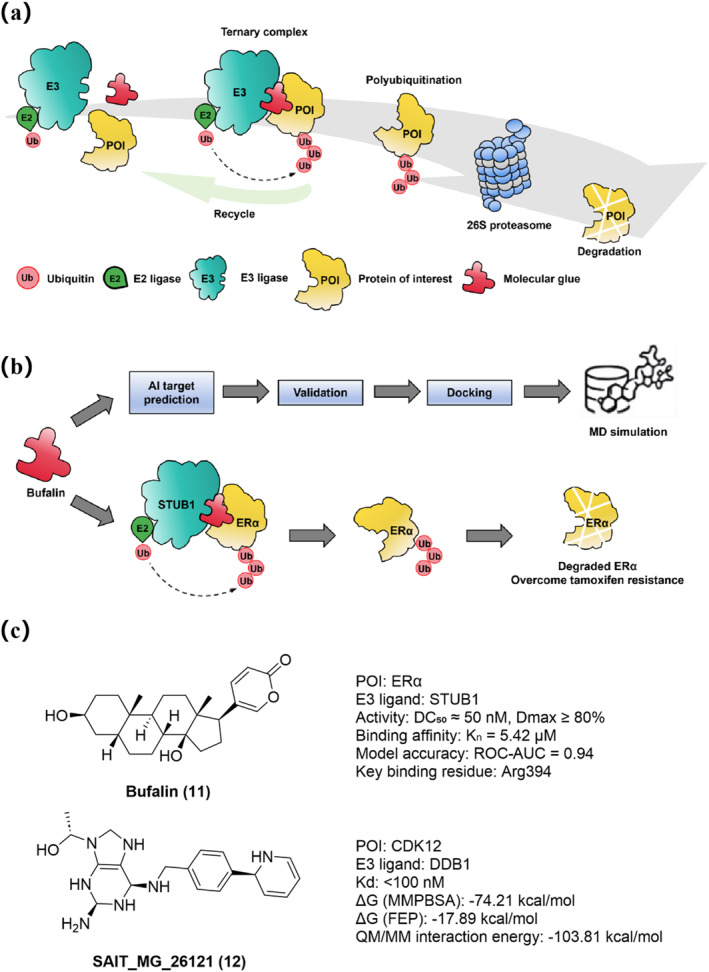
(a) The mechanism of molecular glue. (b) AI for target identification of bufalin. (c) Application examples of AI in the development of molecular glue.

Molecular glues are a class of small‐molecule compounds that regulate key intracellular biological processes by inducing or stabilizing protein‐protein interactions (PPIs).[^24^, ^105^, ^106^, ^107^] These compounds, through their unique “molecular bridge” mode of action, overcome the targeting limitations of conventional drugs that rely on the active sites of target proteins. Unlike traditional small‐molecule drugs, molecular glues do not require binding to the natural functional pockets of target proteins; rather, they facilitate the formation of stable complexes between proteins that initially have no or weak interactions. This process triggers conformational changes, degradation, or functional remodeling of the target protein[^108^, ^109^] (Figure 4a). Beyond their role in PROTAC development, AI substantially enhances molecular glue research and development by providing robust technical support in target discovery, molecular design, and activity prediction (Table 7).

**TABLE 7:** Innovative applications of AI in molecular glue development.

Name	Representation form	Method	Applications/Strengths	References
Target identification				
FMBS	Fingerprints/descriptors	NB	Identify bufalin as a molecular glue degrader of ESR1	[110]
Molecular glue design & activity prediction				
MOLDE	Molecular graph/junction tree	GMPN/TMPN	A complete calculation and design flow of molecular glue from structure prediction to affinity evaluation was established	[111]
Islam et al.	Molecular graph/junction tree	LC‐JT‐VAE	Produce chemically valid, novel, and target‐specific molecular glues capable of facilitating Aβ42 degradation	[112]
Orionis biosciences allo‐glue	Fingerprints/descriptors	NB/RF	Predict glue‐mediated recruitment of CRBN to target proteins	[113]
Kang et al.	NA	ProGlu AI/PPIExplorer	A novel TPD molecular glue degrader IPS‐06061 was found to target KRAS G12D.	[114]
Izaguirre et al.	NA	GlueMap	Provides a multi‐criteria approach to rational molecular glue design	[115]

*Note*: Further elucidation is readily accessible within the pertinent bibliographic citations.

Abbreviations: FMBS, fused multiple biological signature; GMPN, generalized most probable number; LC‐JT‐VAE, ligase‐conditioned junction tree variational autoencoder; MOLDE, molecular glue‐design‐evaluator; NA, not available; TMPN, traditional most probable number.

### 3.1 Target identification

Jiang et al. employed AI innovatively to integrate multiple prediction strategies, including FMBS and Swiss Target Prediction, together with KEGG analysis and multi‐task QSAR models. This approach identified CYP17A1, ESR1, mTOR, AR, and PRKCD as potential targets of bufalin (Figure 4c, 11) from a list of 53 candidate targets[110]. Experimental validation confirmed that bufalin directly binds to estrogen receptor α (ERα) (KD = 5.42E‐06 M) and its specific interaction was verified using surface plasmon resonance, biotin pull‐down, and thermal shift assays. MD simulations demonstrated that bufalin selectively binds to the Arg394 site of ERα and acts as a molecular glue to enhance the interaction between ERα and the E3 ubiquitin ligase STUB1, promoting the proteasomal degradation of ERα rather than inhibiting its transcriptional activity. Mechanistic studies established that bufalin induces ERα degradation via the STUB1‐mediated ubiquitin‐proteasome pathway, markedly reducing ERα protein levels, inhibiting its transcriptional activity, and downregulating the expression of target genes such as AGR2 and CCND1. In MCF‐7 and T47D cells, the anticancer effect of bufalin was dependent on ERα, and knocking down ERα attenuated its apoptosis‐inducing effect. Compared to tamoxifen, bufalin demonstrated superior ERα degradation efficacy and antitumor activity in ERα‐positive breast cancer cells. In addressing endocrine resistance, bufalin effectively induced ERα degradation in tamoxifen‐resistant LCC2 cell lines and patient‐derived organoids, inhibiting their proliferation and promoting apoptosis. In vivo experiments confirmed that bufalin substantially inhibited the growth of drug‐resistant tumors and exhibited greater efficacy than the clinical drug fulvestrant without evident liver or kidney toxicity. This study not only elucidates the novel mechanism of bufalin as a molecular glue degrading ERα but also presents a potential therapeutic strategy for overcoming tamoxifen‐resistant breast cancer, highlighting the powerful capabilities of AI‐driven natural product target discovery (Figure 4b). Beyond conventional machine learning methodologies reliant on pharmacophore fingerprints and QSAR pipelines, multimodal foundation models enable high‐throughput genome‐wide screening by combining small‐molecule structures, multi‐omics profiles, and protein sequence data. This paradigm holds particular promise for discovering molecular glues targeting traditionally undruggable proteins such as transcription factors, which historically have depended on low‐efficiency phenotypic screening approaches[107]. The integration of AI‐driven modeling further streamlines the identification of such degraders, enhancing hit rates compared to conventional screening methods.^[56]^

### 3.2 Molecular glue design & activity prediction

Geoffrey et al. introduced a novel computer‐aided rational design method known as the MOLDE, which targets significant challenges in the field of TPD.^[111]^ Generative AI and pre‐trained foundation models have transformed molecular glue discovery from a serendipitous process into a rational, structure‐driven approach. Conditioned generative diffusion and LLM‐based architectures directly generate novel molecular glue compounds tailored specifically to the POI‐E3 pocket interface. Industrial platforms, including Allo‐Glue and GlueMap, leverage pre‐trained protein foundation models to perform proteome‐wide virtual screening for potential molecular glue binders. Molecular glues, defined as small molecules that facilitate protein‐protein interactions, hold considerable potential for addressing “undruggable” targets. Historically, the discovery of these molecules largely depended on chance, with a notable absence of systematic computational design approaches. The MOLDE framework facilitates rational design through a three‐step process: initially, it identifies binding interface pockets via protein‐protein docking; subsequently, it predicts molecular glue binding modes using molecular docking; and finally, it evaluates the stability and thermodynamic properties of ternary complexes through multi‐scale MD simulations. The research team employed known molecular glue‐protein ternary complexes from the RCSB PDB database, including 6TD3, 8G46, and 7BQV for retrospective validation. Utilizing MegaDock for protein‐protein docking and AutoDock‐Vina for molecular docking, they successfully replicated the experimentally determined binding conformations. GROMACS MD simulations, combined with MMPBSA free energy calculations, confirmed that molecular glues can considerably enhance protein‐protein interactions with an increase in ΔG of 20–70 kcal/mol. To further assess the stability of complexes, FEP, TTMD, and Quantum Mechanics/Molecular Mechanics (QM/MM) calculations were employed. These methods effectively differentiated between ternary complexes with micromolar and nanomolar affinities, thus verifying the predictive capability of the framework. As a demonstration of application, the team used generative AI to design a novel molecular glue, SAIT_MG_26121, for the 6TD3 system (Figure 4c, 12). Computational evaluations indicated that this molecule interacted with key residues similarly to the reference compound RC8, exhibiting an MMPBSA binding energy of −74.21 kcal/mol, and comparable FEP and QM/MM scores to RC8. TTMD analysis confirmed that the molecule could maintain stable interactions even under elevated temperature. This study establishes the first comprehensive computational design workflow for molecular glues spanning from structure prediction to affinity evaluation. However, the authors acknowledge that the current method has been validated only on systems with known PDB structures, and its applicability to broader contexts requires further exploration. Additionally, synthesizing and testing the biological activity of AI‐designed molecules are critical areas for future research.

Islam et al. proposed a generative AI‐based framework for the design of molecular glues targeting the degradation of Aβ42, a key pathogenic protein implicated in AD.^[112]^ The characteristic pathology of AD involves the abnormal aggregation of intracellular Aβ42, which leads to synaptic dysfunction. Traditional therapies such as secretase inhibitors and immunotherapies encounter challenges including off‐target toxicity and limited penetration of the blood‐brain barrier. The study leveraged the UPS and a molecular glue strategy facilitated by E3 ubiquitin ligases (CRBN, VHL, and MDM2) to induce the formation of ternary complexes between Aβ42 and E3 enzymes, thereby achieving precise protein degradation. This approach offers a novel strategy for addressing neurodegenerative diseases. To design ligand‐specific molecular glues effectively, the research team developed a LC‐JT‐VAE. The LC‐JT‐VAE framework exemplifies the empowerment of foundation models, integrating pre‐trained protein language model weights from ProtBERT and ESM2 into a ligand‐conditioned variational autoencoder. This foundation model‐integrated approach has been successfully extended to identify molecular glues targeting oncogenic proteins such as KRAS G12D and MYC. Building on the foundational junction tree VAE, this model introduces an innovative integration of protein sequence embedding of E3 ligase binding sites, encoded by ProtBERT, and molecular torsion angle features. The model comprises a dual‐encoder architecture: one encoder captures the chemical structures, while the other captures the ligand‐binding environment. Information is integrated in the latent space through a cross‐attention mechanism. The training dataset included 65,998 compounds screened for ADMET properties and validated through docking. These compounds spanned a range of affinities, high, medium, and low, to ensure that the model learned specific ligand‐target interaction modes. The results demonstrated that the compounds generated by LC‐JT‐VAE exhibited a chemical validity exceeding 96%, with both novelty and uniqueness surpassing 80%. t‐SNE analysis indicated that the molecules explored new chemical spaces while retaining ligand specificity. Docking validation revealed that molecular glues specific to VHL, CRBN, and MDM2 had binding energies of −5.84, −5.76, and −5.78 kcal/mol, respectively, remarkably outperforming those binding to non‐target ligands. A 100‐ns MD simulation confirmed the structural stability of the ternary complexes and the persistence of hydrogen bond networks. Thus, this AI platform effectively realizes the rational design of degraders targeting previously undruggable proteins, offering a scalable and efficient tool for treating AD and other neurodegenerative diseases.

Prael III et al. conducted a large‐scale analysis using data from 161 targets and over 25,000 CRBN molecular glues produced by the Orionis Biosciences Allo‐Glue™ platform. They systematically compared 2 ML strategies, single‐task (ST) and protein chemometric (PCM), implementing four criteria for activity determination: fold change and Z‐score thresholds of 2 or 3, respectively.^[113]^ The ST model employed only compound information (ECFP4/RDKit fingerprints), whereas the PCM model combined compound information with sequence information from the target protein's G‐loop region (T‐scale, quasi‐sequence order, etc.). The study evaluated the prediction performance of the naive Bayes and random forest algorithms. The results indicated that the ST model was robust for most targets, with 89% of the models achieving a ROC AUC greater than 0.8, thus demonstrating the applicability of ligand‐based ML methods to molecular glue degrader activity prediction. The PCM model surpassed the ST model in over 80% of the targets, confirming that the integration of protein information can substantially enhance the prediction capabilities. Overall, the random forest algorithm outperformed naive Bayes, and ECFP4 fingerprints proved slightly superior to RDKit fingerprints. Among the characterizations of the G‐loop region, descriptors such as T‐scale and quasi‐sequence order, which integrate physicochemical properties and sequence information, displayed the best performance. The findings suggest that PCM modeling can effectively capture the unique protein‐protein interaction features of molecular glues, offering crucial insights for compound prioritization and target selectivity design in CRBN molecular glues and advancing TPD drug development.

Kang et al. identified a novel KRAS G12D‐degrading molecular glue using the advanced drug discovery platform ProGlu AI, which is based on IPS and the IPS chip‐based proteomics technology, PPIExplorer.^[114]^ ProGlu AI accurately predicts the PPI between the E3 ligase and KRAS G12D through MD simulation, facilitating the de novo design of molecular glue degraders. One such degrader, IPS‐06061, was synthesized and demonstrated potent anti‐tumor efficacy in xenograft animal models. These findings suggest that KRAS‐targeting molecular glues represent a promising new therapeutic approach for the treatment of pancreatic ductal adenocarcinoma (PDA), colorectal cancer (CRC), and non‐small cell lung cancer (NSCLC).

Izaguirre et al. developed a computational toolkit named GlueMap, which innovatively combines MD simulation, structural pharmacology modeling, and ML technology to establish a comprehensive workflow from virtual screening to rational design.^[115]^ GlueMap facilitates the identification of initial interaction interfaces through protein docking, generation of conformational ensembles via microsecond‐scale MD sampling, screening of metastable complexes using Markov state models, and achievement of multidimensional ranking by combining calculations of relative binding free energy with evaluations of ubiquitination site accessibility. Unlike traditional methods that depend solely on static docking scores, GlueMap integrates thermodynamic stability, structural dynamic features, and downstream degradation efficiency, thus providing a more comprehensive set of evaluation criteria for molecular glue design. In the CRBN‐GSPT1 system, GlueMap not only accurately predicted the ternary complex structure of the known molecular glue CC‐885 but also screened 11,000 compounds based on simulated encounter complexes, successfully identifying novel candidate molecules such as MG1 and MG2. Regarding the DDB1‐CDK12 case, the relative binding free energy calculations correlated well with experimental values. However, the study also uncovered a significant phenomenon: thermal stability and degradation efficiency are not strictly linearly related. Although Roscovitine and DS50 form complexes with similar stability, Roscovitine exhibits markedly stronger degradation activity because it more effectively directs the lysine residues of the target protein to the catalytic site of the E2 ligase. Based on these findings, the team developed a supervised variational autoencoder model that achieved accurate predictions of degradation efficacy by integrating structural features of the ternary complex and ubiquitination site distance parameters, with an *R* ^2^ of 0.91 on the test set. This indicates that ML can effectively discern subtle structural differences that influence degradation efficiency. The modular design of GlueMap provides both high accuracy and scalability, markedly enhancing the efficiency of thermodynamic evaluations through advanced sampling techniques and automated workflows. This tool surpasses the limitations of traditional methods in detecting weak protein interaction landscapes, translating dynamic conformational information into predictable experimental indicators, and offering mechanistic insights into molecular glue discovery. The study emphasizes the necessity of considering the complete structural dynamics of the E3 ligase complex in rational design rather than focusing solely on binary or ternary binding affinity. This approach not only facilitates the development of molecular glues for cancer‐related targets such as GSPT1 and CDK12 but also establishes a methodological foundation for expansion to membrane proteins and other ubiquitin ligase systems. This promises to enhance the application of TPD technology across a broader spectrum of therapeutic areas.

## 4 AI EXTENSION TO OTHER TPD MODALITIES

The rapid evolution of TPD technology, driven by AI, has extended beyond typical PROTACs and molecular glues to encompass a variety of forms, including LYTACs and bifunctional TPD modalities.

### 4.1 AI for LYTACs

**FIGURE 5:**
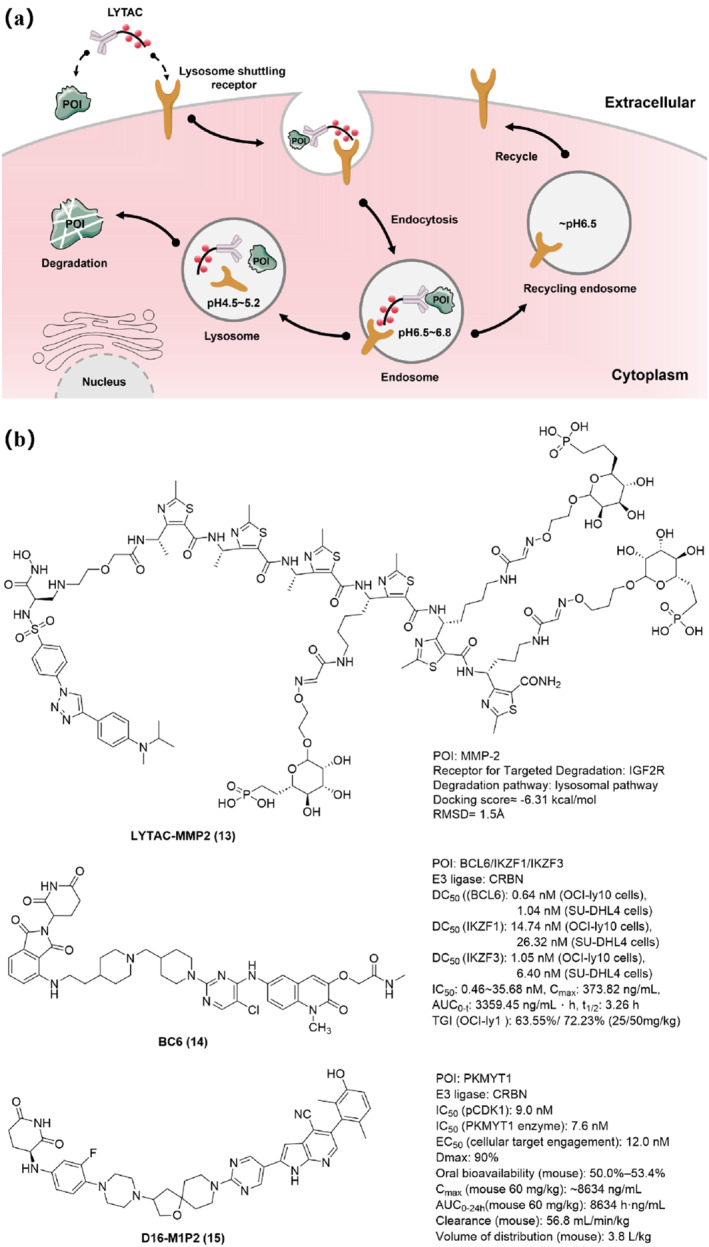
(a) The mechanism of LYTAC. (b) Application examples of AI in the development of other TPD modalities.

The LYTAC technology promotes protein degradation by binding both a cell surface receptor and a target protein utilizing the endocytic‐lysosomal pathway (Figure 5a). Cantudo et al. endeavored to develop a lysosome‐targeting chimera, specifically LYTAC‐MMP2 (Figure 5b, 13), to facilitate the targeted degradation of matrix metalloproteinase‐2 (MMP‐2)[116]. MMP‐2, which is overexpressed in various cancers, plays a significant role in tumor invasion and metastasis. The researchers chose the cation‐independent mannose‐6‐phosphate receptor (CI‐MPR) as the internalization receptor due to its mannose‐6‐phosphate (M6P)‐tagged ligand recognition capability through its MRH domains.

Using computational simulation techniques, the study investigated the interaction mechanisms between CI‐MPR and its ligands and assessed the conformational behavior of the designed LYTAC molecule, laying a theoretical groundwork for novel anticancer drug development. The research methodology included homology modeling, molecular docking, and MD simulations. Initially, the crystal structures of CI‐MPR were studied to compare the binding characteristics of four sugar recognition domains (MRH3, 5, 9, and 15) to M6P. Docking results showed that the MRH3 domain demonstrated the highest affinity for the monoester form of M6P with its “QREY” binding site sequence being notably conserved. Following this, the LYTAC‐MMP2 molecule was engineered by linking a trivalent M6Pn ligand to a selective MMP‐2 inhibitor via a γ‐peptide scaffold. A 100‐ns MD simulation of the MRH3‐M6P complex confirmed the stability of the binding, with RMSD fluctuations limited to only 1.5 Å. Unrestrained 200‐ns MD simulations were conducted separately on the CI‐MPR ligand and LYTAC‐MMP2 to observe their dynamic conformations in an aqueous environment. The findings indicated that the MRH3 domain is optimal for the interaction with LYTAC‐CI‐MPR, with the bound M6P remaining stable throughout the simulation. The LYTAC‐MMP2 molecule predominantly maintained a closed conformation in solution, though it occasionally assumed an open conformation that permits simultaneous binding of both ligand ends to CI‐MPR and MMP‐2. Notably, the charge state considerably influenced the MD behavior, with the physiologically predominant −3 state more effectively adopting the bioactive conformation compared to the −6 state. This study not only provides structural biology insights into the LYTAC technology but also confirms the dual‐targeting capability of the designed molecule.

### 4.2 AI for EndoTags

Through computational design, Huang et al. developed novel endocytosis‐inducing proteins (EndoTags) [117]. In terms of TPD and signaling regulation, the developed EndoTags can serve as a modular, genetically encodable solution. Existing technologies, such as LYTAC, rely on natural ligands and are plagued by off‐target signaling, ligand competition, and manufacturing complexities. Thus, the researchers targeted four receptors with different tissue distributions: the broadly expressed IGF2R, the liver‐specific ASGPR, the brain/muscle‐expressed TfR, and the nervous system‐enriched sortilin. They designed corresponding EndoTags to achieve efficient endocytosis using three strategies: orthosteric site binding for constitutively circulating sortilin and TfR to avoid competition with natural ligands; design of rigid fusion proteins for IGF2R to induce conformational changes in structural domains; and construction of multivalent ligands for ASGPR to promote receptor aggregation.

The experiments demonstrated that the protein‐based LYTACs (pLYTACs) formed by fusing EndoTags with the binding domain of target proteins could markedly degrade cell surface receptors (e.g., EGFR, PD‐L1), with IGF_EndoTag2 clearing more than 80% of EGFR within 48 h, outperforming traditional M6P‐LYTACs. This degradation was dependent on the expression of the corresponding receptors. In mouse tumor models, the PD‐L1 antibody atezolizumab fused with IGF_EndoTag showed appreciably enhanced tumor suppression, prolonged survival, and was well tolerated. EndoTags also cleared soluble proteins, with 70% of IgG being cleared within 48 h using Protein G‐IGF_EndoTag3. The modularity of EndoTags supports logic gating degradation: dual‐marker and gating of HER2 and EGFR via the Co‐LOCKR system, which degrades EGFR only in double‐positive cells. Additionally, EndoTag fusion proteins secreted locally by engineered cells maintain functionality and are suitable for cellular therapeutics. Most strikingly, the EndoTag fusion enhanced the signaling intensity of the synthetic Notch signaling system *ortho*‐SNIPR by nearly 100‐fold, an effect dependent on endosomes and the acidic environment. In summary, with the use of EndoTags, the limitations of natural ligands can be overcome, fabrication with an all‐protein structure can be simplified, tissue‐specific, logic‐gated, and secretable properties can be precisely degraded, and signal amplification can be combined. Therefore, EndoTags serve as a powerful tool for targeted therapeutics, synthetic biology, and drug delivery.

### 4.3 AI for bifunctional TPD modalities

Jin and colleagues developed a novel modular design strategy for PROTAC/IMiD bifunctional molecules, aiming to achieve synergistic degradation of critical lymphoma targets.^[118]^ The research team established a bifunctional molecule design‐oriented (BMDO) IMiD library comprising over 200 compounds. This library integrates a systematic workflow that includes bioinformatics analysis, validation of synergistic effects, chemical synthesis, and biological evaluation. Their analysis identified that transcription factors BCL6 and IKZF1/3 are profoundly overexpressed and positively correlated in diffuse large B‐cell lymphoma (DLBCL). The simultaneous degradation of these factors led to synergistic antitumor effects, thus providing a theoretical foundation for the design of dual‐target drugs. Employing this strategy, the researchers successfully synthesized the first BCL6/IKZF1/3 degrader, BC6 (Figure 5b, 14), and a BTK/IKZF1/3 degrader, BT6. BC6 exhibited potent degrading activity across various DLBCL cell lines, with DC_50_ values ranging from 0.08 to 0.64 nM for BCL6 and 5.74–14.74 nM for IKZF1/3. Additionally, it demonstrated favorable oral bioavailability (AUC_0‐t_ = 3359.45 ng/mL·h) and resulted in up to 72.23% inhibition of tumor growth in mouse models, appreciably surpassing the performance of single‐target degraders. Proteomic analysis further revealed that BC6 induces synergistic effects by modulating cell cycle and interferon pathways, while alterations in IRF4 expression confirmed the complementary mechanism between BCL6 and IKZF1/3 degradation. This study provides an efficient platform for the development of PROTAC/IMiD bifunctional molecules, with 9 out of 11 tested compounds (an 81.8% hit rate) demonstrating capabilities to degrade both POI and IKZF. BC6 and BT6 not only emerge as promising oral therapeutic candidates for relapsed/refractory DLBCL but also overcome the limitations of traditional drugs with insufficient activity. This work represents a remarkable advancement in integrating molecular glue and PROTAC technologies, opening new possibilities for precision oncology. In addition, Wang et al. developed a bifunctional PKMYT1‐targeted PROTAC (D16_M1P2) (Figure 5b, 15) using the AI‐driven Chemistry42 platform, a molecule that demonstrated potent antitumor activity as a monotherapy in a xenograft model through a dual mechanism of degradation and inhibition, while exhibiting high selectivity and good oral bioavailability.^[104]^

## 5 SUMMARY AND OUTLOOK

After comprehensively summarizing AI applications across various TPD modalities, we further categorize core algorithms and available datasets to clarify current technical foundations. Diverse machine learning and deep learning architectures are widely utilized in TPD computational research. The core advantages and inherent limitations of established and emerging algorithms, including RF, GNN, Transformer, and diffusion models, are presented (Table 8). Specific algorithmic characteristics determine their applicability in complex prediction tasks, linker generation, and activity forecasting, guiding informed model selection for TPD design. Datasets constitute the cornerstone of reliable TPD model training, with current datasets primarily sourced from public repositories and internal experimental collections of varying scale and annotation quality (Table 9). Uneven sample distributions and insufficient in vivo data commonly limit the generalizability of existing prediction tools.

**TABLE 8:** The core strengths of AI‐driven TPD development methods.

Methods	Core strengths	References
GCN	Excels at modeling graph‐structured data; captures node relationships and topological features; effective for molecular/protein structure learning	[56, 83]
RNN	Models sequential data with temporal dependencies; retains historical information; suitable for SMILES, protein sequence, and time‐series data	[56, 80]
NB	Fast training, low computation; robust to small datasets; strong for simple classification with independent features	[60, 92, 94, 110, 113]
Linear layer	Basic feature transformation; low complexity; easy to integrate with other modules; stable for feature projection	[61]
ReLU	Alleviates vanishing gradient; accelerates training; sparse activation; improves model nonlinearity and convergence	[61]
CNN	Extracts local spatial features; parameter sharing reduces computation; efficient for 2D/3D structural data	[62, 66, 73]
Diffusion	High precision in 3D molecule generation and conformation recovery; stepwise denoising yields extremely high valid molecule rates; supports equivariant constraints for rotation/translation invariance	[62, 78, 82, 84]
RF	Ensemble learning with high robustness; resists overfitting; interpretable; good for small‐sample bioactivity prediction	[63, 92, 93, 94, 96, 97, 113]
SVM	Excels in high‐dimensional space; good for small‐sample binary classification; robust via kernel tricks; strong generalization	[63, 92, 94, 97]
MLP	Universal approximation; simple structure; easy to train; suitable for heterogeneous feature fusion	[63, 83]
Flow	Exact likelihood calculation; stable generation; suitable for controllable molecular distribution learning	[65, 83]
RL	Optimizes target objectives directly; supports multi‐objective optimization; suitable for PROTAC property optimization	[75, 76, 79, 80, 81, 87, 91]
EGNN	Preserves 3D geometric invariance; accurate for ternary complex and spatial structure modeling	[66, 71]
Attention	Focuses on key atom/fragment linkage sites to improve accuracy; models global molecular relationships and avoids missing local structures; plug‐and‐play compatibility with most model architectures	[66, 70, 81]
GGNN	Gating mechanism controls message passing and mitigates vanishing gradients; strong ability to model long‐range molecular dependencies; ideal for generating complex molecular scaffolds	[67, 68, 87]
LLM	Understands text/molecular semantics; few‐shot learning; supports cross‐modal molecular design	[69, 70]
GNN	Naturally fits molecular graph structures and captures atom‐bond relationships; learns molecular features end‐to‐end without manual descriptors; strong generalization for diverse molecular generation tasks	[70, 73, 77, 78, 82]
Semi‐supervised learning	Uses limited labeled data + massive unlabeled data; reduces annotation cost; improves generalization	[71]
GVAE	Models molecular graphs directly and preserves topological information; smooth latent space supports interpolation and molecular optimization; enables conditional generation for fragment‐linking constraints	[72]
Transformer	Global attention models long‐range dependencies between molecular fragments; parallel computing speeds up training and inference; compatible with SMILES for syntactically consistent molecule generation	[64, 69, 74, 75, 76, 84]
VAE	Generates highly diverse molecules covering broad chemical space; controllable latent space enables precise molecular editing; stable training with no mode collapse	[77]
CL	Easy‐to‐hard training improves convergence and stability; decomposes complex tasks to lower generation difficulty; boosts generalization in low‐data scenarios	[87]
KNN	Non‐parametric and adaptive; effective for small‐sample similarity prediction; low computation cost; strong local pattern recognition	[92, 94, 97]
GBT	Models complex feature interactions; high precision in degradation prediction	[93]
RT	Fast inference speed; simple and interpretable tree structure	[94]
DT	Highly interpretable with clear rules; fast training/inference; captures nonlinear relationships; fits small structured datasets	[97]
MPNN	Directly learns from molecular graphs; captures structural/atomic features; supports multi‐task prediction; preserves molecular topology	[98]
DNN	Models complex nonlinear relationships; enables multi‐task learning; integrates diverse features; improves via transfer learning	[98]

*Note*: Further elucidation is readily accessible within the pertinent bibliographic citations.

**TABLE 9:** Compilation of representative datasets for TPD computational modeling.

Datasets	Data source and scale	Strengths	Limitations
DeepPROTAC dataset	PROTAC‐DB, total 2832 PROTAC entries; classified by DC_50_/Dmax	Clear binary classification criteria; balanced good/poor degrader grouping; contains multi‐target PROTAC data	Lacking chiral information; target distribution imbalance; no in vivo pharmacodynamic data
Ribes dataset	PROTAC‐DB + PROTAC‐pedia, 2141 experimental data	Simultaneously annotated with pDC_50_ & Dmax; open‐source; multi‐cell line information	Discrepancy of experimental conditions across different literatures; uneven E3 subtype distribution
DegradeMaster (PROTAC‐1K/PROTAC‐8K)	PROTAC‐DB3.0; 1503/8603 samples	Large dataset volume; semi‐supervised pseudo‐label expands available data; covers diverse POI/E3 combinations	Most labels are in vitro‐based; scarce in vivo degradation records; insufficient ternary crystal information
PrePROTAC dataset	445 kinase (training set)+ 50 non‐kinase proteins (external test set) + DisGeNet + pharos	Abundant protein sequence features; good cross‐family generalization; supported by eSHAP interpretability	Only limited to CRBN‐recruiting degraders; lack data for VHL/IAP series
E3 ligand dataset (Karki)	662 ligands covering 17 E3 subtypes from PROTAC‐DB 2.0, PROTACpedia and proximity degraders database	Contains mainstream E3 ligands (CRBN/VHL); pharmacophore interpretable feature	Rare E3 (XIAP/DCAF) sample shortage; few non‐active negative samples
Poongavanam permeability set	Original training set: 228 PROTAC (115 VHL/113 CRBN)	Clear VHL/CRBN grouping; unified permeability testing standard	Severe data imbalance for CRBN subset; limited molecular diversity
Jiménez solubility set	21 commercial PROTAC compounds	Uniform experimental determination of logS; verified multiple existing QSPR tools	Very small sample size; poor external generalization
MT‐GNN ADMET datasets	25 experimental ADMET sets + 274k auxiliary compounds	Covers glue and bifunctional TPD; transfer learning available; subtype differentiated error	Higher prediction error for large bifunctional PROTAC; incomplete CYP‐related data
Orionis molecular glue dataset	>25000 CRBN glue +161 targets	Massive CRBN molecular glue resources; protein sequence integrated features	Heavily biased toward CRBN; scarce VHL/MDM glue experimental data
LC‐JT‐VAE dataset	65998 filtered ADMET‐screened compounds from the Vitas and ChEMBL databases	Strict ADMET pre‐filtering; multi‐affinity graded labeling	Focused on neurodegenerative targets, narrow application scope
ZINC‐derived fragment datasets (DeLinker/DEVELOP)	∼418k/420k fragment pairs	Ultra‐large chemical space; diverse fragment skeletons; easy open access	No PROTAC‐specific biological annotation; lacking ternary interaction data
DiffPROTAC dataset	ZINC + GEOM (pre‐train), PROTAC‐DB (fine‐tune)	Separate pre‐training & fine‐tuning; high molecular validity after tuning	Rely on high‐quality 3D crystal data; unstable for oversized novel degraders

TPD is a domain undergoing groundbreaking changes with the use of AI [50]. It plays a crucial role in several aspects of TPD‐based drug discovery and development, such as designing ternary complexes for use with PROTACs, optimizing linkers, enhancing degradation efficiency, and predicting ADMET, ligand screening of E3 ubiquitin ligases, cellular permeability, oral bioavailability, and the identification and design of molecular glue targets. Thus, AI has become an invaluable tool in increasing the efficiency of research, reducing the cost of development, and shortening the period of drug discovery. It has emerged as an effective solution for targeting traditionally undruggable protein targets and addressing the numerous challenges associated with the use of TPD therapeutics. Within the context of PROTAC creation, AI technologies have successfully addressed some of the most persistent issues in the field. These include the challenges of stable ternary complex formation, complexity in linker design, variability in degradation performance, lack of drug‐likeness, and the limited number of suitable E3 ligases. AI‐assisted tools and models have not only overcome these challenges but have also improved the accuracy of PROTAC design and facilitated their translation to clinical applications.

AI is also effective in the discovery of molecular glues. It aids in the identification of targets, the design of compounds, and the prediction of activity, all of which are vital in the development of this new class of degraders. Through AI, scientists can now explore entirely novel methods of targeted degradation, markedly expanding the scope of TPD technology beyond cancer to potentially include neurological, infectious, and metabolic diseases.

### 5.1 Remaining challenges in AI‐driven TPD

Despite these positive developments, the use of AI in TPD remains in an early phase and is faced with several critical challenges:

(1) Small Size and Quality of Data

The datasets on TPD are relatively small and, in many cases, are not standardized, which may jeopardize the accuracy and generalizability of AI models.[^92^, ^119^] Models trained on small datasets can often be unreliable when applied to novel types of TPD molecules or untested disease targets.[^26^, ^120^] Most currently available TPD datasets are fragmented and lack unified formatting that integrates small‐molecule chemical structures, protein 3D conformations, and cellular or tissue multi‐omics readouts, restricting the development of robust multimodal learning systems.^[94]^

(2) Dynamic Biological Systems Static Modeling

TPD involves the sophisticated coordination of the UPS and multi‐molecular interactions.[^22^, ^33^, ^121^, ^122^, ^123^] Nevertheless, most existing AI models are based on static structural data or simplified representations that do not account for dynamic interactions, changes in conformation, and cellular context.[^98^, ^104^, ^124^] This limits the ability of the models to capture the complex biological reality of TPD in action.

(3) Black‐Box Character of Most AI Models

Many AI models, particularly those involving deep learning, are opaque. The fact that they are black boxes makes it difficult for researchers to understand the logic behind a particular prediction, thereby hindering their ability to comprehend mechanisms and restricting rational optimization. The lack of clear interpretability might make drug developers hesitant to rely on AI‐generated insights and take appropriate action.[^125^, ^126^, ^127^, ^128^] Furthermore, current biological foundation models have exacerbated black‐box limitations compared to conventional task‐specific neural networks;^[94]^ their implicit multi‐layer feature extraction fails to explicitly link atomic or substructural modifications with resultant degradation potency, substantially hindering rational lead optimization informed by experimental structural pharmacology data.

### 5.2 Future directions and opportunities

In the future, AI could be used to facilitate the research process for other TPD technologies, such as AUTACs, HyT, and ATTECs. We could use databases such as TPDdb to provide key support, predict molecular models, and achieve multi‐objective precision optimization with database updates to promote the clinical transformation of these molecules.[^57^, ^58^, ^59^] There is potential for developing game changers in TPD research by further integrating AI within the biological and chemical sciences. The next stage of development is likely to be characterized by several strategic directions:

(1) Growing and Improving Training Data

Combining multi‐omics data, including genomics, transcriptomics, proteomics, and interactomics, will enable the production of larger and more diverse datasets. Inter‐institutional and data‐sharing efforts can be utilized to construct high‐quality training sets that enhance model robustness, accuracy, and utility across a broad range of drug targets and disease models.[^129^, ^130^, ^131^] Standardized multimodal datasets will serve as foundational resources for fine‐tuning TPD‐specific foundation models, extending beyond general pre‐trained architectures. Constructing standardized multimodal TPD databases that integrate multi‐omics and structural biology data will enable robust training of multimodal neural networks and domain‐specific foundation models.

(2) Development of Dynamic and Mechanistic Models

It's not enough for the next wave of AI tools to merely guess. MD simulations, systems biology models, and real‐time interaction data can be used to develop AI systems that predict TPD process changes over time and space. Dynamic models can forecast how various components of the degradation machinery will interact and change over time, helping us understand how things break down.[^122^, ^124^, ^132^, ^133^]

(3) Moving Forward with XAI

AI drug development models must be easy to understand attracting more users. By developing XAI systems, professionals can learn how predictions are made. AI provides clear explanations that aid in molecular theories, leads to the improvement of molecules, and offers information that assists designers in making decisions early in the design process.[^134^, ^135^] Multi‐modal XAI tools quantitatively dissect the contributions of genomic mutations, protein residue alterations, and molecular scaffold modifications to degradation efficacy and ADME performance, addressing the black‐box nature of multimodal fusion models.

(4) Novel Chemical Space Exploration

Significant advances in generative AI, including GANs, VAEs, and diffusion models, powered by foundation models, unlock previously inaccessible chemical space for macrocyclic PROTACs and unconventional molecular glue scaffolds beyond traditional combinatorial chemistry, substantially accelerating chemical space exploration. Similarly, the tools discussed here can assist in constructing scaffolds with designs that were previously untried or too complex to build.^[136]^ This enables the discovery of new types of TPD molecules that are readily absorbed by organisms and metabolized in the body. With AI's help, scientists are synthesizing chemicals that structurally differ from traditional drugs, uncovering more medical applications. Additionally, AI could further facilitate the design of TPD probes (e.g., PROTAC Probe)^[52]^ by predicting probe‐POI‐E3 complex dynamics and optimizing labeling compatibility, supporting protein function research and target validation.

(5) Fostering Transdisciplinary Innovation

The future of AI in degrading specific proteins depends on effective collaboration among scientists from various fields. These systems require reliable data streams. Clinical, systems medicine, molecular biology, and chemistry data must be integrated to enhance these processes. AI technologies in structure‐based drug design have been proven effective in research and therapeutic contexts, making TPD treatment development simpler and more reliable.[^98^, ^137^] Multimodal learning integrates multi‐omics data and protein structure data to advanced AI‐driven drug design. It is beneficial for achieving precise target identification, ligand binding prediction, and lead compound optimization, thereby reducing experimental costs and improving prediction accuracy.^[138]^

(6) Customized TPD and Clinical Translation

AI also makes TPD treatment more personalized by utilizing patient‐specific data (such as changes in genomics, protein expression patterns, and disease biomarkers) in the drug development process.[^19^, ^139^] In alignment with the principles of precision medicine, this approach can be used to develop methods for degrading proteins that are tailored to each individual.[^140^, ^141^, ^142^] Thus, AI can help develop treatments that are both mechanistically and practically sound for each patient.^[143]^

## 6 CONCLUSIONS

AI is increasingly used to discover and develop TPD‐based treatments. The process of creating new drugs is enhanced by AI, which aids in predicting ADMET properties and exploring molecular glue theories. However, challenges such as simplifying model interpretability and ensuring robust validation through experimental data still require further attention. In particular, limited interpretability of deep learning models remains a notable bottleneck, restricting the translation of computationally predicted TPD candidates into clinically available drugs. Meanwhile, the synergy between computational and biological sciences is likely to enhance the safety, selectivity, and efficacy of future TPD therapies. Multi‐modal learning integrating multi‐omics and structural biology date along with foundation‐model‐driven generative AI has great potential in drug design. As these AI technologies improve and become more user‐friendly, they are expected to play a substantially larger role in advancing TPD strategies into clinical settings especially in cancer.

## AUTHOR CONTRIBUTIONS

**Shuanglin Qin**: Conceptualization; methodology; writing—original draft; writing—review and editing; supervision; funding acquisition. **Rui Peng**: Conceptualization; methodology; writing—original draft. **Guangshuai Zhang**: Conceptualization; methodology; writing—original draft. **Si Yan**: Validation. **Jin Xiao**: Validation. **Zishu Liu**: Validation. **Ziyu Tan**: Validation. **Qingqing Xie**: Validation. **Wei Luo**: Validation. **Xiaohe Xiao**: Writing—review and editing; supervision; funding acquisition.

## CONFLICT OF INTEREST STATEMENT

The authors declare no conflicts of interest.

## Data Availability

Data sharing not applicable to this article as no datasets were generated or analyzed during the current study.

## References

[1] A. Sertkaya , T. Beleche , A. Jessup , B. D. Sommers , JAMA Netw. Open 2024, 7, e2415445. 38941099 10.1001/jamanetworkopen.2024.15445 PMC11214120

[2] L. S. J. Roope , J. Control Release 2022, 345, 275. 35306118 10.1016/j.jconrel.2022.03.023 PMC8926434

[3] M. F. Stahl , A. M. Zeidan , Blood 2026, 147, 811. 41175364 10.1182/blood.2025029727

[4] D. Sun , W. Gao , H. Hu , S. Zhou , Acta Pharm. Sin. B 2022, 12, 3049. 35865092 10.1016/j.apsb.2022.02.002 PMC9293739

[5] Z.‐Z. Wang , X.‐X. Shi , G.‐Y. Huang , G.‐F. Hao , G.‐F. Yang , Trends Biochem. Sci. 2023, 48, 539. 36841635 10.1016/j.tibs.2023.01.008

[6] C. V. Dang , E. P. Reddy , K. M. Shokat , L. Soucek , Nat. Rev. Cancer 2017, 17, 502. 28643779 10.1038/nrc.2017.36 PMC5945194

[7] P. A. Kiberstis , Science 2016, 353, 1510.

[8] N. Yang , B. Kong , Z. Zhu , F. Huang , L. Zhang , T. Lu , Y. Chen , Y. Zhang , Y. Jiang , Mol. Divers. 2024, 28, 309. 36790583 10.1007/s11030-023-10606-w PMC9930057

[9] C. Zhang , Y. Liu , G. Li , Z. Yang , C. Han , X. Sun , C. Sheng , K. Ding , Y. Rao , Sci. Bull. 2024, 69, 1776. 10.1016/j.scib.2024.03.056 38614856

[10] G. Zhang , S. Yan , Y. Liu , Z. Du , Q. Min , S. Qin , Bioanalysis 2025, 17, 261. 39895280 10.1080/17576180.2025.2459528 PMC11864318

[11] Y. Lu , Y. Yang , G. Zhu , H. Zeng , Y. Fan , F. Guo , D. Xu , B. Wang , D. Chen , G. Ge , Int. J. Biol. Sci. 2023, 19, 3360. 37496997 10.7150/ijbs.83026 PMC10367563

[12] G. Zhang , J. Zhang , Y. Gao , Y. Li , Y. Li , Expert Opin. Drug Dis. 2022, 17, 55. 10.1080/17460441.2021.1969359 34455870

[13] J.‐j. Zhuang , Q. Liu , D.‐l. Wu , L. Tie , Acta Pharmacol. Sin. 2022, 43, 2474. 35132191 10.1038/s41401-021-00852-9 PMC9525275

[14] J. H. Bushweller , Nat. Rev. Cancer 2019, 19, 611. 31511663 10.1038/s41568-019-0196-7 PMC8820243

[15] M. J. Henley , A. N. Koehler , Nat. Rev. Drug Discov. 2021, 20, 669. 34006959 10.1038/s41573-021-00199-0

[16] Y. Li , J. Song , P. Zhou , J. Zhou , S. Xie , J. Med. Chem. 2022, 65, 10183. 35881047 10.1021/acs.jmedchem.2c00691

[17] R. Santos , O. Ursu , A. Gaulton , A. P. Bento , R. S. Donadi , C. G. Bologa , A. Karlsson , B. Al‐Lazikani , A. Hersey , T. I. Oprea , J. P. Overington , Nat. Rev. Drug Discov. 2016, 16, 19. 27910877 10.1038/nrd.2016.230 PMC6314433

[18] K. Vasan , D. M. Gysi , A.‐L. Barabási , iScience 2023, 26, 108361. 38146432 10.1016/j.isci.2023.108361 PMC10749231

[19] G. Zhong , X. Chang , W. Xie , X. Zhou , Signal. Transduction Targeted Ther. 2024, 9, 308. 10.1038/s41392-024-02004-x PMC11539257 39500878

[20] J. M. Tsai , R. P. Nowak , B. L. Ebert , E. S. Fischer , Nat. Rev. Mol. Cell Bio. 2024, 25, 740. 38684868 10.1038/s41580-024-00729-9

[21] J. Kim , I. Byun , D. Y. Kim , H. Joh , H. J. Kim , M. J. Lee , Chem. Soc. Rev. 2024, 53, 3253. 38369971 10.1039/d3cs00344b

[22] L. Zhao , J. Zhao , K. Zhong , A. Tong , D. Jia , Signal. Transduction Targeted Ther. 2022, 7, 113. 10.1038/s41392-022-00966-4 PMC8977435 35379777

[23] K. T. G. Samarasinghe , C. M. Crews , Cell Chem. Biol. 2021, 28, 934. 34004187 10.1016/j.chembiol.2021.04.011 PMC8286327

[24] X. Tan , Z. Huang , H. Pei , Z. Jia , J. Zheng , iScience 2024, 27, 110712. 39297173 10.1016/j.isci.2024.110712 PMC11409024

[25] M. Békés , D. R. Langley , C. M. Crews , Nat. Rev. Drug Discov. 2022, 21, 181. 35042991 10.1038/s41573-021-00371-6 PMC8765495

[26] K. Farley , S. Bhattacharya , J. Cleland , P. Chandran , J. Wu , Nat. Rev. Drug Discov. 2025, 24, 164. 39604677 10.1038/d41573-024-00187-0

[27] M. Hinterndorfer , V. A. Spiteri , A. Ciulli , G. E. Winter , Nat. Rev. Cancer 2025, 25, 493. 40281114 10.1038/s41568-025-00817-8

[28] S. Qin , X. Xiao , Future Med. Chem. 2025, 17, 987. 40314207 10.1080/17568919.2025.2498875 PMC12091913

[29] D. A. Nalawansha , C. M. Crews , Cell Chem. Biol. 2020, 27, 998. 32795419 10.1016/j.chembiol.2020.07.020 PMC9424844

[30] X. Huang , F. Wu , J. Ye , L. Wang , X. Wang , X. Li , G. He , Acta Pharm. Sin. B 2024, 14, 2402. 38828146 10.1016/j.apsb.2024.01.010 PMC11143490

[31] X. Xie , T. Yu , X. Li , N. Zhang , L. J. Foster , C. Peng , W. Huang , G. He , Signal. Transduction Targeted Ther. 2023, 8, 335. 10.1038/s41392-023-01589-z PMC10480221 37669923

[32] A. Hanzl , G. E. Winter , Curr. Opin. Chem. Biol. 2020, 56, 35. 31901786 10.1016/j.cbpa.2019.11.012 PMC7615046

[33] J. Song , M. Hu , J. Zhou , S. Xie , T. Li , Y. Li , Eur. J. Med. Chem. 2023, 261, 115839. 37778240 10.1016/j.ejmech.2023.115839

[34] Y. Fang , S. Wang , S. Han , Y. Zhao , C. Yu , H. Liu , N. Li , Trends Pharmacol. Sci. 2023, 44, 303. 37059054 10.1016/j.tips.2023.03.003

[35] F. Shen , L. M. K. Dassama , Chem. Sci. 2023, 14, 8433. 37592990 10.1039/d3sc02361c PMC10430753

[36] M. Duran‐Frigola , M. Cigler , G. E. Winter , J. Am. Chem. Soc. 2023, 145, 2711. 36706315 10.1021/jacs.2c11098 PMC9912273

[37] S. Tan , Z. Chen , R. Lu , H. Liu , X. Yao , WIREs Comput. Mol. Sci. 2025, 15, e70013.

[38] M. S. Jamal Danishuddin , K.‐S. Song , K.‐W. Lee , J.‐J. Kim , Y.‐M. Park , Y. M. Park , Pharmaceuticals 2023, 16, 1649. 38139776 10.3390/ph16121649 PMC10747325

[39] Y. Gharbi , R. Mercado , Digit. Discov. 2024, 3, 2158.

[40] J. Ge , C.‐Y. Hsieh , M. Fang , H. Sun , T. Hou , Trends Pharmacol. Sci. 2024, 45, 1162. 39567313 10.1016/j.tips.2024.10.006

[41] S. Shaik , P. Kumar Reddy Gayam , M. Chaudhary , G. Singh , A. Pai , Bioorg. Chem. 2024, 153, 107868. 39374557 10.1016/j.bioorg.2024.107868

[42] A. Abbas , F. Ye , Int. J. Bio. Macromol. 2024, 277, 134293. 39084437 10.1016/j.ijbiomac.2024.134293

[43] W. Lv , X. Jia , B. Tang , C. Ma , X. Fan , X. Jin , Z. Niu , X. Han , Eur. J. Med. Chem. 2025, 289, 117432. 40015161 10.1016/j.ejmech.2025.117432

[44] B. Ma , D. Liu , Z. Wang , D. Zhang , Y. Jian , K. Zhang , T. Zhou , Y. Gao , Y. Fan , J. Ma , Y. Gao , Y. Chen , S. Chen , J. Liu , X. Li , L. Li , J. Med. Chem. 2024, 67, 10336. 38836467 10.1021/acs.jmedchem.4c00828

[45] K. M. Sakamoto , K. B. Kim , A. Kumagai , F. Mercurio , C. M. Crews , R. J. Deshaies , PANS (Pest. Artic. News Summ.) 2001, 98, 8554. 10.1073/pnas.141230798 PMC37474 11438690

[46] S.‐M. Qi , J. Dong , Z.‐Y. Xu , X.‐D. Cheng , W.‐D. Zhang , J.‐J. Qin , Front. Pharmacol. 2021, 12, 692574. 34025443 10.3389/fphar.2021.692574 PMC8138175

[47] C. Arnold , Nat. Med. 2024, 30, 3030. 39379566 10.1038/d41591-024-00072-8

[48] C. Wang , Y. Zhang , W. Chen , Y. Wu , D. Xing , Mol. Cancer 2024, 23, 110. 38773495 10.1186/s12943-024-02024-9 PMC11107062

[49] X. Wang , Z.‐L. Qin , N. Li , M.‐Q. Jia , Q.‐G. Liu , Y.‐R. Bai , J. Song , S. Yuan , S.‐Y. Zhang , Eur. J. Med. Chem. 2024, 267, 116166. 38281455 10.1016/j.ejmech.2024.116166

[50] D. Chirnomas , K. R. Hornberger , C. M. Crews , Nat. Rev. Clin. Oncol. 2023, 20, 265. 36781982 10.1038/s41571-023-00736-3 PMC11698446

[51] M. F. Mabanglo , B. Wilson , M. Noureldin , S. W. Kimani , A. Mamai , C. Krausser , H. González‐Álvarez , S. Srivastava , M. Mohammed , L. Hoffer , M. Chan , J. Avrumutsoae , A. S. M. Li , T. Hajian , S. Tucker , S. Green , M. Szewczyk , D. Barsyte‐Lovejoy , V. Santhakumar , S. Ackloo , P. Loppnau , Y. Li , A. Seitova , T. Kiyota , J. G. Wang , G. G. Privé , D. A. Kuntz , B. Patel , V. Rathod , A. Vala , B. Rout , A. Aman , G. Poda , D. Uehling , J. Ramnauth , L. Halabelian , R. Marcellus , R. Al‐awar , M. Vedadi , Nat. Commun. 2024, 15, 10165. 39580491 10.1038/s41467-024-54500-x PMC11585590

[52] S. Yan , G. Zhang , W. Luo , M. Xu , R. Peng , Z. Du , Y. Liu , Z. Bai , X. Xiao , S. Qin , Eur. J. Med. Chem. 2024, 276, 116725. 39083982 10.1016/j.ejmech.2024.116725

[53] F. Xue , M. Zhang , S. Li , X. Gao , J. A. Wohlschlegel , W. Huang , Y. Yang , W. Deng , Nat. Commun. 2025, 16, 5514. 40593782 10.1038/s41467-025-61272-5 PMC12216337

[54] M. P. Schwalm , A. Krämer , A. Dölle , J. Weckesser , X. Yu , J. Jin , K. Saxena , S. Knapp , Cell Chem. Biol. 2023, 30, 753. 37354907 10.1016/j.chembiol.2023.06.002

[55] M. S. Gadd , A. Testa , X. Lucas , K.‐H. Chan , W. Chen , D. J. Lamont , M. Zengerle , A. Ciulli , Nat. Chem. Biol. 2017, 13, 514. 28288108 10.1038/nchembio.2329 PMC5392356

[56] F. Li , Q. Hu , X. Zhang , R. Sun , Z. Liu , S. Wu , S. Tian , X. Ma , Z. Dai , X. Yang , S. Gao , F. Bai , Nat. Commun. 2022, 13, 7133. 36414666 10.1038/s41467-022-34807-3 PMC9681730

[57] G. Weng , X. Cai , D. Cao , H. Du , C. Shen , Y. Deng , Q. He , B. Yang , D. Li , T. Hou , Nucleic Acids Res. 2023, 51, D1367. 36300631 10.1093/nar/gkac946 PMC9825472

[58] J. Ge , S. Li , G. Weng , H. Wang , M. Fang , H. Sun , Y. Deng , C.‐Y. Hsieh , D. Li , T. Hou , Nucleic Acids Res. 2025, 53, D1510. 39225044 10.1093/nar/gkae768 PMC11701630

[59] G. Weng , C. Shen , D. Cao , J. Gao , X. Dong , Q. He , B. Yang , D. Li , J. Wu , T. Hou , Nucleic Acids Res. 2021, 49, D1381. 33010159 10.1093/nar/gkaa807 PMC7778940

[60] A. Rao , T. M. Tunjic , M. Brunsteiner , M. Müller , H. Fooladi , C. Gasbarri , N. Weber , Artif. Intel. Life Sci. 2023, 3, 10072.

[61] S. Ribes , E. Nittinger , C. Tyrchan , R. Mercado , Artif. Intel. Life Sci. 2024, 6, 100104.

[62] B. G. A S , D. Agrawal , N. M. Kulkarni , R. Vetrivel , K. Gurram , ACS Omega 2024, 9, 12611. 38524483 10.1021/acsomega.3c07318 PMC10955709

[63] A. S. Abouzied , B. Alshammari , H. Kari , B. Huwaimel , S. Alqarni , S. E. Kassab , Mol. Divers. 2024, 29, 2995. 39425859 10.1007/s11030-024-11011-7

[64] H. Mslati , F. Gentile , M. Pandey , F. Ban , A. Cherkasov , J. Chem. Inf. Model. 2024, 64, 3034. 38504115 10.1021/acs.jcim.3c01878

[65] B. Qiang , W. Shi , Y. Song , M. Wu , ArXiv 2024, abs/2405.06654.

[66] L. Cai , G. Yue , Y. Chen , L. Wang , X. Yao , Q. Zou , X. Fu , D. Cao , Brief. Bioinform. 2025, 26, bbae654. 10.1093/bib/bbae654 PMC11713031 39783892

[67] C.‐L. Chou , C.‐T. Lin , C.‐T. Kao , C.‐C. Lin , ACS Omega 2024, 9, 38371. 39310161 10.1021/acsomega.3c10183 PMC11411543

[68] C.‐T. Kao , C.‐T. Lin , C.‐L. Chou , C.‐C. Lin , J. Chem. Inf. Model. 2023, 63, 2918. 37150933 10.1021/acs.jcim.2c01287 PMC10207268

[69] J. Shao , Q. Gong , Z. Yin , Y. Chen , Y. Hao , L. Zhang , L. Jiang , M. Yao , J. Li , F. Wang , L. Wang , ArXiv 2024, abs/2412.09661.

[70] Z. Chen , C. Gu , S. Tan , X. Wang , Y. Li , M. He , R. Lu , S. Sun , C. Y. Hsieh , X. Yao , H. Liu , P. A. Heng , Adv. Sci. 2025, 12, e08138. 10.1002/advs.202508138 PMC12713099 41026144

[71] J. Liu , M. J. Roy , L. Isbel , F. Li , Bioinformatics 2025, 41, i342. 40662822 10.1093/bioinformatics/btaf191 PMC12261415

[72] F. Imrie , A. R. Bradley , M. van der Schaar , C. M. Deane , J. Chem. Inf. Model. 2020, 60, 1983. 32195587 10.1021/acs.jcim.9b01120 PMC7189367

[73] F. Imrie , T. E. Hadfield , A. R. Bradley , C. M. Deane , Chem. Sci. 2021, 12, 14577. 34881010 10.1039/d1sc02436a PMC8580048

[74] Y. Y. Yang , S. J. Zheng , S. M. Su , C. Zhao , J. Xu , H. M. Chen , Chem. Sci. 2020, 11, 8312. 34123096 10.1039/d0sc03126g PMC8163338

[75] S. Zheng , Y. Tan , Z. Wang , C. Li , Z. Zhang , X. Sang , H. Chen , Y. Yang , Nat. Mach. Intell. 2022, 4, 739.

[76] Y. Tan , L. Dai , W. Huang , Y. Guo , S. Zheng , J. Lei , H. Chen , Y. Yang , J. Chem. Inf. Model. 2022, 62, 5907. 36404642 10.1021/acs.jcim.2c00982

[77] Y. Huang , X. Peng , J. Ma , M. Zhang , Arxiv ‐ CS ‐ Artif. Intell. 2022, arxiv‐2205.07309.

[78] I. Igashov , H. Stärk , C. Vignac , A. Schneuing , V. G. Satorras , P. Frossard , M. Welling , M. Bronstein , B. Correia , Nat. Mach. Intell. 2024, 6, 417.

[79] B. Li , T. Ran , H. Chen , Briefings Bioinf. 2023, 24, bbad323.

[80] J. Guo , F. Knuth , C. Margreitter , J. P. Janet , K. Papadopoulos , O. Engkvist , A. Patronov , Digit. Discov. 2023, 2, 392.

[81] R. M. Neeser , M. Akdel , D. Kovtun , L. Naef , ArXiv 2023, abs/2306.08166.

[82] M. Ning , Y. Zhou , G. Chen , X. Mei , Adv. Neural Inf. Process. Syst. 2023, 12223.

[83] J. Jin , D. Wang , G. Shi , J. Bao , J. Wang , H. Zhang , P. Pan , D. Li , X. Yao , H. Liu , T. Hou , Y. Kang , J. Med. Chem. 2023, 66, 10808. 37471134 10.1021/acs.jmedchem.3c01009

[84] F. Li , Q. Hu , Y. Zhou , H. Yang , F. Bai , Briefings Bioinf. 2024, 25, bbae358. 10.1093/bib/bbae358 PMC11299039 39101502

[85] W. GaoConnor , W. Coley , J. Chem. Inf. Model. 2020, 60, 5714. 32250616 10.1021/acs.jcim.0c00174

[86] R. L. van den Broek , S. Patel , G. J. van Westen , W. Jespers , W. Sherman , J. Chem. Inf. Model. 2025, 65, 9383. 40893044 10.1021/acs.jcim.5c01203 PMC12458704

[87] H. Zhang , J. Huang , J. Xie , W. Huang , Y. Yang , M. Xu , J. Lei , H. Chen , J. Chem. Inf. Model. 2024, 64, 666. 38241022 10.1021/acs.jcim.3c01700

[88] R. Karki , Y. Gadiya , P. Gribbon , A. Zaliani , ACS Omega 2023, 8, 30177. 37636935 10.1021/acsomega.3c02803 PMC10448689

[89] S. W. Kimani , J. Owen , S. R. Green , F. Li , Y. Li , A. Dong , P. J. Brown , S. Ackloo , D. Kuter , C. Yang , M. MacAskill , S. S. MacKinnon , C. H. Arrowsmith , M. Schapira , V. Shahani , L. Halabelian , J. Chem. Inf. Model. 2023, 63, 4070. 37350740 10.1021/acs.jcim.3c00082 PMC10337664

[90] A. S. M. Li , S. Kimani , B. Wilson , M. Noureldin , H. González‐Álvarez , A. Mamai , L. Hoffer , J. P. Guilinger , Y. Zhang , M. von Rechenberg , J. S. Disch , C. J. Mulhern , B. L. Slakman , J. W. Cuozzo , A. Dong , G. Poda , M. Mohammed , P. Saraon , M. Mittal , P. Modh , V. Rathod , B. Patel , S. Ackloo , V. Santhakumar , M. M. Szewczyk , D. Barsyte‐Lovejoy , C. H. Arrowsmith , R. Marcellus , M.‐A. Guié , A. D. Keefe , P. J. Brown , L. Halabelian , R. Al‐awar , M. Vedadi , J. Med. Chem. 2023, 66, 5041. 36948210 10.1021/acs.jmedchem.2c02132 PMC10108359

[91] D. Nori , C. W. Coley , R. Mercado , ArXiv 2022, abs/2211.02660.

[92] W. Zhang , S. S. Roy Burman , J. Chen , K. A. Donovan , Y. Cao , C. Shu , B. Zhang , Z. Zeng , S. Gu , Y. Zhang , D. Li , E. S. Fischer , C. Tokheim , X. Shirley Liu , Genom. Proteom. Bioinf. 2022, 20, 882. 10.1016/j.gpb.2022.11.008 PMC10025769 36494034

[93] A. R. Panchenko , L. Xie , L. Xie , PLoS Comput. Biol. 2023, 19, e1010974. 37590332 10.1371/journal.pcbi.1010974 PMC10464998

[94] H. Cui , A. Tejada‐Lapuerta , M. Brbić , J. Saez‐Rodriguez , S. Cristea , H. Goodarzi , M. Lotfollahi , F. J. Theis , B. Wang , Nature 2025, 640, 623. 40240854 10.1038/s41586-025-08710-y

[95] G. Apprato , G. D’Agostini , P. Rossetti , G. Ermondi , G. Caron , Molecules 2023, 28, 1206. 36770875 10.3390/molecules28031206 PMC9919651

[96] D. García Jiménez , M. Rossi Sebastiano , M. Vallaro , V. Mileo , D. Pizzirani , E. Moretti , G. Ermondi , G. Caron , J. Med. Chem. 2022, 65, 12639. 35469399 10.1021/acs.jmedchem.2c00201 PMC9574862

[97] V. Poongavanam , F. Kölling , A. Giese , A. H. Göller , L. Lehmann , D. Meibom , J. Kihlberg , ACS Omega 2023, 8, 5901. 36816707 10.1021/acsomega.2c07717 PMC9933238

[98] G. Peteani , M. T. D. Huynh , G. Gerebtzoff , R. Rodríguez‐Pérez , Nat. Commun. 2024, 15, 5764. 38982061 10.1038/s41467-024-49979-3 PMC11233499

[99] F. Montanari , L. Kuhnke , A. Ter Laak , D.‐A. Clevert , Molecules 2020, 25, 44. 10.3390/molecules25010044 PMC6982787 31877719

[100] E. N. Feinberg , E. Joshi , V. S. Pande , A. C. Cheng , J. Med. Chem. 2020, 63, 8835. 32286824 10.1021/acs.jmedchem.9b02187

[101] L. B. Snyder , T. K. Neklesa , R. R. Willard , D. A. Gordon , J. Pizzano , N. Vitale , K. Robling , M. A. Dorso , W. Moghrabi , S. Landrette , R. Gedrich , S. H. Lee , I. C. A. Taylor , J. G. Houston , Mol. Cancer Ther. 2025, 24, 511. 39670468 10.1158/1535-7163.MCT-23-0655 PMC11962395

[102] E. P. Hamilton , C. Ma , M. De Laurentiis , H. Iwata , S. A. Hurvitz , S. A. Wander , M. Danso , D. R. Lu , J. Perkins Smith , Y. Liu , L. Tran , S. Anderson , M. Campone , Future Oncol. 2024, 20, 2447. 39072356 10.1080/14796694.2024.2377530 PMC11524203

[103] X. Gao , H. A. Burris, III , J. Vuky , R. Dreicer , A. O. Sartor , C. N. Sternberg , I. J. Percent , M. H. A. Hussain , A. Rezazadeh Kalebasty , J. Shen , E. I. Heath , G. Abesada‐Terk , S. G. Gandhi , M. McKean , H. Lu , E. Berghorn , R. Gedrich , S. D. Chirnomas , N. J. Vogelzang , D. P. Petrylak , J. Clin. Oncol. 2022, 40, 17.

[104] Y. Wang , X. Wang , T. Liu , C. Wang , Q. Meng , F. Meng , J. Yu , J. Liu , Y. Fan , D. Gennert , F. W. Pun , A. Aliper , F. Ren , M. Zhang , X. Cai , X. Ding , A. Zhavoronkov , Nat. Commun. 2025, 16, 10759. 41315240 10.1038/s41467-025-65796-8 PMC12663101

[105] A. Mullard , Nat. Rev. Drug Discov. 2024, 23, 799. 39402425 10.1038/d41573-024-00170-9

[106] A. Mullard , Nat. Rev. Drug Discov. 2021, 20, 247. 33737725 10.1038/d41573-021-00052-4

[107] S. L. Schreiber , Cell 2021, 184, 3.33417864

[108] A. Domostegui , L. Nieto‐Barrado , C. Perez‐Lopez , C. Mayor‐Ruiz , Chem. Soc. Rev. 2022, 51, 5498. 35723413 10.1039/d2cs00197g

[109] M. Cully , Nat. Biotechnol. 2025, 43, 1212. 40739073 10.1038/s41587-025-02779-6

[110] S. Jiang , K. Liu , T. Jiang , H. Li , X. Wei , X. Wan , C. Zhong , R. Gong , Z. Chen , C. Zou , Q. Zhang , Y. Cheng , D. Cao , Nat. Commun. 2025, 16, 7854. 40846852 10.1038/s41467-025-62288-7 PMC12373995

[111] A. S. Ben Geoffrey , D. Agrawal , N. M. Kulkarni , M. Gunasekaran , ACS Omega 2025, 10, 6650. 40028145 10.1021/acsomega.4c08049 PMC11865985

[112] N. N. Islam , T. R. Caulfield , Biomolecules 2025, 15, 849. 40563489 10.3390/biom15060849 PMC12190558

[113] F. J. Prael , J. Cox , N. Sturm , P. Kutchukian , W. C. Forrester , G. Michaud , J. Blank , L. Shen , R. Rodríguez‐Pérez , Artif. Intell. Life Sci. 2024, 6, 100100.

[114] I. Kang , S. Seo , S. Cho , in Proceedings of the American Association for Cancer Research Annual Meeting 2025 Part 1 (Regular Abstracts); **2025**; Chicago, IL. Philadelphia (PA), AACR; Cancer Res 2025;85(8_Suppl_1), Abstract nr 384.

[115] J. A. Izaguirre , Y. Wu , Z. McDargh , T. Palpant , A. M. Razavi , F. Trovato , C. Koh , H. Xu , bioRxiv 2025, 2025, 01.13.632817.

[116] L. Márquez Cantudo , C. V. López‐Gallego , C. Coderch Boué , B. Pascual‐Teresa Fernández , ANALES RANF 2023, 89, 265.

[117] B. Huang , M. Abedi , G. Ahn , B. Coventry , I. Sappington , C. Tang , R. Wang , T. Schlichthaerle , J. Z. Zhang , Y. Wang , I. Goreshnik , C. W. Chiu , A. Chazin‐Gray , S. Chan , S. Gerben , A. Murray , S. Wang , J. O’Neill , L. Yi , R. Yeh , A. Misquith , A. Wolf , L. M. Tomasovic , D. I. Piraner , M. J. Duran Gonzalez , N. R. Bennett , P. Venkatesh , M. Ahlrichs , C. Dobbins , W. Yang , X. Wang , D. D. Sahtoe , D. Vafeados , R. Mout , S. Shivaei , L. Cao , L. Carter , L. Stewart , J. B. Spangler , K. T. Roybal , P. Greisen, Jr. , X. Li , G. J. L. Bernardes , C. R. Bertozzi , D. Baker , Nature 2024, 638, 796. 39322662 10.1038/s41586-024-07948-2 PMC11839401

[118] Y. Jin , X. Liao , J. Zhang , H. Duan , R. Xu , X. Luo , X. Yu , B. Li , J. Wang , X. Li , C. Li , L. Xu , L. Li , Y. Lu , G. Cai , Z. Zhou , S. Zeng , W. Huang , J. Li , Y. Zhou , X. Dong , J. Che , J. Med. Chem. 2025, 68, 21249. 41086092 10.1021/acs.jmedchem.5c01159

[119] F. J. Prael Iii , J. Blank , W. C. Forrester , L. Shen , R. Rodríguez‐Pérez , Artif. Intell. Life Sci. 2025, 7, 100121.

[120] C. Radice , K. Korzekwa , S. Nagar , Drug Metab. Dispos. 2022, 50, 750. 35339986 10.1124/dmd.122.000831 PMC9199116

[121] R. Guo , F. Yang , E. C. Cherney , RSC Med. Chem. 2025, 16, 1865. 39949641 10.1039/d4md00718b PMC11815867

[122] B. Mostofian , H.‐J. Martin , A. Razavi , S. Patel , B. Allen , W. Sherman , J. A. Izaguirre , J. Chem. Inf. Model. 2023, 63, 5408. 37602861 10.1021/acs.jcim.3c00603 PMC10498452

[123] G. Kudo , T. Hirao , R. Harada , Y. Shigeta , T. Hirokawa , R. Yoshino , J. Chem. Inf. Model. 2025, 65, 6939. 40550492 10.1021/acs.jcim.5c00102 PMC12264966

[124] T. Dixon , D. MacPherson , B. Mostofian , T. Dauzhenka , S. Lotz , D. McGee , S. Shechter , U. R. Shrestha , R. Wiewiora , Z. A. McDargh , F. Pei , R. Pal , J. V. Ribeiro , T. Wilkerson , V. Sachdeva , N. Gao , S. Jain , S. Sparks , Y. Li , A. Vinitsky , X. Zhang , A. M. Razavi , I. Kolossváry , J. Imbriglio , A. Evdokimov , L. Bergeron , W. Zhou , J. Adhikari , B. Ruprecht , A. Dickson , H. Xu , W. Sherman , J. A. Izaguirre , Nat. Commun. 2022, 13, 5884. 36202813 10.1038/s41467-022-33575-4 PMC9537307

[125] K. Zhang , X. Yang , Y. Wang , Y. Yu , N. Huang , G. Li , X. Li , J. C. Wu , S. Yang , Nat. Med. 2025, 31, 45. 39833407 10.1038/s41591-024-03434-4

[126] H. Xu , K. M. J. Shuttleworth , Intell. Med. 2024, 4, 52.

[127] R. Alizadehsani , S. S. Oyelere , S. Hussain , S. K. Jagatheesaperumal , R. R. Calixto , M. Rahouti , M. Roshanzamir , V. H. C. De Albuquerque , IEEE Access 2024, 12, 35796.

[128] V. Hassija , V. Chamola , A. Mahapatra , A. Singal , D. Goel , K. Huang , S. Scardapane , I. Spinelli , M. Mahmud , A. Hussain , Cognit. Comput. 2023, 16, 45.

[129] C. N. Hayes , H. Nakahara , A. Ono , M. Tsuge , S. Oka , Genes 2024, 15, 1551. 39766818 10.3390/genes15121551 PMC11675490

[130] M. Krassowski , V. Das , S. K. Sahu , B. B. Misra , Front. Genet. 2020, 11, 610798. 33362867 10.3389/fgene.2020.610798 PMC7758509

[131] Z. Yang , R. Kotoge , X. Piao , Z. Chen , L. Zhu , P. Gao , Y. Matsubara , Y. Sakurai , J. Sun , Sci. Data 2025, 12, 913. 40447627 10.1038/s41597-025-05235-x PMC12125382

[132] L. K. Nguyen , Brief. Bioinform. 2016, 17, 479. 26210356 10.1093/bib/bbv052

[133] B. L. Puniya , J. Mol. Biol. 2025, 437, 169181. 40316010 10.1016/j.jmb.2025.169181

[134] Y. A. Qadri , S. Shaikh , K. Ahmad , I. Choi , S. W. Kim , A. V. Vasilakos , Pharmaceutics 2025, 17, 1119. 41012456 10.3390/pharmaceutics17091119 PMC12472777

[135] I. Ponzoni , J. A. Páez Prosper , N. E. Campillo , WIREs Comput. Mol. Sci. 2023, 13, e1681.

[136] R. Brighenti , M. P. Cosma , J. Mech. Phys. Sol. 2020, 143, 104011.

[137] P. C. Tiwari , R. Pal , M. J. Chaudhary , R. Nath , Drug Dev. Res. 2023, 84, 1652. 37712494 10.1002/ddr.22115

[138] Y. Zhou , P. Geng , S. Zhang , F. Xiao , G. Cai , L. Chen , For the Alzheimer’s Disease Neuroimaging Initiative , Q. Lu , Briefings Bioinf. 2024, 25, bbae448.

[139] M. Hou , M. Li , Y. Li , X. Wu , D. Long , D. Sun , J. Zeng , Bioorg. Med. Chem. 2025, 129, 118297. 40614626 10.1016/j.bmc.2025.118297

[140] F. Boniolo , E. Dorigatti , A. J. Ohnmacht , D. Saur , B. Schubert , M. P. Menden , Expert Opin. Drug Discov. 2021, 16, 991. 34075855 10.1080/17460441.2021.1918096

[141] P. Wludyka , Technometrics 2020, 62, 561.

[142] M.‐E. Martel , A. José‐Garcia , C. Vens , M. De Vos , V. Sobanski , Therapies 2025, 81, 171. 10.1016/j.therap.2025.10.003 41167996

[143] O. Eladl , Biochem. Pharmacol. 2025, 242, 117297. 40907798 10.1016/j.bcp.2025.117297

